# Extensive data mining uncovers novel diversity among members of the rare biosphere within the Thermoplasmatota

**DOI:** 10.1186/s40168-025-02140-8

**Published:** 2025-07-01

**Authors:** Mara D. Maeke, Xiuran Yin, Lea C. Wunder, Chiara Vanni, Tim Richter-Heitmann, Samuel Miravet-Verde, Hans-Joachim Ruscheweyh, Shinichi Sunagawa, Jenny Fabian, Judith Piontek, Michael W. Friedrich, Christiane Hassenrück

**Affiliations:** 1 Microbial Ecophysiology Group, Faculty of Biology/Chemistry, University of Bremen, Bremen, 28359 Germany; 2 State Key Laboratory of Marine Resource Utilization in South China Sea, Hainan University, Haikou, 570228 China; 3 MARUM – Center for Marine Environmental Sciences, University of Bremen, Bremen, 28359 Germany; 4 Department of Biology, Institute of Microbiology and Swiss Institute of Bioinformatics, ETH Zurich, Zurich, 8093 Switzerland; 5 Biological Oceanography, Leibniz Institute for Baltic Sea Research Warnemünde (IOW), Rostock, 18119 Germany

## Abstract

**Background:**

Rare species, especially of the marine sedimentary biosphere, have long been overlooked owing to the complexity of sediment microbial communities, their sporadic temporal and patchy spatial abundance, and challenges in cultivating environmental microorganisms. In this study, we combined enrichments, targeted metagenomic sequencing, and extensive data mining to uncover uncultivated members of the archaeal rare biosphere in marine sediments.

**Results:**

In protein-amended enrichments, we detected the ecologically and metabolically uncharacterized class *Candidatus* Penumbrarchaeia within the phylum Thermoplasmatota. By screening more than 8000 metagenomic runs and 11,479 published genome assemblies, we expanded the phylogeny of *Ca*. Penumbrarchaeia by 3 novel orders. All six identified families of this class show low abundance in environmental samples characteristic of rare biosphere members. Members of the class *Ca*. Penumbrarchaeia were predicted to be involved in organic matter degradation in anoxic, carbon-rich habitats. All *Ca*. Penumbrarchaeia families contain high numbers of taxon-specific orthologous genes, highlighting their environmental adaptations and habitat specificity. Besides, members of this group exhibit the highest proportion of unknown genes within the entire phylum Thermoplasmatota, suggesting a high degree of functional novelty in this class.

**Conclusions:**

In this study, we emphasize the necessity of targeted, data-integrative approaches to deepen our understanding of the rare biosphere and uncover the functions and metabolic potential hidden within these understudied taxa.

Video Abstract

**Supplementary Information:**

The online version contains supplementary material available at 10.1186/s40168-025-02140-8.

## Introduction

In the environment, the vast majority of microbial species is represented by low abundance microorganisms, known as the “rare biosphere” [1]. While many studies define rare taxa as those being less than 0.01–0.1% abundant in a sample at a specific time point [2, 3], rarity is not only confined to population sizes but can also be measured by geographic range and habitat specificity [4, 5]. Rare taxa are hypothesized to play an important role in ecosystems by carrying a gene pool, which can be accessed under changing environmental conditions, functioning as a seed bank, or by supporting the community with key functions [6, 7]. These functions can be nutrient cycling [8–10], degradation of pollutants [11], or the promotion of community resilience [12, 13]. Due to high intra- or interspecific competition [6], as well as the assumed limited environmental distribution most rare taxa occupy, these taxa mostly show only temporally and spatially constrained abundance [7], making the study of the rare biosphere challenging.

Most studies so far conducted on the rare marine biosphere have focused on diversity assessments of bacterial and archaeal plankton using high-throughput 16S rRNA gene surveys [2, 3, 14–20]. More recently, first metagenomic approaches have been applied to investigate the rare biosphere in marine bacterioplankton [21]. However, the rare biosphere in marine sediments remains largely unexplored, owing to the complexity of sediment communities [22, 23], the sequencing effort needed to resolve such complex communities, computational resources, and associated costs.

To reduce the complexity of sediment communities and thereby make the rare biosphere more accessible for the analysis of metabolic functions, enrichment experiments with substrate amendments were designed to selectively promote the growth of rare taxa [24–27]. While isolation would be preferable to further characterize the metabolic capabilities of rare taxa, most microorganisms remain uncultured despite recent advances in cultivation techniques [28, 29]. Therefore, enrichment techniques coupled to metagenome analyses simplify the description of the full metabolic potential of rare uncultivated microorganisms in the absence of isolates [2, 29–31]. Yet, even the combination of these methods only provides a snapshot of the enriched taxa at a specific time point in a specific setting and cannot offer further information regarding their global diversity or habitat selection.

Recent advances in high-throughput sequencing and computational techniques have enabled deep sequencing of microbial communities, including the rare biosphere [2, 32–34] and led to rapid data accumulation on public databases. Data from next-generation sequencing runs deposited on the Sequence Read Archive (SRA) under the umbrella of the International Nucleotide Database Collaboration (INSDC) reached a data volume of 57.9 PB in 2024 [35], while the number of assembled genomes exceeds 2.3 million [36]. Through the screening and recovery of novel metagenome-assembled genomes (MAGs) and single-cell amplified genomes (SAGs) from public archives by data-driven projects, such as the Genome Taxonomy Database (GDTB) [37], the Genomes from Earth’s Microbiomes (GEM) [38], and the Ocean Microbiomics Database [39], the catalog of microbial diversity is steadily increasing. Genomic analyses conducted on unidentified microorganisms disclosed novel functions and illustrated the extent of their untapped metabolic potential [38–42]. These studies attest to the wealth of information available in existing data on public archives. Despite the evidence outlined above for the importance of the rare biosphere, rare taxa remain regularly overlooked in environmental metagenomic studies, since the most abundant taxa remain the focus of the research on key players in microbial communities [6]. Still, the generated sequencing data may hold valuable information about the phylogenetic and metabolic diversity of rare taxa, their habitats, and their role in the Earth’s environments, which can be accessed by group-targeted data mining of the SRA.

Archaea, in particular, have been regarded as members of the rare biosphere, complicating the study of their diversity and role in biogeochemical cycles [34, 43]. While it has been estimated that half of the archaeal diversity remains unidentified, multiple archaeal groups were shown to play an important role in organic matter degradation [34, 44, 45]. To investigate the capabilities of uncultivated archaea involved in the degradation of organic matter, specifically proteins, we established enrichments amended with pure egg-white protein (further referred to as protein). After detecting a so far ecologically and metabolically uncharacterized class of the phylum Thermoplasmatota within these protein-amended enrichments, we used the data available on the databases of the INSDC to conduct extensive group-targeted data mining to describe this new class. By screening 11,479 publicly available genome assemblies, data from 8287 metagenomic sequencing runs, and the genomes of the Ocean Microbiomics Database (OMDB), we generated an integrative dataset enabling the study and formal description of this new class. We investigated the phylogenetic diversity, biogeography, and metabolic capabilities of the novel class and presented three previously unknown orders within this group. Notably, by analyzing the metabolic potential within the novel class, we observed percentages of unknown genes higher than those found in any other class within the Thermoplasmatota.

## Results

### High abundance of an ecologically and metabolically uncharacterized class of the phylum Thermoplasmatota in protein enrichments

**Fig. 1:**
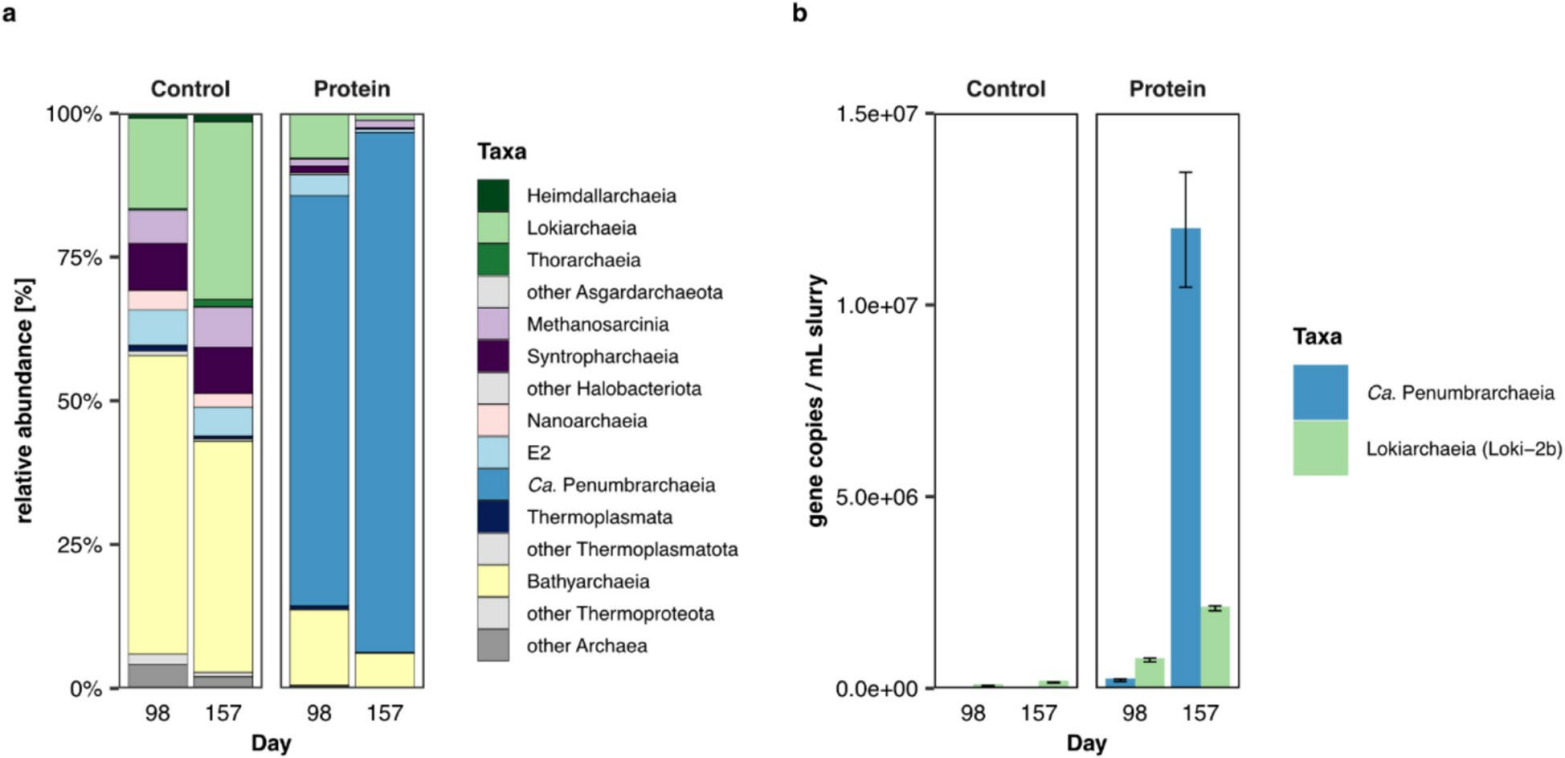
Abundance of archaeal 16S rRNA genes in second-generation enrichments on days 98 and 157. **a** Relative 16S rRNA gene abundance within protein samples (amended with protein, sulfate, and antibiotics) on day 98 and day 157 and control samples (amended with sulfate and antibiotics). **b** 16S rRNA gene copies per ml slurry of the classes *Ca.* Penumbrarchaeia and Lokiarchaeia subgroup Loki-2b in protein-amended samples and control samples

To investigate the potential of novel microorganisms for protein degradation, we established a series of slurry enrichments using sediment from the Helgoland mud area. In order to reduce and eventually eliminate the sediment component, incubations were transferred into anoxic Widdel medium after 372 days for further enrichment (“Further enrichment of target microorganisms” section). Samples were amended with protein, sulfate, and an antibiotic mix, supplied at the beginning of the incubation experiment, to specifically target archaeal groups by inhibiting general bacterial groups in marine sediments. Subsamples of the second-generation enrichment were taken on days 98 (480 days total) and 157 (529 days total) to perform archaeal 16S rRNA gene amplicon sequencing and qPCR. Within one replicate incubation, amended with protein, sulfate, and antibiotics, we observed a high relative abundance of EX4484-6 (90.4%), a so far undescribed class within the phylum Thermoplasmatota, which had increased from day 98 to day 157 (Fig. 1a, Table S1). The taxon was represented by four ASVs, of which one ASV showed a relative abundance of 90.2% (Fig. S1b, Table S1). Hereafter, the class EX4484-6 will be referred to as *Candidatus* Penumbrarchaeia (proposal of type material and higher ranks in Supplementary material). In contrast, a second replicate showed a high relative abundance of ‘*Candidatus* Prometheoarchaeum syntrophicum’ strain MK-D1 on day 157 (86.56%), while the relative abundance of the *Ca*. Penumbrarchaeia class was lower (12%) (Fig. S1a and c, Table S1, Results SI). The control samples without protein amendment showed high relative abundances in Bathyarchaeia and Lokiarchaeia, reaching up to 52% and 31%, respectively. The class *Ca*. Penumbrarchaeia was not enriched in any of the control samples.

Using a newly designed qPCR primer set, specific for the archaeal class *Ca*. Penumbrarchaeia, we followed the abundance of its 16S rRNA gene copies within the same enrichments. In the unenriched environmental samples and in control samples, gene copies of the class *Ca*. Penumbrarchaeia were below the detection limit (Fig. 1b), providing the first indication that in the environment this group is a member of the rare biosphere. However, in the protein-amended samples, the 16S rRNA gene copies of the class *Ca*. Penumbrarchaeia increased 100-fold from day 98 to day 157, reaching a number of 1.2 × 10^7^ gene copies per ml slurry (Fig. 1b, day 157). After 372 days (744 days total), gene copies decreased again (8.7 × 10^5^ gene copies per ml slurry; Fig. S2). Based on these initial findings, the uncultivated class *Ca*. Penumbrarchaeia might be involved in protein metabolism. Metagenomic sequencing was performed to further analyze the metabolic potential of this class.

### Data mining uncovers 35 MAGs representing the class *Ca.* Penumbrarchaeia

Through metagenomic sequencing of the protein and antibiotics amended enrichment from day 157, we retrieved one MAG classified as a member of the class *Ca*. Penumbrarchaeia. To analyze the phylogeny between the 16S rRNA gene obtained from the *Ca*. Penumbrarchaeia MAG and the highly abundant *Ca*. Penumbrarchaeia ASV obtained through 16S rRNA gene amplicon sequencing, we calculated a 16S rRNA gene phylogenetic tree (Fig. S3a). Both the single copy of the full-length 16S rRNA gene from the MAG and the 16S rRNA gene sequence of the ASV fell into the same branch with a sequence identity of 100%. A nucleotide blast search against the rRNA/ITS databases of NCBI (date accessed 19.03.2024) [46] revealed that the closest cultured representative of both sequences was *Methanomassiliicoccus luminyensis*, with a sequence identity of 83–84% and 100% of the 16S rRNA gene sequence of the ASV and 97% of the 16S rRNA gene from the MAG being covered (Table S2).

**Fig. 2:**
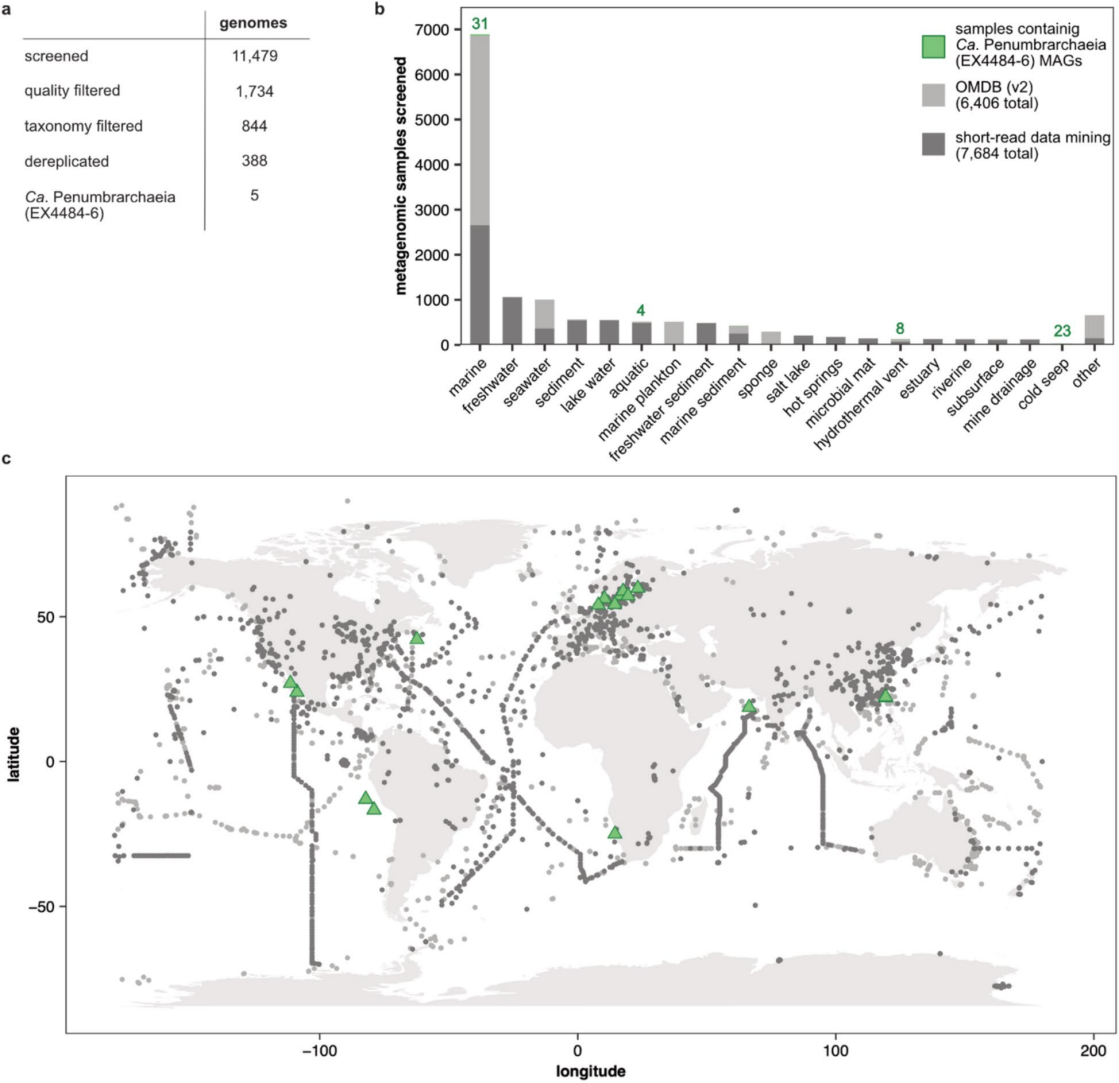
Screened data for retrieving novel *Ca.* Penumbrarchaeia MAGs. Number of datasets of **a** screened published MAGs from GenBank and **b** screened metagenomic samples from the SRA, the OMDB (v2), and those from which *Ca*. Penumbrarchaeia MAGs were reconstructed. Numbers above bars indicate the number of samples in which *Ca*. Penumbrarchaeia was detected and ultimately reconstructed. The category “other” includes samples of 35 additional environmental categories, for which < 100 metagenomic samples were available, none of which *Ca*. Penumbrarchaeia was detected in (e.g., algae, alkali sediment, viral, hypolithon, or coral reef metagenomes). **c** World map of all screened metagenomic samples derived through short-read data mining and the OMDB (v2) with an indication of those locations at which target MAGs were detected

To further characterize the novel class, we performed a group-targeted data mining, investigating the phylogenetic breadth, biogeography, abundance, and rarity in other habitats, as well as the metabolic capabilities. We increased the number of available genomes for the class *Ca*. Penumbrarchaeia by searching 11,479 genome assemblies of the phylum Thermoplasmatota and additional unclassified genome assemblies (Fig. 2a) published in GenBank. Still, we could only find an additional five published *Ca*. Penumbrarchaeia MAGs, prompting us to screen 57.8 TB of publicly available metagenomic short-read data. Using the nonredundant marine *Ca*. Penumbrarchaeia MAGs retrieved through the first screening, we searched 8287 publicly available metagenomic runs (of 7684 metagenomic samples) of aquatic origin for the presence of *Ca*. Penumbrarchaeia MAGs (Fig. 2b, c). Further including data retrieved from the OMDB (v2), sampling bias was reduced by screening samples of in total 54 different categories, covering coastal and open ocean environments, along with extreme habitats known to host Thermoplasmatota [47–51] (Fig. 2b). In 30 metagenomic studies, the class *Ca*. Penumbrarchaeia was found with a cumulative coverage > 2 (Table S3). Medium- and high-quality *Ca*. Penumbrarchaeia MAGs as per the minimum information about a metagenome-assembled genome (MIMAG) [52] could be reconstructed from the studies PRJNA368391, PRJNA531756, PRJNA541421, PRJNA704804, PRJNA721298, and PRJNA889212 and were added to our dataset. Further, previously unpublished *Ca*. Penumbrarchaeia MAGs from the Baltic Sea, Benguela Upwelling System (Namibia), Cariaco Basin (Venezuela), Arabic Sea, Qinghai Lake (China), and the Scotian Slope (Atlantic Ocean), were added to our dataset (Table S4), yielding a total of 35 medium- to high-quality *Ca*. Penumbrarchaeia MAGs with a completeness of > 80% (80.3 to 97.9%) and contamination of < 5% (0.05 to 4.35%). Genome sizes of the single MAGs were adjusted based on their completeness and varied from the smallest genome size of 1.14 Mbp to the largest genome size of 3.94 Mbp (Table S5).

### *Ca.* Penumbrarchaeia forms a novel class with four orders

**Fig. 3:**
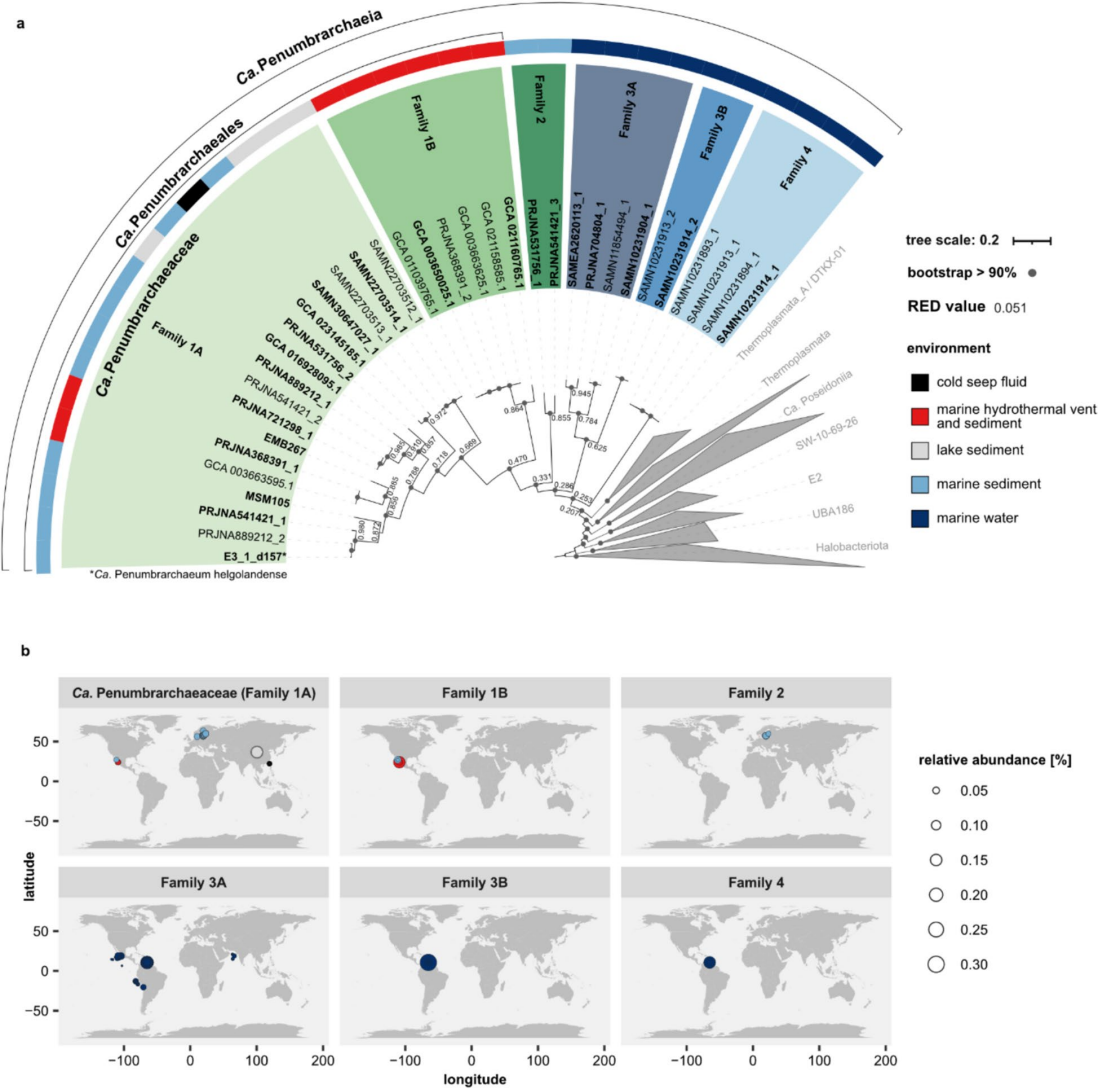
Phylogenomic tree of the Thermoplasmatota based on 53 archaeal marker genes. **a** Maximum-likelihood tree (RAxML, 100 bootstraps) of 370 Thermoplasmatota MAGs and 35 *Ca*. Penumbrarchaeia MAGs obtained through data mining of genome assemblies and metagenomic short-read datasets. MAGs in bold define the nonredundant *Ca*. Penumbrarchaeia MAGs, derived through MAG dereplication (“Retrieval of EX4484-6 MAGs from public archives” section). Node labels indicate RED values, which were used to define the phylogeny of the class *Ca*. Penumbrarchaeia into four orders, consisting of six families (1 A (*Ca*. Penumbrarchaeia), 1B, 2, 3 A, 3B, 4). The environments from which single MAGs are derived are indicated by a colored strip. **b** Relative abundance of nonredundant *Ca*. Penumbrarchaeia representative MAGs in the environment estimated from metagenomic short-reads mapped against a competitive reference and quantified using coverm. A more detailed description is provided in the main “Relative MAG abundance in the environment” section. The environments individual MAGs were found in are indicated by color

Next, we performed a phylogenomic analysis on 370 Thermoplasmatota species found within the screened 11,479 genome assemblies and all 35 *Ca*. Penumbrarchaeia MAGs. The tree based on 53 archaeal marker genes showed one distinct cluster with high bootstrap support, consisting of all 35 *Ca*. Penumbrarchaeia MAGs (Fig. 3a). Taxonomic ranks were assigned to the *Ca*. Penumbrarchaeia MAGs using the relative evolutionary divergence (RED) rank normalization according to GTDB. Based on the calculated RED [37], the *Ca*. Penumbrarchaeia cluster can be grouped into four orders (orders 1–4) (Fig. S4), which consist of a total of six families (order 1: family 1 A, 1B; order 2: family 2; order 3: family 3 A, 3B; order 4: family 4) (for further information, see Supplementary results II). From these, only families 1 A and 1B were represented in the GTDB database versions 207 and 214. Additional computed average nucleotide identity (ANI) and amino acid identity (AAI) supported the classification according to reported RED values (Figs. S5 and S6). The phylogeny was later also confirmed by a species tree based on the core genome (“Annotation of EX4484-6 MAGs” section) of the class *Ca*. Penumbrarchaeia (Fig. S3b).

### *Ca.* Penumbrarchaeia is globally distributed in coastal and organic-rich areas

We aimed to further gain insights into the biogeography of the *Ca*. Penumbrarchaeia MAGs and differences between the habitats of individual families within this class. For this, 8573 metagenomic runs were mapped against a competitive mapping index that included 20 nonredundant *Ca*. Penumbrarchaeia representatives, and relative abundances of MAGs in these samples were calculated. From all searched metagenomic sequencing runs, *Ca*. Penumbrarchaeia MAGs could only be detected in 128 runs (1.49% of all searched runs; Tables S6 and 7). Three of the nonredundant *Ca*. Penumbrarchaeia MAGs (MSM105, EMB267, GCA_016928095.1) were not found in any of the searched runs. As we used stringent detection methods with a minimum breadth of coverage of 50% and a minimum percentage identity of 95% for computation of relative abundances to avoid false positives, it is possible that these three MAGs were not detected because of false negatives or because their specific environment of preference was not sufficiently well represented in our selection of metagenomic runs.

**Fig. 4:**
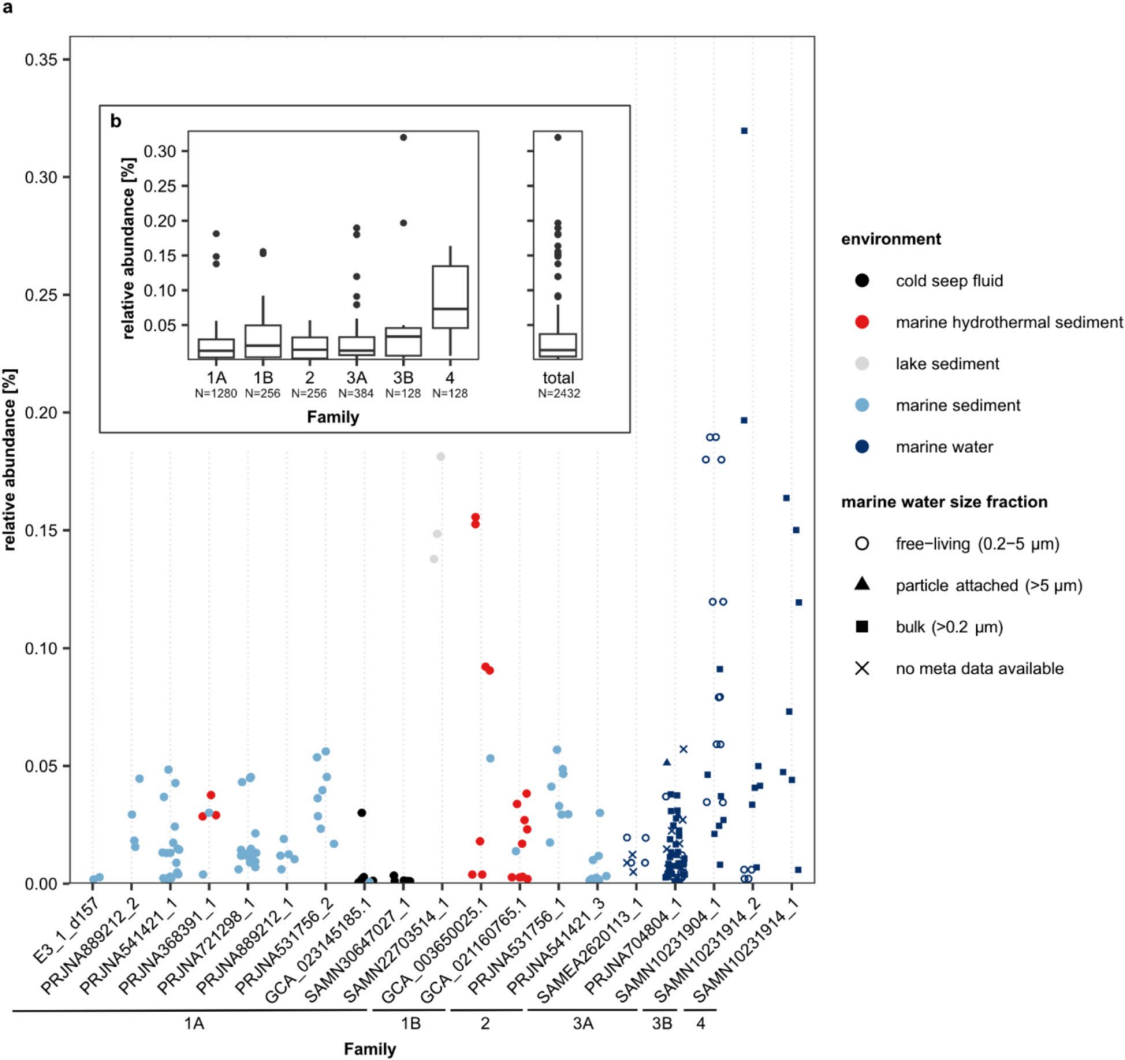
Relative abundances of *Ca*. Penumbrarchaeia MAGs in screened samples for **a** individual nonredundant MAGs. For MAGs found in marine water samples, the filter size each sample was filtered through is indicated by shape. Colors indicate the environment of the samples in which the MAGs were found. **b** Relative abundance of *Ca.* Penumbrarchaeia MAGs summarized by family and for the class as a whole. Family 1 A corresponds to *Ca*. Penumbrarchaeia

Relative abundances of *Ca*. Penumbrarchaeia MAGs in the environment ranged between 0% and a maximum of 0.32%, with on average 0.031% (Figs. 3b and 4, Fig. S7). While at the class level *Ca*. Penumbrarchaeia could be detected in continental shelf environments worldwide (Fig. S7), single families preferred very distinctive habitats (Figs. 3b and 4). MAGs of the families 1 A and 2 were mostly detected in marine surface sediments (Table S7). Family 1 A was additionally present in a variety of environments, such as lake and marine sediments, hydrothermal vents, and cold seeps, and therefore could be found more globally distributed (Figs. 3b and 4). The MAGs of family 2 were present in marine sediments from the Baltic Sea (Fig. 3b). MAGs of family 1B were only found in hydrothermal sediments from the Guaymas and Pescadero basins (Fig. 3b). All MAGs from the families 3 A, 3B, and 4 were found in the marine water column, specifically in oxygen minimum and -deficient zones (Figs. 3b and 4). MAGs of families 3B and 4 were confined to the Cariaco Basin (Venezuela), while family 3 A was additionally observed in the Arabic Sea and off the coasts of Mexico, Peru, and Chile (Fig. 3b). Two of the MAGs originating from the water column (SAMEA2620113_1 and SAMN10231904_1) were found predominantly in the free-living fraction of the seawater community (retained on filters with a pore size 0–5 µm), suggesting a habitat selection of these microorganisms (Fig. 4a). However, most other MAGs found within the water column were derived from bulk seawater samples (retained on filters with 0.2-µm pore size); thus, it cannot be resolved whether they were part of the free-living or particle-associated community (Fig. 4a). Compared to other classes within the phylum of Thermoplasmatota, the class *Ca*. Penumbrarchaeia showed the lowest average relative abundance in the environment (Fig. S8b). The highest relative abundances among the Thermoplasmatota were found in the classes Thermoplasmata (up to 43%) and *Ca*. Poseidonia (up to 6%).

### Housekeeping genes and shared metabolic potential encoded by the core genome of the class *Ca.* Penumbrarchaeia

To assess genomic differences among families, orthogroups shared between MAGs of the class *Ca*. Penumbrarchaeia were studied. Orthogroups are defined as a set of genes, which descended from a single last common ancestor [53], and as such are considered to be homologous [54]. For this, we performed an NMDS analysis based on orthogroups found within all *Ca*. Penumbrarchaeia MAGs (Fig. S9a). The NMDS showed a clustering of MAGs, which resembled the structure of the phylogenomic tree (Fig. 3a) with MAGs derived from sediment samples and those derived from water samples forming two well-defined distinct clusters. Generally, the number of orthogroups found per family resembled differences in genome sizes within the families (Fig. S9b, Table S5). Three of the families contained high numbers of family-specific orthogroups. In family 1 A, with the highest number of orthogroups (*n* = 2961), 1179 family-specific orthogroups (40% of the orthogroups in this family) could be identified (Fig. S9b). Families 3 A and 4 from the water column contained 848 (32%) and 800 (40%) family-specific orthogroups, respectively. We further observed that more closely related families within the class *Ca*. Penumbrarchaeia shared more orthogroups (Fig. S10). In total, we found 477 orthogroups, which were present in at least 90% of all 35 *Ca*. Penumbrarchaeia MAGs, and as such were defined as the core genome of the class *Ca*. Penumbrarchaeia (Table S8). Overall, the core genome encoded expected housekeeping genes involved in gene expression, such as translation, transcription, replication, DNA repair, tRNA biogenesis, and ribosomal proteins. Further, genes affiliated with metabolic processes, such as transporters, the gluconeogenesis pathway, reverse TCA (rTCA) cycle, pyruvate metabolism, fatty acid, and amino acid degradation, were found within the core genome (Tables S8, S9, S10, 11). Moreover, genes needed for biosynthetic processes, such as the nucleotide and amino sugar metabolism as well as the biosynthesis of amino acids, fatty acids, lipids, glycans, vitamins, and cofactors, were encoded (Tables S8, S9, 10).

### Heterotrophy and mixotrophy as main nutritional strategies of the class *Ca.* Penumbrarchaeia

**Fig. 5:**
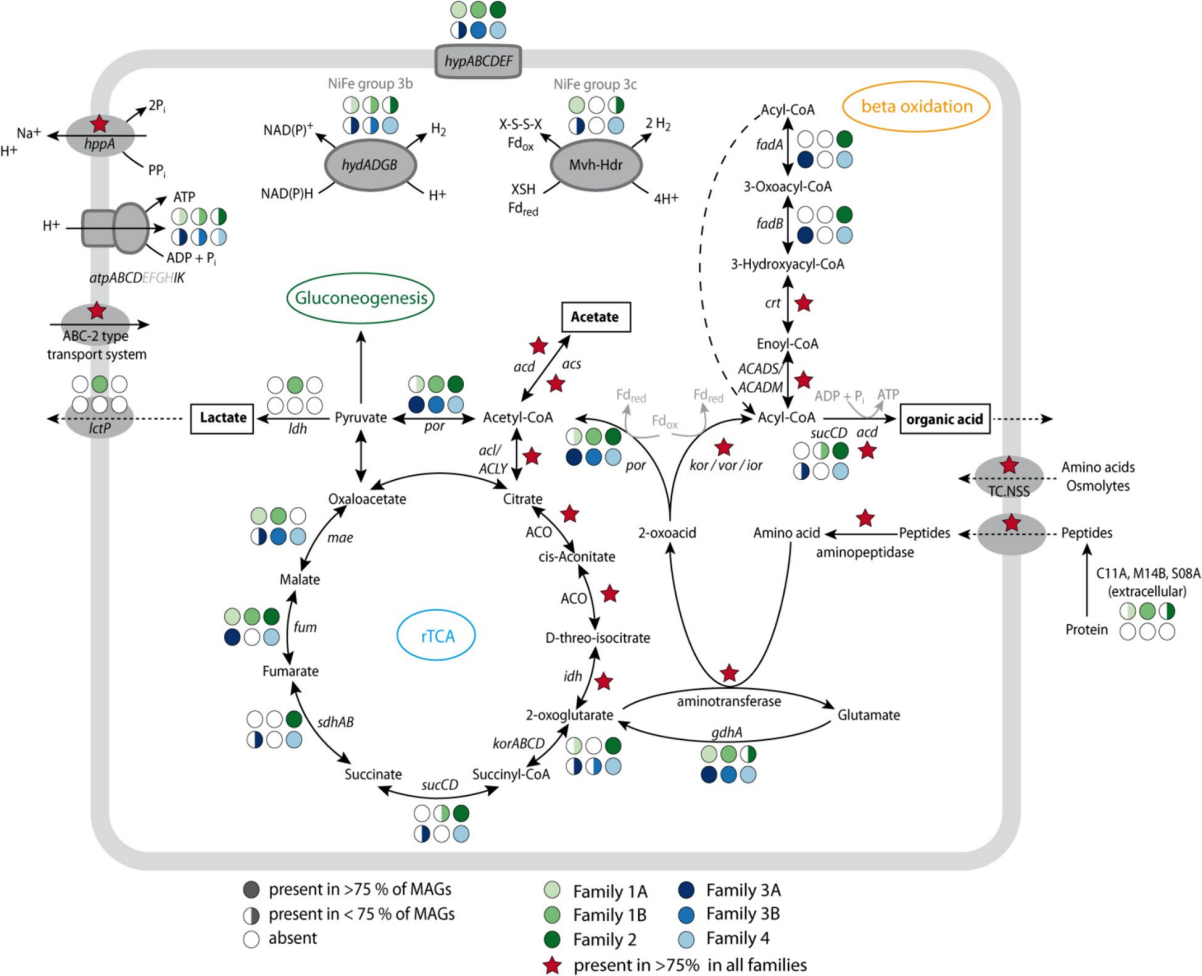
Metabolic reconstruction of the main metabolic features of the novel class *Ca*. Penumbrarchaeia. The presence of genes is indicated by full or half circles for each family or with red stars if present in > 75% of the MAGs of all families. Amino acid degradation is as follows: *gdhA* glutamate dehydrogenase, *kor* 2-oxoacid:ferredoxin oxidoreductase, *vor* 2-oxoisovalerate ferredoxin oxidoreductase, *ior* indolepyruvate ferredoxin oxidoreductase, *por* pyruvate ferredoxin oxidoreductase, and *acd* acetyl coenzyme A synthetase; beta-oxidation: ACADS butyryl-CoA dehydrogenase, ACADM acyl-CoA dehydrogenase, *crt* enoyl-CoA hydratase, *fadB* 3-hydroxyacyl-CoA dehydrogenase, and *fadA* acetyl-CoA acyltransferase; rTCA: *acl*/ACLY ATP-citrate lyase, ACO aconitate hydratase, *idh* isocitrate dehydrogenase, *korABCD* 2-oxoacid:ferredoxin oxidoreductase, *sucCD* succinyl-CoA synthetase, *sdhAB* succinate dehydrogenase/fumarate reductase, *fum* fumarate hydratase, and *mae* malate dehydrogenase (oxaloacetate-decarboxylating); pyruvate metabolism: *acd* acetyl coenzyme A synthetase, *acs* acetyl-CoA synthetase, *por* pyruvate ferredoxin oxidoreductase, and *ldh* lactate dehydrogenase; and hydrogenases: *hydADGB* sulfhydrogenase, *mvh* F420-non-reducing hydrogenase, *hdr* heterodisulfide reductase, *hypABCDEF* hydrogenase expression/formation protein, *atpABCDEFGHJK* V/A-type H + -transporting ATPase, *lctP* lactate permease, *hppA* K(+)-stimulated pyrophosphate-energized sodium pump, and TC.NSS neurotransmitter:Na + symporter family

As we found our initial *Ca*. Penumbrarchaeia MAG within protein-amended enrichments, we analyzed all MAGs for the potential of protein degradation (Table S11). Metabolic capabilities included amino acid degradation for all MAGs of this novel class (for details, see Fig. 5). MAGs of the families 1 A, 1B, and 2 additionally encoded genes for extracellular peptidases of the families C11 A, M14B, and S08 A (Fig. 5, Fig. S11a), which are required for the first step of protein polymer hydrolysis into smaller peptides, whereas families 3 A, 3B, and 4 lacked genes for these enzymes. However, MAGs of all families encoded genes for oligopeptide transporters, different aminopeptidases (*pepF*, *pepT*, *pepP*, *pepS*, *map*), and aminotransferases (Table S12, Fig. S11b). Furthermore, all MAGs encoded aspartate aminotransferase (*aspB*) and alanine aminotransferase (*alaA*) for deamination. Single MAGs encoded an alanine-glyoxylate transaminase (AGXT2), branched-chain amino acid aminotransferase (*ilvE*), and aromatic amino acid aminotransferase. After deamination, the resulting 2-oxoacids can be further converted to acetyl-CoA via pyruvate ferredoxin oxidoreductase (*por*) or to acyl-CoA via indolepyruvate ferredoxin oxidoreductase (*ior*), 2-oxoacid:ferredoxin oxidoreductase (*kor*), or 2-oxoisovalerate ferredoxin oxidoreductase (*vor*). Energy-rich acyl-CoA can support ATP formation by an acetyl coenzyme A synthetase (*acdAB*; ADP-forming), which was found in all orders. In four families (1B, 2, 3 A, and 4), also genes for succinyl-CoA synthetase (*sucCD*) were also found. The amino acid degradation results in the main products acetate and organic acids.

The families 2, 3 A, and 4 additionally encoded all genes of the beta-oxidation pathway, including a butyryl-CoA dehydrogenase (ACADS), acyl-CoA dehydrogenase (ACADM), enoyl-CoA hydratase (*crt*), 3-hydroxyacyl-CoA dehydrogenase (*fadB*), and acetyl-CoA acyltransferase (*fadA*) to further degrade short- and medium-chain acyl-CoAs. Family 1B additionally contained a lactate dehydrogenase (*ldhA*), which forms lactate from pyruvate. The findings of protein, amino acid, and fatty acid degradation among the families of the *Ca*. Penumbrarchaeia suggest a heterotrophic lifestyle for this class. Additionally, all families contained genes encoding the partial rTCA cycle. Moreover, the presence of genes of the Wood-Ljungdahl pathway in family 3 A could indicate a mixotrophic lifestyle for this family. A more detailed annotation of metabolic and assimilatory pathways can be found in supplementary results for all orders (Results SIII, Figs. S12, S13, S14, S15, Tables S10, S11, S12).

### *Ca.* Penumbrarchaeia families are adapted to environmental stress

MAGs of all families encoded genes for the prevention of oxidative stress, including thioredoxin reductase (*trxR*), thioredoxin (*trxA*), and desulfoferredoxin (*dfx*), acting as superoxide reductase [55]. Moreover, in MAGs of all families, except family 4, genes for the conversion of hydrogen peroxide to water, catalyzed by peroxiredoxin (*prxQ*), were found [56]. MAGs of families 1 A and 2 further contained genes for the reduction of arsenate via an arsenate reductase (*arsC*), which reduces arsenate As(V) to arsenite As(III). For the removal of arsenite from the cell, a gene encoding an arsenite transporter (*acr3*/*arsB*) was found in MAGs of families 1 A, 2, and 3 A. An arsenite methyltransferase (AS3MT) was additionally found in the MAGs of families 1 A, 3 A, 3B, and 4. Lastly, a gene for the defense against antimicrobial drugs was encoded in all MAGs of families 1 A, 2, and 4, annotated as a MATE family drug/sodium antiporter, which is driven by a sodium gradient [57].

### Rare MAGs in the phylum Thermoplasmatota hold higher numbers of genes encoding functionally unknown genes

Defining protein-coding genes without a known functional annotation as either hypothetical (at least one hypothetical classification through KEGG or the nonredundant RefSeq database NR) or unknown (no database match), we observed high numbers of genes encoding proteins with hypothetical and unknown functions in all MAGs of *Ca*. Penumbrarchaeia (Table S14, Fig. S16). Considering the low relative abundance of *Ca*. Penumbrarchaeia MAGs in the screened metagenomic runs, this raises the question of whether the unexpectedly high number of genes encoding hypothetical and unknown proteins in this novel class may be related to its rarity. The percentage of genes encoding unknown proteins among the *Ca*. Penumbrarchaeia ranged between 1.6 and 63% in single MAGs with an average of 26%, and high percentages of genes encoding unknown proteins were found in MAGs of the families 2, 3 A, 3B, and 4 (43–63%), most of which derived from the water column (Fig. S16). Additionally, AGNOSTOS was run to investigate novelty at protein domain level [42]. Based on the AGNOSTOS classification, 14–29% of the encoded proteins in the *Ca*. Penumbrarchaeia MAGs were classified as genomic unknowns (genes with unknown function, derived from sequenced or draft genomes), and between 0.24 and 17% genes have been characterized as environmental unknowns (genes with unknown function, found only in environmental metagenomes or MAGs) (Fig. S17) with no further functional assignment. While classification through AGNOSTOS showed a higher percentage of genes encoding proteins with known protein domains, KEGG and NR annotations could not give functional assignments for these genes, which were therefore regarded as genes with novel function. Novel genes in this study were defined as those which are orphaned in function, despite possibly being found in other microorganisms.

**Fig. 6:**
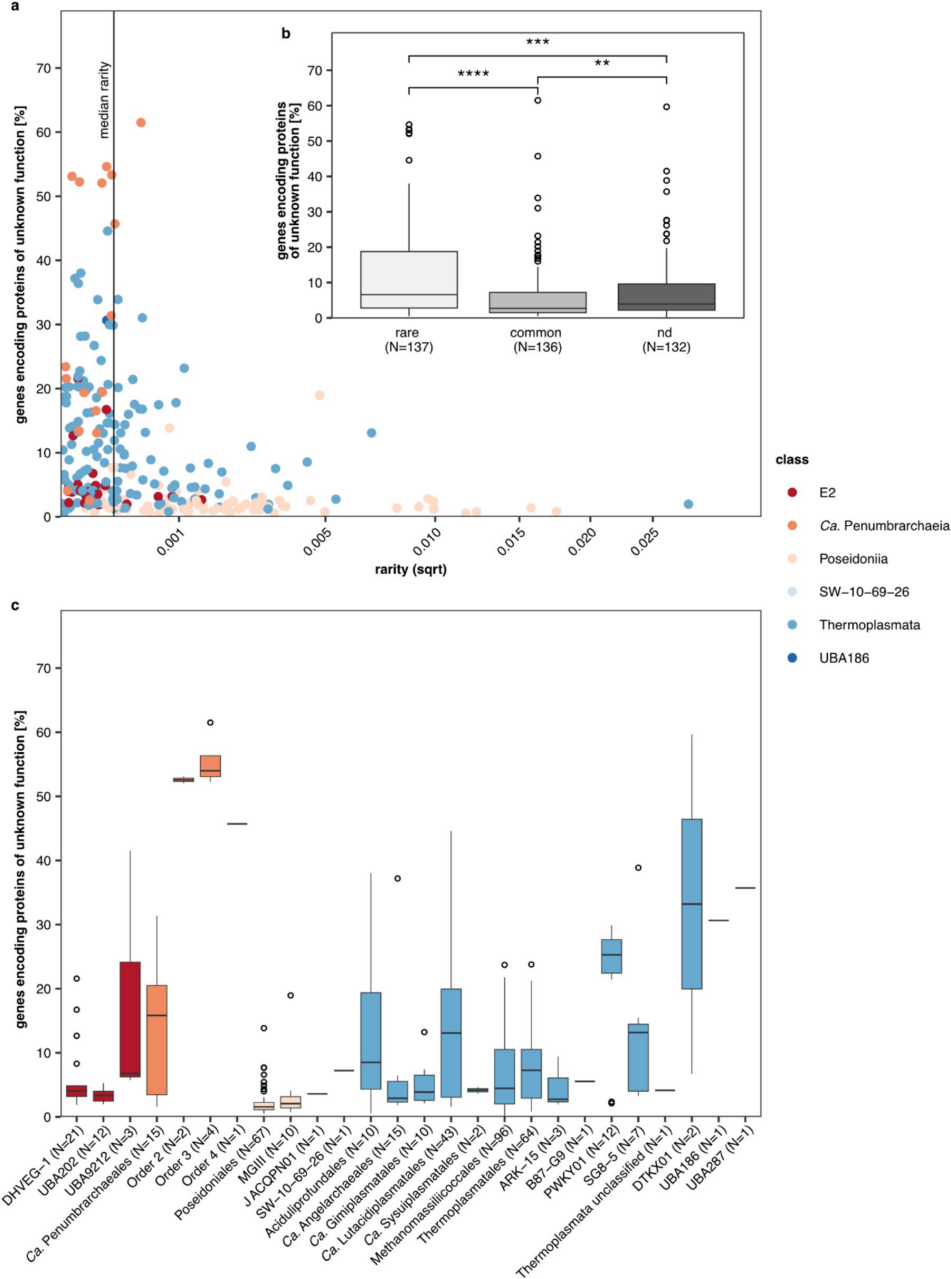
Relationship between protein novelty and MAG occurrence. **a** Percentage of genes encoding proteins of unknown function vs. rarity for each of the redundant MAGs within the phylum Thermoplasmatota. The *x*-axis was square-root (sqrt)-transformed. The rarity index was defined as median relative abundance weighted by the fraction of occurrence. MAGs below the median rarity were defined as rare, and MAGs above were defined as common. **b** Percentage of genes encoding proteins of unknown function in genomes of the three defined rarity groups: rare, common, and not detected (nd), which contain those genomes, to which none of the screened metagenomic short-read data mapped. Differences between groups were tested by Wilcoxon rank-sum tests, Bonferroni-adjusted significance threshold is 0.0167, and *p*-values are indicated by asterisks (****p < = 0.0001, ***p < = 0.001, **p < = 0.01). Number of observations *N* indicates number of genomes sorted into each of the defined rarity groups. **c** Percentage of genes encoding proteins of unknown function for each order found within the phylum Thermoplasmatota. Number of observations *N* represents the number of Thermoplasmatota genomes per order

To test for a correlation between the percentage of genes encoding unknown proteins and the occurrence of the MAGs within the phylum of Thermoplasmatota, we defined a rarity index as the median relative abundance of the MAG in the environment, weighted by the fraction of datasets in which the MAG occurred across all screened datasets (fraction of occurrence). Based on the rarity index, we differentiated between rare (rarity < median rarity) and common (rarity > median rarity) MAGs. The percentages of genes encoding unknown proteins in the rare group were higher than in the common group (Fig. 6b). Notably, we found that most of our *Ca*. Penumbrarchaeia MAGs (73%) were defined as rare by our definition (Fig. 6a, Table S16). Further, we observed that the three novel *Ca*. Penumbrarchaeia orders (orders 2, 3, and 4) hold a higher percentage of genes encoding unknown proteins compared to any other Thermoplasmatota order (Fig. 6c). MAGs in the category “not detected” were not found in any of the screened metagenomic runs derived from aquatic samples (Fig. 6) as these MAGs originated from soil, biodigesters, or human- and animal-associated habitats (Table S15). Thus, low or missing relative abundances for these nonaquatic Thermoplasmatota do not necessarily classify these taxa as overall rare but as rare in the screened environments.

Following up on the hypothesis of the rare biosphere acting as a gene pool for the community, we investigated if orthogroups found in the *Ca*. Penumbrarchaeia MAGs were also present in other Thermoplasmatota. While we observed significant differences between orthogroups shared among *Ca*. Penumbrarchaeia MAGs and those shared with other Thermoplasmatota for orthogroups in all three categories (annotated, hypothetical, and unknown; Wilcoxon rank-sum test, Bonferroni adjusted *p*-value: 0.0167), the absolute effect size was higher for hypothetical and unknown orthogroups (Impact Effect size test, annotated: 0.3496, hypothetical: 1.1955, unknown: 2.2668). Thus, hypothetical and unknown orthogroups found in the *Ca*. Penumbrarchaeia MAGs were mostly confined to the class *Ca*. Penumbrarchaeia (Fig. S18), while annotated orthogroups were more likely to be shared between *Ca*. Penumbrarchaeia MAGs and other Thermoplasmatota. Ancestral gene family reconstruction further predicted that the majority of these hypothetical and unknown orthogroups were gained by the four orders within the class *Ca*. Penumbrarchaeia, rather than being present in the last common ancestor and subsequently lost by other members of the Thermoplasmatota (Fig. S19).

## Discussion

The importance of the rare biosphere in the environment and its role in promoting community stability has been recognized only recently [2, 6, 13, 58, 59]. Studies on global phylogenetic diversity identified not only abundant taxa but also members of the rare biosphere [37–39, 60]. Yet, studies on the rare biosphere in marine environments are very limited, and the roles rare taxa play in these ecosystems remain elusive. We detected the novel and rare class *Ca*. Penumbrarchaeia within the phylum Thermoplasmatota in protein-amended enrichments. Using these first findings, we combined information gathered from the enrichment experiments and coupled these to extensive group-targeted data mining to investigate organic matter degradation by members of the *Ca*. Penumbrarchaeia. This class has been so far overlooked; it remains unexplored and scarcely represented in INSDC databases.

In this study, we identified the class *Ca*. Penumbrarchaeia as an organic matter degrader in sediments from the Helgoland mud area, likely able to survive on protein compounds as a sole carbon source, enabled by the presence of genes encoding pathways for the degradation of amino acids in all 35 *Ca*. Penumbrarchaeia MAGs (Fig. 5). The presence of a partial rTCA could be indicating an alternative pathway to convert the intermediate glutamate into acetyl-CoA as has been described previously by Yin et al. [61]. These results are consistent with findings from several other members of the phylum Thermoplasmatota, which live heterotrophically or mixotrophically by degrading organic matter, i.e., utilizing fatty acids, carbohydrates, proteins, peptides, and amino acids as their substrates in aquatic environments [44, 45, 61, 62].

Along with genes for the degradation of amino acids, MAGs of sediment-inhabiting *Ca*. Penumbrarchaeia families encoded genes for extracellular peptidases involved in protein polymer hydrolysis. A previous study identified low-abundant archaeal groups involved in catabolic protein degradation, utilizing proteins as both energy and carbon sources [61]. Generally, many microbial groups are capable of protein degradation, but in an anabolic fashion, incorporating extracellular peptides or amino acids as the carbon and nitrogen sources. In contrast, catabolic protein degraders appear to be rare in environments, as revealed by several studies using stable isotope probing approaches [61, 63, 64]. For example, some unknown Thermoplasmatota and Lokiarchaeia have been identified as dissimilatory protein degraders, yet they remain nearly undetectable in situ based on 16S rRNA gene sequencing [61]. An explanation is that in addition to the competition with widespread anabolic protein degraders, the bioavailability of free proteins or amino acids in environments is extremely limited (typically below 10 µM for amino acids) [65]. Such scarcity of accessible proteins as both energy and carbon sources will constrain the survival and prevalence of the catabolic degraders, which require a higher protein supply than the anabolic counterparts.

In MAGs derived from the water column, genes for protein degradation were not found, contrasting with other abundant planktonic Thermoplasmatota that were shown to degrade proteins [62]. The pelagic *Ca*. Penumbrarchaeia MAGs in our study might have lost the trait for protein degradation or thrive in close proximity to those microbes that host extracellular peptidases. Despite that missing genes in some families could also result from the incompleteness of the analyzed MAGs, our observation suggests different lifestyle preferences among the *Ca*. Penumbrarchaeia families.

Although the potential for fatty acid degradation was observed in some of the MAGs, the oxidation reaction is thermodynamically unfavorable, and mechanisms for the consumption of reducing equivalents are required [66]. As we did not observe an alternative electron acceptor, electrons might undergo bifurcation via the detected heterodisulfide reductase [67], which was present in some *Ca*. Penumbrarchaeia members with the potential for fatty acid oxidation. Alternatively, the fermentation of fatty acids could also become favorable by a low hydrogen partial pressure in the environment [66]. Therefore, despite showing the potential for fatty-acid degradation, the feasibility of this metabolism remains so far unresolved for members of *Ca*. Penumbrarchaeia.

Overall, all datasets containing reads of *Ca*. Penumbrarchaeia MAGs were confined to continental shelf environments. These areas are regarded as organic carbon storage hotspots where large amounts of carbon are supplied through river discharge and land runoff and large phytoplankton blooms in upwelling areas [68, 69]. Due to high organic matter input and high sedimentation rates, oxygen in such sediments can be depleted within the first millimeters, generating an anoxic environment [70]. Corroborating *Ca*. Penumbrarchaeia presence in anoxic environments, all MAGs from the water column were predominantly found in oxygen-minimum and oxygen-deficient zones, in which high bioavailability of marine organic matter is prevalent due to high primary productivity in the overlying water column and high respiration causes local anoxia [71–74]. The presence of all MAGs only in such organic, carbon-rich, and anoxic environments with high input of marine organic matter agrees with our findings that the class *Ca*. Penumbrarchaeia is involved in amino acid and partially protein degradation, besides being protected against possible oxidative stress.

Additional to the core metabolism shared among the majority of *Ca*. Penumbrarchaeia MAGs, we identified environment-specific adaptations. MAGs retrieved in this study were found in samples from marine sediments, lake sediments, hydrothermal sediments and vents, cold seeps, and the marine water column. While most *Ca*. Penumbrarchaeia families were found at distinct sites only and may therefore occupy very distinctive habitats, two families (1 A and 3 A) exhibited a more widespread distribution. We detected a high functional repertoire of the most heterogeneous family 1 A, reflected by a high number of family-specific orthogroups. Finding the highest number of MAGs in this family reflects its broad distribution. Specific environmental adaptations could be detected in most of the families. For example, families 1 A, 2, and 4 were found in locations of the Baltic Sea and the Cariaco Basin in Venezuela, which are in proximity to riverine input. As such, these locations might be experiencing input of waste water, which could explain the defensive MATE family drug/sodium antiporter excreting antimicrobial drugs or naturally occurring antibiotics in these MAGs. The presence of such genes might indicate antimicrobial resistance in these human-influenced environments [75–77]. Further, MAGs found in the Baltic Sea carried genes against arsenic toxicity, which is in agreement with high arsenic concentrations reported in this environment [78].

Not only the class *Ca*. Penumbrarchaeia did exhibit a habitat selection of specific continental shelf environments but also its relative abundance in the environment did not exceed 0.32%, with an average relative abundance of < 0.05%. In contrast to our findings, other classes within the Thermoplasmatota, such as *Ca*. Poseidonia and Thermoplasmata were found to have much higher relative abundances in the screened aquatic environments. Only recently, novel taxa, namely *Ca*. Sysuiplasmatales, *Ca*. Lunaplasmatales, *Ca*. Yaplasmales, and *Ca*. Gimiplasmatales, were described and are characterized as having a limited environmental distribution and low abundances [51, 79–81]. The continuous detection of novel rare taxa indicates the untapped diversity, which lies within the Thermoplasmatota and likely lies within other prokaryotic phyla as well. With continuous advances in sequencing technologies, deeper sequencing will shed more light on unknown microorganisms. Our findings highlight the importance of studying the rare biosphere to understand not only their metabolic functions, including so far undescribed functional diversity, but also evolutionary processes [34], such as the transition from an aerobic to anaerobic lifestyle among members of the Thermoplasmatota [51].

Besides being classified as a rare biosphere member, all orders of the class *Ca*. Penumbrarchaeia contained high percentages of genes encoding unknown and hypothetical proteins. It is a common feature of uncultivated and rare taxa to contain high numbers of unknown protein families [2, 40] with up to 70% of genes that cannot be annotated by databases such as KEGG and NR [38, 82], as these databases use complete genomes and functionally characterized genes as reference [83, 84]. These high numbers of unknown protein families may be indicative of the class functioning as a genetic seed bank under changing environmental conditions [2, 6, 7]. Especially, rare organisms are expected to harbor genes that could become important once environmental pressures arise. By activating these genes, community resilience is promoted.

To further investigate the distribution and origin of the genes encoding hypothetical and unknown proteins found within the *Ca*. Penumbrarchaeia MAGs in our study, we analyzed the presence of the respective genes in members of all Thermoplasmatota. Most of these genes were unique to the class *Ca*. Penumbrarchaeia, not being present in the last common ancestor to other Thermoplasmatota lineages and therefore most likely not inherited vertically. Some genes encoding unknown and hypothetical proteins might be acquired laterally, as they were predicted to have been gained independently by Thermoplasmatota orders outside the class *Ca*. Penumbrarchaeia. Still, the origin of most genes encoding unknown and hypothetical proteins found in the *Ca*. Penumbrarchaeia remains unknown. As our exploration of the potential for lateral gene transfer is limited to the phylum Thermoplasmatota, additional insights on the gene origin may be gained by extending the analysis to all prokaryotic lineages.

The low probability of lateral gene transfer for these uncharacterized genes raises the question of which evolutionary pressures and vectors of lateral gene transfer might be inactive. As members of the rare biosphere, the class *Ca*. Penumbrarchaeia could undergo less phage predation, and as such, phage-mediated gene transfer might be restricted; yet, also genetic distance to other organisms could be indicative of a lack of lateral gene transfer by transduction [85]. Moreover, cell density in the marine subsurface or marine water column could be too low to allow efficient gene transfer [86, 87]. The lack of gene transfer of these genes encoding uncharacterized proteins furthermore indicates that these genes rather act as accessory than core metabolic functions [88] and, as such, promote the potential of members of the class *Ca*. Penumbrarchaeia as genetic seed bank.

## Conclusions

With our study, we demonstrated how to combine targeted enrichments with metagenomic sequencing and group-targeted data mining to investigate the metabolic potential of the rare biosphere, specifically focusing on the uncharacterized class *Ca*. Penumbrarchaeia within the phylum Thermoplasmatota. We identified the class *Ca*. Penumbrarchaeia as utilizing proteins and amino acids in organic matter-rich and aquatic habitats with limited or no oxygen. Our study revealed habitat specificity for all families in this class with low abundances in their environments. We used the class *Ca*. Penumbrarchaeia to better understand features of rare microorganisms in the environment and could identify high percentages of genes encoding hypothetical and unknown proteins, compared to other classes of the phylum Thermoplasmatota. The limited lateral transfer of these uncharacterized genes offers an intriguing incentive to further explore the functional and genetic diversity among members of the rare biosphere. Temporally, variable abundances and niche preferences of rare biosphere members can be further investigated by sequencing so far underrepresented environments and over time series. Our findings not only shed light on the metabolic potential and habitat specificity of the class *Ca*. Penumbrarchaeia but also underscore the broader importance of exploring the rare biosphere. Building on this foundation, the data available in public archives present a valuable opportunity to target the generation of new data in areas of underrepresentation, such as the rare biosphere, by first thoroughly reusing what is available. With this study, we highlight the need for targeted and data-integrative approaches to gain further insights into the rare biosphere and unravel functions and metabolic potential that lie within these understudied taxa.

## Methods

### 1. Sample collection and enrichments

Sediment was collected from the Helgoland mud area (54°05′15.5″N, 7°58′05.5″E) by gravity coring in 2017 during the RV Heincke cruise HE483 [89]. Sediments from a depth of 45–70 cm were selected from gravity core HE483/2–2 for initial slurry incubations. We selected sediments from the Helgoland mud area for these incubations as it is renowned for its high organic content [90, 91]. Anoxic slurry incubations were set up using sediment and sterilized artificial seawater (ASW; composition 26.4-g NaCl, 11.2-g MgCl_2_ · 6H_2_O, 1.5-g CaCl_2_ · 2H_2_O, 0.7-g KCl, and 4.26-g Na_2_SO_4_ per liter) at a ratio of 1:4 (w/v). A total volume of 50-ml mixed slurry was dispensed in 120-ml serum bottles sealed with butyl rubber stoppers. To remove residual oxygen, the headspace of the serum bottles was exchanged with N_2_. The slurry was preincubated for 2 days. Four replicates were set up for each initial treatment, containing either 1.86 g/l egg white protein or no additional substrate as control. Two of the four treatments were additionally amended with an antibiotic mix (D-cycloserine, kanamycin, vancomycin, ampicillin, and streptomycin; 40 mg/l for each). Samples were incubated at 20 °C. After 25 days, one replicate of the protein enrichment with antibiotics and one replicate without antibiotics were additionally amended with sodium 2-bromoethanesulfonate (BES; 4 mM) for the inhibition of methanogenesis.

### 2. Further enrichment of target microorganisms

To retrieve highly enriched Thermoplasmatota from the first generation of enrichments, 5-ml slurry were anoxically transferred into Widdel medium after 372 days of the initial incubations. A total volume of 25-ml medium was anaerobically dispensed into 50-ml serum bottles, and the headspace was flushed with N_2_:CO_2_ (80:20, v/v) (BIOGON C20 E941/E290; Linde, Germany). As basal medium, anaerobic Widdel medium [92] was prepared using salt water medium (20.0-g NaCl, 3.0-g MgCl_2_ · 6-H_2_O, 0.2-g KH_2_PO_4_, 0.25-g NH_4_Cl, 0.5-g KCl, 0.15-g CaCl_2_ · 2-H_2_O per liter): After sterilization, the basal medium was cooled down under an N_2_:CO_2_ (80:20, v/v) atmosphere. Sterilized 1-M NaHCO_3_ (30 ml), trace element solution SL 10 (1 ml) [93], selenite-tungstate solution (1 ml) [92], seven vitamins solution (0.5 ml) [94], and sodium sulfide (Na_2_S · 9 H_2_O; final concentration 0.7 M) were added to 1-l basal medium. As a redox indicator, sterilized resazurin (0.2 ml; 0.2% w/v) was added. The pH was adjusted from 7.2 to 7.4.

All samples were amended once at the beginning of the enrichment experiment with 30-mM sulfate (Na_2_SO_4_) and an antibiotic mix (D-cycloserin, kanamycin, vancomycin, ampicillin, and streptomycin; 50 mg/l for each) that has been used previously to inhibit general bacterial activity in marine sediments [61, 95, 96]. Control samples were incubated without an additional carbon source. Protein samples were amended with 2.33 g/l egg white protein as the sole carbon source. Samples were incubated at 20 °C.

### 3. Nucleic acid extraction

Two milliliters of slurry was sampled anaerobically from each of the treatments at days 98, 157, and 372 of the second-generation enrichment (“Further enrichment of target microorganisms” section) for nucleic acids extraction according to Aromokeye et al. [97]. DNA pellets were washed twice with 1-ml cold 70% ethanol and eluted in 100-µl diethylpyrocarbonate (DEPC) treated water (Carl Roth, Germany). The quality of nucleic acids was checked with a NanoDrop 1000 spectrophotometer (PEQLAB Biotechnologie, Erlangen, Germany).

### 4. 16S rRNA gene amplicon sequencing

Illumina amplicon sequencing libraries were prepared as described in Aromokeye et al. [97] using 30 PCR cycles. Primers targeting the V4 region of the archaeal 16S rRNA were Arc519 F (5′-CAGCMGCCGCGGTAA-3′) [98] and Arc806R (5′-GGACTACVSGGGTATCTAAT-3′) [99]. Thermal cycling conditions were as follows: initial denaturation at 95 °C for 3 min, 30 cycles of denaturation at 95 °C for 20 s, annealing at 60 °C for 15 s, elongation at 72 °C for 15 s, and final elongation at 72 °C for 1 min. Amplicons were generated at Novogene (Cambridge, UK) on the NovaSeq 6000 platform (2 × 250 bp, Illumina) in mixed orientation by ligation, therefore resulting in forward and reverse amplicon orientation in both forward (R1) and reverse read files (R2). Reads were demultiplexed and primer clipped using cutadapt v 2.1. [100] and further processed using the package dada2 v1.16.0 [101] in R v4.0.2 [102]. R1 and R2 reads were trimmed to 150 and 160 bp with a maximum error rate of 2. Subsequently, error rates were learned and samples dereplicated and denoised independently for each library orientation, by pooling the data from all samples, using a modified loess function adapted for libraries with binned quality scores [103]. Error-corrected R1 and R2 reads were merged into amplicon sequence variants (ASVs), and sequence tables for forward and reverse orientations were combined by reorientation of the reverse ASVs. Chimeras, ASVs of unexpected lengths (< 249 bp and > 255 bp), and singletons were removed. A bootstrap cutoff of 80 was used to perform taxonomic classification with the assignTaxonomy function of dada2 with a newly formatted GTDB r214 reference database containing the full 16S rRNA gene set. For further processing of archaeal ASVs, all non-archaeal taxa were removed.

### 5. Quantitative PCR (qPCR)

We quantified 16S rRNA gene copy numbers of Archaea, Bacteria, and uncultured Thermoplasmatota in the second-generation enrichments. Reaction mixtures contained 1 × Takyon MasterMix (Eurogentec, Seraing, Belgium), 4-µg bovine serum albumin (Roche, Mannheim, Germany), 300-nM primers, 1 ng of DNA template, or 2 µl of standard in a total reaction volume of 20 µl. The primer pair used for archaea was 806 F/912R (5′-GACTACHVGGGTATCTAATCC-3′/5′-GTGCTCCCCCGCCAATTCCTTTA-3′, annealing temperature 58 °C) [104], for bacteria 8 Fmod/338Rmod (5′-AGAGTTTGATYMTGGCTCAG-3′/5′-GCWGCCWCCCGTAGGWGT-3′, annealing temperature 58 °C) [105, 106], for uncultured Thermoplasmatota (EX4484-6, now *Ca.* Penumbrarchaeia) the newly designed pair 472 F/633R (5′-CGGTAAATCTCTGGGTAAATCG-3′/5′-ACCCGTTCTGGTCGGACGCYTT-3′, annealing temperature 64 °C), and for Lokiarchaeia subgroup 2b a newly designed pair Loki2b_34 F/Loki2b_278R (5′-TCCGACTGCTATCCGGGTAA-3′/5′-TCACGGCCCTTATCGATCAT-3′, annealing temperature 60 °C). Thermal cycling conditions were as follows: initial denaturation at 95 °C for 5 min, 40 cycles of denaturation at 95 °C for 30 s, annealing at given temperatures for 30 s, and elongation at 72 °C for 40 s.

### 6. Metagenomic analysis

After analyzing the amplicon sequencing data, the protein and antibiotics amended replicate of day 157 (second generation), which showed a high proportion of Thermoplasmatota, was chosen for metagenomic sequencing. An amount of ~ 1-µg extracted DNA was used for metagenomic sequencing on the Illumina HiSeq 4000 platform (2 × 150 bp) at Novogene (Cambridge, UK). Metagenomic reads were adapter and quality trimmed using bbduk from bbmap version 38.86 [107]. De novo assemblies were generated with SPAdes v.3.15.5 using the flag *meta* [108] and megahit v1.2.9 using the preset *meta-sensitive* [109]. For read mapping, fasta headers of each of the assemblies were simplified with anvio-7.1 [110]; contigs < 1000 bp were removed, and, subsequently, the quality-trimmed reads were mapped back to the assemblies using bowtie2 v2.3.5.1 [111]. For each assembly, binning was performed with metabat2 v2.12.1 [112] and CONCOCT v1.0.0 [113], followed by bin refinement using the bin refinement module of metaWRAP v1.3.1 [114]. The refined bins of both assemblies were then dereplicated by dRep v3.0.0 using the ANImf algorithm with a primary ANI of 0.9 (mash v2.3 [115]) and a secondary ANI of 0.95 (fastANI v1.32 [116]) with a minimum aligned fraction of 0.5 [117]. Dereplicated bins were reassembled using the bin reassembly module of metaWRAP.

Quality of obtained MAGs was calculated with checkM2 v0.1.3 [118], and taxonomic classification was assigned through gtdbtk v2.1.0 [119], based on the GTDB database v207 [37].

### 7. Retrieval of EX4484-6 MAGs from public archives

To increase the number of EX4484-6 MAGs for further analysis, a list of in total 19,931 genome assemblies, consisting of all Thermoplasmatota as well as ecological and unclassified MAGs according to their NCBI taxon ID, was retrieved through the ENA advanced search interface (date accessed: 22.08.2022; Fig. S20). Genome assemblies were filtered based on their scientific names, removing those environments affiliated with anthropogenic activities. The remaining 11,479 MAGs were downloaded from RefSeq [84] or GenBank [120], quality assessed using checkM2 v0.1.3 [118], and filtered based on completeness (> 80%) and contamination (< 5%). Quality-filtered MAGs (1602) were taxonomically classified using gtdbtk v2.1.0 [119]. Only 714 MAGs classified as Thermoplasmatota were kept for further analyses. Additionally, this MAG collection was supplemented by 132 quality-filtered Thermoplasmatota MAGs from GTDB [37], which were not yet included in the ENA search output. Furthermore, 34 additional Thermoplasmatota MAGs from previous studies of *Ca*. Lutacidiplasmatales [50], *Ca.* Gimiplasmatales [80], *Ca*. Sysuiplasmatales [51], and uncultured Thermoplasmatota [61] were included in the dataset. The total remaining set of 844 MAGs was dereplicated using dRep v3.0.0 as described above (“Metagenomic analysis” section), resulting in a total number of 388 MAGs. Within this data set, five new nonredundant MAG clusters of EX4484-6 were found, of which four clusters were represented by one single MAG, while one cluster was represented by four MAGs, all of which derived from Guaymas Basin hydrothermal sediments [48, 121, 122]. The cluster representatives of four clusters were of marine origin and further used as input for the following mining of metagenomic short-read data for EX4484-6.

### 8. Mining of metagenomic short-read data from public archives

Data mining of environmental metagenomes was conducted to increase the number of MAGs for the group EX4484-6 by reanalysis of metagenomic short-read data (Supplementary Fig. 19). For this, a list containing raw reads of 44,968 ecological metagenomes was downloaded from the ENA advanced search (date accessed: 10.11.2022) using the parameters tax_tree (410,657), library strategy “WGS,” instrument platform “ILLUMINA,” library source “METAGENOMIC,” and library selection “RANDOM.” Samples without location information, unspecified instrument model, missing ftp links, and a base count below 3 Gbp were removed from the selection. Through manual filtering, nonaquatic samples were further removed, leaving a data set of 8287 metagenomes.

In a first screening step, all 8287 metagenomes were downloaded, quality trimmed using bbduk from bbmap version 38.86 [107], and mapped against the previously identified EX4484-6 clusters (MAG from enrichments, “Metagenomic analysis” section; dereplicated marine EX4484-6 clusters, “Retrieval of EX4484-6 MAGs from public archives” section) using bwa v0.7.17 [123]. The per-genome coverage was estimated from the number of mapped reads using coverm v0.6.1 [124] with a minimum read percent identity of 50%, a minimum aligned percentage of 50%, and a minimum read overlap of 30 bp between read and reference. Cumulative coverage was calculated for all datasets within a given study accession. Studies with a cumulative coverage for EX4484-6 MAGs > 2 were used for de novo co-assembly and binning, unless MAGs associated with the study had already been published. In these cases, published MAGs were screened for EX4484-6 MAGs along with the catalog of Earth’s microbiome [38], the OceanDNA MAG catalog [125], and the Ocean Microbiomics Database OMDB (v2) [39, 126]. MAGs from the OMDB (v2) were contributed by the co-authors and produced as previously published in Paoli et al. [39]. A more detailed pipeline description is included in supplementary methods. Further, high-quality EX4484-6 MAGs from unpublished work were contributed to this study by co-authors. Raw reads from studies without published MAGs were downloaded and adapter and quality trimmed using bbduk v38.86 [107]. For each study, de novo co-assemblies were computed using megahit v1.2.9 [109] with either the preset meta-large, starting at the lowest possible kmer size (27 or 37) for large sample sizes or a modified preset meta-sensitive starting at kmer size 23. Then, quality-trimmed reads were mapped onto the co-assembly using bowtie2 v2.3.5.1 [111] and binned, refined, and subsequently classified using gtdbtk v2.1.0 [119] with GTDB database v207 as previously described (“Metagenomic analysis” section). If bins of the class EX4484-6 were present, all Thermoplasmatota MAGs within the bin set were reassembled using the metaWRAP bin reassembly module followed again by taxonomic classification. All newly found EX4484-6 MAGs were additionally manually refined using anvio v7.1 [110].

### 9. Annotation of EX4484-6 MAGs

Genes of EX4484-6 MAGs were predicted using prodigal v2.6.3 [127]. Predicted genes were annotated with the nonredundant RefSeq database (NR) (accessed 23 June 2023) [128] and KEGG release 104 [83, 129] using diamond blastp v2.0.15 [130]. Additionally, we retrieved a full annotation of all MAGs using InterProScan v5.65–97.0 [131, 132]. Peptidases were identified by diamond blastp against the MEROPS database v12.4 [133]. From these, extracellular peptidases (signal peptides) were determined using signalp v6.0 [134]. Signal peptides were only counted as such if they were annotated as “SP” and contained an additional MEROPS annotation. CAZymes were annotated using dbCAN v3.0.7 [135]. To reduce false-positive CAZymes in the annotation, all predicted CAZymes were manually validated with the KEGG and NR annotations. Only those CAZymes with more than one annotation according to dbCAN that were also represented in KEGG or NR were counted as positive hits. Lastly, transporters were annotated with the Transporter Classification Database (accessed 12/2023) [136] using diamond blastp v2.0.15 [130] to improve information on transporters found through NR and KEGG. For all blastp searches, a blast score ratio [137] threshold of 0.4 was applied. If metabolic pathways were incomplete, also annotations below the blast score ratio were analyzed.

From all predicted genes for each of the redundant EX4484-6 MAGs, genes were sorted into the categories annotated, hypothetical and unknown based on their annotation status. Annotated genes were defined as genes which had a functional annotation from KEGG or NR, hypothetical genes were defined from the remaining genes without functional annotation if they had at least one hypothetical classification, and genes without any annotation were defined as functionally unknown genes. To further characterize the unknown genes in the EX4484-6 MAGs, we applied AGNOSTOS v1.1 [42] with default parameters.

To analyze clusters of orthologous genes within the class of EX4484-6, all predicted genes were clustered into orthogroups using OrthoFinder v2.5.5 with multiple sequence alignment for tree inference [53]. From all orthogroups, a nonmetric multidimensional scaling (NMDS) was computed using the R function metaMDS from the package vegan v2.6.4 [138] with Jaccard dissimilarities based on shared orthogroups between MAGs. For visualization of family-specific orthogroups, an upset plot was created using the package UpSetR v1.4.0 [139]. Orthogroups, which were present in at least 90% of all redundant EX4484-6 MAGs, were defined as the core genome, and annotations of genes therein were analyzed.

For contextualization of the proportions of unknown genes in the EX4484-6 MAGs, we similarly annotated all Thermoplasmatota genomes (“Retrieval of EX4484-6 MAGs from public archives” section) using NR and KEGG and sorted predicted genes into the three previously mentioned categories: annotated, hypothetical, and unknown. Additionally, orthogroups across all Thermoplasmatota were computed using the rooted phylogenomic tree (“Marker gene tree and phylogenomic analyses” section) as the input tree and sorted based on their annotation status as described previously. The proportion of EX4484-6 orthogroups shared within EX4484-6 MAGs and between Thermoplasmatota and EX4484-6 MAGs was calculated (“Retrieval of EX4484-6 MAGs from public archives”). Wilcoxon rank-sum tests were then applied to test for differences in that proportion between EX4484-6 and other Thermoplasmatota for each annotation status, and the effect size for each difference was computed using the package ImpactEffectsize in R [140]. The *p*-value was corrected for executing three pairwise comparisons using the Bonferroni correction.

### 10. Marker gene tree and phylogenomic analyses

All dereplicated Thermoplasmatota MAGs (“Retrieval of EX4484-6 MAGs from public archives” section) together with all redundant EX4484-6 MAGs (“Mining of metagenomic short-read data from public archives” section) were used as input for a phylogenomic marker gene tree. In total, the tree contained 419 genomes, including an outgroup consisting of 14 medium to good quality (completeness > 80%, contamination < 5%), randomly selected Halobacteriota genomes. A multiple sequence alignment (MSA) with 53 marker genes was generated using the de novo workflow of gtdbtk v2.1.0 [119]. With the resulting MSA, a suitable model (LG + I + R10 + F) was identified using ModelFinder [141] as implemented within iqtree2 v2.2.2.7 [142]. The marker gene tree was calculated using raxml-ng v1.1.0 [143] with 20 starting trees. Bootstrap convergence with a bootstrap cutoff at 0.02 was reached after 100 trees. The tree was rooted at the outgroup and manually collapsed based on the GTDB v207 taxonomy [37] using iTol v6.8.1 [144]. Further, relative evolutionary divergence (RED) was calculated using the function getreds from the R package castor [145]. Additionally, average nucleotide identity (ANI) was calculated using fastANI v1.32 [116], and amino acid identity (AAI) was computed using fastAAI v0.1.18 [146]. Based on this information, the class EX4484-6 was described as *Ca*. Penumbrarchaeia (Supplementary results and discussion II). In addition to the gene tree based on 53 archaeal marker genes, a maximum-likelihood species tree was computed from gene trees of all orthogroups that were part of the core genome (Annotation of EX4484-6 MAGs) using SpeciesRax implemented in GeneRax v.2.1.3 [147, 148].

For gene family reconstruction of uncharacterized genes found in the class *Ca*. Penumbrarchaeia, Wagner parsimony was computed with a gain penalty of 1 using COUNT [149] for the phylum Thermoplasmatota, using those orthogroups of the class *Ca*. Penumbrarchaeia that contained genes encoding hypothetical and unknown proteins.

### 11. Relative MAG abundance in the environment

All redundant *Ca*. Penumbrarchaeia MAGs were dereplicated using dRep v3.0.0 as described above (“Metagenomic analysis” section), resulting in 20 nonredundant MAGs. Among these clusters, three of the initial four marine cluster representatives (“Retrieval of EX4484-6 MAGs from public archives” section) were found. The fourth cluster was represented by a MAG from a different study, showing higher completeness and lower contamination than the previously obtained cluster representative while still deriving from the same sediment (Guaymas Basin). To quantify representatives of *Ca*. Penumbrarchaeia in the environment, a competitive mapping index was constructed from the dereplicated *Ca*. Penumbrarchaeia MAGs and other representatives of the Thermoplasmatota phylum (“Retrieval of EX4484-6 MAGs from public archives” section) using bowtie2 v2.3.5 [111]. For obtaining relative abundances of *Ca*. Penumbrarchaeia MAG representatives in the environment, the initial 8287 metagenomic runs (“Mining of metagenomic short-read data from public archives” section) were downloaded along with 286 additional TARA ocean runs, which we initially excluded from our metagenomic run screening as unlikely to contain sufficient EX4484-6-affiliated reads for MAG recovery (“Mining of metagenomic short-read data from public archives” section). PhiX sequences and adapters were removed, followed by quality trimming using a minimum read length equal to 2/3 of the initially generated read length or 100 bp for sequencing runs with more than 160 cycles, a trimming quality of 20, and a minimum average quality of 10 with bbduk from bbmap version 38.86 [107]. Quality-trimmed reads were aligned to the competitive mapping index using bowtie2. Mean coverage, breadth of coverage, and relative abundances of single MAGs were computed using coverm genome v0.6.1 [124] with a minimum breadth of coverage of 50% and a percentage identity of at least 95%.

### 12. Evaluation of rarity in the phylum Thermoplasmatota

Rarity for each nonredundant *Ca*. Penumbrarchaeia MAG was defined as the median of its relative abundance in the metagenomic runs, where it was detected (“Relative MAG abundance in the environment” section), weighted by the fraction of the runs in which it occurred in of all screened runs. The median rarity across all Thermoplasmatota MAGs was calculated and then defined as the cutoff between rare (rarity < median rarity) and common MAGs (rarity > median rarity) in our dataset. Additionally, a third group was contributed by those MAGs that were not detected in any of the screened metagenomic runs. The percentages of unknown genes (“Annotation of EX4484-6 MAGs” section) were sorted by rarity groups (common, rare, not detected), and Wilcoxon rank-sum tests were applied to test for differences between these three groups. The *p*-value was corrected for executing three pairwise comparisons using the Bonferroni correction.

## Supplementary Information

Supplementary Material 1: Figures **S1**. Microbial community composition of second-generation enrichments. Relative abundances of (a.) archaeal 16S rRNA genes, (b.) *Ca.* Penumbrarchaeia ASVs and (c.) Lokiarchaeia ASVs at day 98 and 157 in two replicates each of control samples and protein amended samples, both amended with 30 mM sulfate (Na_2_SO_4_) and an antibiotics mix (D-cycloserin, kanamycin, vancomycin, ampicillin and streptomycin; 50 mg/l for each). Protein samples were additionally amended with 2.33 g/l egg white protein. **S2**. Microbial community composition of second-generation enrichments. 16S rRNA gene copies per ml slurry of Bacteria, Archaea, the class *Ca.* Penumbrarchaeia and *Lokiarchaeia* subgroup Loki-2b. Gene copies are shown for both replicate enrichments (Replicate 1, Replicate 2*) of control samples and protein amended samples, both amended with 30 mM sulfate (Na_2_SO_4_) and an antibiotics mix (D-cycloserin, kanamycin, vancomycin, ampicillin and streptomycin; 50 mg/l for each). Protein samples were additionally amended with 2.33 g/l egg white protein. Error bars represent standard deviations of the three technical qPCR replicates per sample. **S3**. Phylogenetic reconstruction of the class *Ca*. Penumbrarchaeia. (a.) Maximum-likelihood tree (RAxML, convergence reached after 1300 bootstraps) of 354 full length 16S rRNA gene sequences of Thermoplasmatota, including all retrieved full length *Ca*. Penumbrarchaeia 16S rRNA genes from single MAGs. Shorter 16S rRNA gene sequences of ASVs and those found in *Ca.* Penumbrarchaeia MAGs were added to the existing tree. (b.) Maximum-likelihood tree based on orthogroups that were part of the core genome and detected in > 90% of all *Ca*. Penumbrarchaeia MAGs. The species tree was inferred from gene trees of all orthogroups using SpeciesRax [98]. **S4**. Partial phylogenomic tree of the Thermoplasmatota including RED. Maximum-likelihood tree (RAxML, 100 bootstraps) of 370 Thermoplasmatota MAGs and 35 *Ca*. Penumbrarchaeia MAGs obtained through data mining of genome assemblies and metagenomic short read data sets. Node labels display taxonomic affiliation on class level. The class *Ca.* Penumbrarchaeia clusters with Thermoplasmata_A/DTKX_01 according to the marker gene tree and is indicated by a red box (Fig. 3a). Node values indicate relative evolutionary divergence (RED). The complete tree is provided as separate PDF. **S5**. Average nucleotide identity (ANI). Heatmap of ANI between all 35 *Ca.* Penumbrarchaeia MAGs obtained during our data mining. Numbers in colored tiles indicate the ANI percentage between the compared MAGs (threshold ANI > 75%). MAGs were ordered according to their taxonomy in the marker gene tree (Fig. 3. **S6**. Amino acid identity (AAI). Heatmap of AAI between all 35 *Ca.* Penumbrarchaeia MAGs obtained during our data mining. Numbers in colored tiles indicate the AAI percentage between the compared MAGs. MAGs were ordered according to their taxonomy in the marker gene tree (Fig. 3). **S7**. Distribution of the class *Ca.* Penumbrarchaeia. World map showing the distribution, relative abundance and environment of all observed *Ca.* Penumbrarchaeia detected in 128 samples. Relative abundances were calculated by aligning quality trimmed reads of 8573 metagenomic sequencing runs to a competitive mapping index containing 20 non-redundant *Ca.* Penumbrarchaeia MAGs. Point colors indicate the environment, point sizes indicate relative abundance. **S8**. Abundance of non-redundant Thermoplasmatota in the environment. (a.) As fraction of occurrence for each order found within the non-redundant Thermoplasmatota data set. The fraction of occurrence was defined as fraction of data sets MAGs occurred in across all screened data sets. Number of observations N represents the number of Thermoplasmatota genomes per order. (b.) As relative abundance of each order in samples they occurred in. Number of observations N represents the number of metagenomic runs, in which the genome was detected. Order 1—Order 4 correspond to the *Ca*. Penumbrarchaeia orders, with Order 1 being *Ca.* Penumbrarchaeales. **S9**. Clustering of *Ca.* Penumbrarchaeia MAGs based on orthogroups. a NMDS based on all orthogroups found in all 35 *Ca.* Penumbrarchaeia. The NMDS was computed using the function metaMDS from the package vegan v2.6.4 with Jaccard dissimilarities and two dimensions. Single families are indicated by different colors. Family 1 A corresponds to *Ca.* Penumbrarchaeaceae. b Upset plot showing intersections of orthogroups for all six families. Intersections shown reflect 95% of all orthogroups within the data set. Numbers of orthogroups per intersection are indicated above the bars. Orthogroup intersections, which are only present in sediment or water are indicated by color. Total number of different orthogroups per family are indicated by horizontal bars next to the upset plot. **S10**. Shared orthogroups between *Ca.* Penumbrarchaeia MAGs. Heatmap of shared orthogroups between all 35 *Ca.* Penumbrarchaeia MAGs. Numbers in colored tiles in the heatmap indicate the percentage of shared orthogroups between the compared MAGs. MAGs were ordered according to their family taxonomy in the marker gene tree (Fig. 3). Family 1 A corresponds to *Ca.* Penumbrarchaeaceae. **S11**. Peptidases in *Ca.* Penumbrarchaeia MAGs. a Extracellular peptidase homologs within MAGs of the class *Ca*. Penumbrarchaeia. b Peptidase homologs within MAGs of the class *Ca.* Penumbrarchaeia. MAGs were ordered according to their family taxonomy in the marker gene tree (Fig. 3). Family 1 A corresponds to *Ca.* Penumbrarchaeaceae. **S12**. Metabolic reconstruction of *Ca*. Penumbrarchaeales (Order 1) (*Ca*. Penumbrarchaeaceae (Family 1 A), Family 1B). Pathways in grey represent pathways with missing genes that are therefore not functional. Gene abbreviations can be found in Supplementary Table 9. The presence of genes is indicated by full or half circles for each family. Red stars indicate the presence of genes in all families. **S13**. Metabolic reconstruction of Order 2 (Family 2). Pathways in grey represent pathways with missing genes that are therefore not functional. Gene abbreviations can be found in Supplementary Table 9. The presence of genes is indicated by full or half circles. **S14**. Metabolic reconstruction of Order 3 (Family 3 A, Family 3B). Pathways in grey represent pathways with missing genes that are therefore not functional. Gene abbreviations can be found in Supplementary Table 9. The presence of genes is indicated by full or half circles for each family. Red stars indicate the presence of genes in all families. **S15**. Metabolic reconstruction of Order 4 (Family 4). Pathways in grey represent pathways with missing genes and are therefore not functional. Gene abbreviations can be found in Supplementary Table 9. The presence of genes is indicated by full or half circles. **S16**. Annotation status of predicted genes in *Ca.* Penumbrarchaeia MAGs. Genes classified as annotated, hypothetical and unknown based on NR and KEGG annotations shown as (a.) number of genes and (b.) percentage of genes. MAGs were ordered according to their family taxonomy in the marker gene tree (Fig. 3). Family 1 A corresponds to *Ca.* Penumbrarchaeaceae. S17. AGNOSTOS annotation category of genes in *Ca.* Penumbrarchaeia MAGs. Gene classification based on AGNOSTOS. Gene classifications are shown as (a.) number of genes and (b.) percentage of genes. MAGs were ordered according to their family taxonomy in the marker gene tree (Fig. 3). Family 1 A corresponds to *Ca.* Penumbrarchaeaceae. **S18**. Shared orthogroups among Thermoplasmatota. Boxplot of Thermoplasmatota genomes sharing orthogroups (OGs) present within *Ca.* Penumbrarchaeia MAGs separated by annotation category: annotated, hypothetical and unknown, according to their NR and KEGG annotation. Differences between groups were tested by Wilcoxon Signed Rank test, *p*-values are indicated by asterisks (**** *p* < = 0.0001). The measure of impact describes the effect size. Number of observations N indicates the number of orthogroups in each category. Number of genomes per group is indicated by number n. **S19**. Ancestral gene family reconstruction of orthogroups containing genes with hypothetical or unknown function. Evolutionary events are indicated for each order within the phylum of Thermoplasmatota representing orthogroup gains, expansions and losses. Evolutionary events were inferred using the Wagner parsimony in COUNT (gain penalty = 1) [99]. **S20**. Flowchart of methods applied during the data mining conducted in this study. Retrieval of *Ca.* Penumbrarchaeia (EX4484-6) MAGs from public archives (methods Sect. 7) is displayed in green, the subsequent mining of metagenomic short read data from public archives is displayed in blue (methods Sect. 8). Annotation and classification (methods Sect. 9) was conducted on both, the MAGs retrieved from public archives and MAGs reconstructed from metagenomic short read data (yellow). Programs and settings used are indicated next to or below arrows.

Supplementary Material 2: Tables **S1**. ASV counts and relative abundances of archaeal 16S rRNA gene amplicon sequencing for control and protein amended samples. **S2**. Top hits of a megablast search of the *Ca*. Penumbrarchaeia ASV sq2 and 16S rRNA gene obtained from the *Ca*. Penumbrarchaeia MAG E3_1_d157 against the rRNA/ITS database of the NCBI RefSeq Targeted Loci Project. **S3**. Overview list of metagenomic studies, in which *Ca*. Penumbrarchaeia MAGs had a coverage > 2. These studies were used to reconstruct *Ca*. Penumbrarchaeia MAGs. **S4**. Overview list of MAGs obtained in this studyS4. Overview list of MAGs obtained in this study. **S5**. Quality statistics computed with checkM and checkM2 and taxonomy information computed using gtdb_tk with the GTDB database v207S5. Quality statistics computed with checkM and checkM2 and taxonomy information computed using gtdb_tk with the GTDB database v207. **S6**. Table containing relative abundances of single MAGs in mapped metagenomic runs. **S7**. List of all unique metagenomic runs, in which *Ca*. Penumbrarchaeia MAGs were found with meta data derived from ENA of the sampling location and environment details. . **S8**. List of Orthogroups, which were selected as core genome (Orthogroup present in at least 90% of MAGs). The table shows gene counts for each MAG within single orthogroups. **S9**. Gene counts for single pathways within each of the *Ca*. Penumbrarchaeia MAGs. Genes were extracted from the core genome and annotated using NR and kegg. Based on found annotations genes were grouped into kegg pathway descriptions. **S10**. Full annotation of genes, which were found in all orthogroups of the core genome. Annotation was performed using kegg, NR, OM_RGC, dbCAN and merops. **S11**. Full annotation of selected pathways for all 35 Ca. Penumbrarchaeia MAGs. Annotation was performed using KEGG (release 104) and NR (release 13/05/2023), dbCAN (v3.0.7) and merops (v12.4). **S12**. Annotation of peptidases found in all 35 *Ca*. Penumbrarchaeia MAGs. The annotation was performed using merops (v12.4). **S13**. Annotation of CAZymes found in all 35 *Ca*. Penumbrarchaeia MAGs. The annotation was performed using dbCAN (v3.0.7). Results were validated with NR and KEGG annotations.**S14**. Gene counts for the categories annotated, hypothetical and unknown, along with percentages for each category. **S15**. Counts of rarity groups per habitat. **S16**.Overview table of all Thermoplasmatota MAGs with their taxonomic classification, fraction of occurrence, percentage of unknown genes (perentage_unknown), rarity and habitat. **S17**. Studies from selected marine metagenomics literature and matched with BioProject identifiers from the European Nucleotide Archive for MAG reconstruction in OMDv2

Supplementary Material 3. Proposal of type material and higher ranks. Methods: I. Clone library construction. II. Standard preparation for qPCR. III. qPCR primer design. IV. 16S rRNA gene phylogenetic tree. V. Data collection, processing and MAG reconstruction in OMDB (v2). Results and discussion: I. Additional qPCR results. II. Delineation of the class *Ca*. Penumbrarchaeia. III. Annotation of the class *Ca.* Penumbrarchaeia. Carbon metabolism. Carbon assimilation. Hydrogenases and energy conservation. Transporters. Stress response.

## Data Availability

All code used to perform analyses in this study is available on zenodo https://doi.org/10.5281/zenodo.15464567. Amplicon and metagenomic raw reads for this study have been deposited in the European Nucleotide Archive (ENA) at EMBL-EBI under accession number PRJEB80318 (https://www.ebi.ac.uk/ena/data/view/PRJEB80318), using the data brokerage service of the German Federation for Biological Data (GFBio [150]), in compliance with the Minimal Information about any (X) Sequence (MIxS) standard [151]. Ca. Penumbrarchaeia MAGs obtained and generated in this study and all data tables used for figure generation are available on zenodo https://zenodo.org/records/10813815. The type species genome has been deposited in the European Nucleotide Archive (ENA) at EMBL-EBI under accession number GCA_965234725.1. Clone sequences were deposited at GenBank under the accession numbers PQ255994-PQ256084.
