# Supplementary material for: Extensive data mining uncovers novel diversity among members of the rare biosphere within the Thermoplasmatota
Source: Microbiome. 2025 Jul 1;13:155. doi: 10.1186/s40168-025-02140-8 (PMC12220078; doi:10.1186/s40168-025-02140-8)
Supplement: Supplementary file 2 — Supplementary Material 1: Figures S1. Microbial community composition of second-generation enrichments. Relative abundances of (a.) archaeal 16S rRNA genes, (b.) Ca. Penumbrarchaeia ASVs and (c.) Lokiarchaeia ASVs at day 98 and 157 in two replicates each of control samples and protein amended samples, both amended with 30 mM sulfate (Na2SO4) and an antibiotics mix (D-cycloserin, kanamycin, vancomycin, ampicillin and streptomycin; 50 mg/l for each). Protein samples were additionally amended with 2.33 g/l egg white protein. S2. Microbial community composition of second-generation enrichments. 16S rRNA gene copies per ml slurry of Bacteria, Archaea, the class Ca. Penumbrarchaeia and Lokiarchaeia subgroup Loki-2b. Gene copies are shown for both replicate enrichments (Replicate 1, Replicate 2*) of control samples and protein amended samples, both amended with 30 mM sulfate (Na2SO4) and an antibiotics mix (D-cycloserin, kanamycin, vancomycin, ampicillin and streptomycin; 50 mg/l for each). Protein samples were additionally amended with 2.33 g/l egg white protein. Error bars represent standard deviations of the three technical qPCR replicates per sample. S3. Phylogenetic reconstruction of the class Ca. Penumbrarchaeia. (a.) Maximum-likelihood tree (RAxML, convergence reached after 1300 bootstraps) of 354 full length 16S rRNA gene sequences of Thermoplasmatota, including all retrieved full length Ca. Penumbrarchaeia 16S rRNA genes from single MAGs. Shorter 16S rRNA gene sequences of ASVs and those found in Ca. Penumbrarchaeia MAGs were added to the existing tree. (b.) Maximum-likelihood tree based on orthogroups that were part of the core genome and detected in > 90% of all Ca. Penumbrarchaeia MAGs. The species tree was inferred from gene trees of all orthogroups using SpeciesRax [98]. S4. Partial phylogenomic tree of the Thermoplasmatota including RED. Maximum-likelihood tree (RAxML, 100 bootstraps) of 370 Thermoplasmatota MAGs and 35 Ca. Penumbrarchaeia MAGs obtained thro [file 40168_2025_2140_MOESM1_ESM.docx]

**Extensive data mining uncovers novel diversity among members of the rare biosphere within the Thermoplasmatota**

Mara D. Maeke^1^, Xiuran Yin^1,2*^, Lea C. Wunder^1^, Chiara Vanni^3^, Tim Richter-Heitmann^1^, Samuel Miravet-Verde^4^, Hans-Joachim Ruscheweyh^4^, Shinichi Sunagawa^4^, Jenny Fabian^5^, Judith Piontek^5^, Michael W. Friedrich^1,3*^ & Christiane Hassenrück^5*^

^1^Microbial Ecophysiology Group, Faculty of Biology/Chemistry, University of Bremen, Bremen, Germany

^2^State Key Laboratory of Marine Resource Utilization in South China Sea, Hainan University, Haikou, China

^3^MARUM – Center for Marine Environmental Sciences, University of Bremen, Bremen, Germany

^4^Department of Biology, Institute of Microbiology and Swiss Institute of Bioinformatics, ETH Zurich, Zurich, Switzerland

^5^Leibniz Institute for Baltic Sea Research Warnemünde (IOW), Rostock, Germany

*Correspondence:

Prof. Dr. Michael W. Friedrich

University of Bremen

Microbial Ecophysiology Group

James-Watt-Straße 1 28359 Bremen

Germany

Phone: +49 (0) 421 218-63060

Email: michael.friedrich@uni-bremen.de

Dr. Christiane Hassenrück

Leibniz Institute for Baltic Sea Research Warnemünde (IOW)

Biological Oceanography

Seestrasse 15 18119 Rostock-Warnemünde

Germany

Phone: +49 (0)381 5197 227

Email: christiane.hassenrueck@io-warnemuende.de

Dr. Xiuran Yin

State Key Laboratory of Marine Resource Utilization in South China Sea

Hainan University

Renmin Ave. No.58 570228 Haikou

China

Email: 996383@hainanu.edu.cn

**Content**

Proposal of type material and higher ranks

Supplementary Methods

Supplementary Results

Supplementary Figures

Supplementary Tables (excel file)

Supplementary References

**Proposal of type material and higher ranks**

We propose one type species based on one of the 35 MAGs used in this present study. *Candidatus* Penumbrarchaeum helgolandense (GCA_965234725.1) representing the class, formerly known as “EX4484-6”. Based on this type species, we further propose the genus *Candidatus* Penumbrarchaeum gen. nov., *Candidatus* Penumbrarchaeaceae fam. nov., *Candidatus* Penumbrarchaeales ord. nov. and *Candidatus* Penumbrarchaeia class nov. The taxon names were registered in SeqCode (seqco.de/r:zcce2eqc) [1].

**Description of ‘*Candidatus* Penumbrarchaeum’ gen. nov.**

*Candidatus* Penumbrarchaeum (Pe.num.brar.chae'um. N.L. fem. n. *penumbra* the partial shadow as in partially hidden; presence in the rare biosphere, at the edge of detection; N.L. neut. n. *archaeum,* ancient one, archaeon; N.L. neut. n. *Penumbrarchaeum* the archaeon hidden in the biosphere at the edge of detection). Inferred to be of marine sediment origin, an anaerobe fermenting proteins, peptides and amino acids. Members were of low abundance (< 0.32%) and thus classified as rare biosphere. Type species: *Candidatus* Penumbrarchaeum helgolandense.

**Description of ‘*Candidatus* Penumbrarchaeum helgolandense’ sp. nov.**

*Candidatus* Penumbrarchaeum helgolandense (N.L. neut. adj. *helgolandense* of Helgoland, the region where the Helgoland mud area is located in the North Sea, and this organism originated from), named to indicate the origin of the species. Description is the same as for *Candidatus* Penumbrarchaeum gen. nov. represented by the MAG (metagenome-assembled genome) E3_1_d157 (3.46 Mbp), obtained from metagenomic sequences retrieved from an enrichment of Helgoland mud area sediment (54°05’15.5’’N, 7°58’05.5’’E) from a sediment depth of 45-70cm. Based on the genome reporting standards for MAGs [2], *Candidatus* Penumbrarchaeum helgolandense is defined as “high-quality” draft MAG with an estimated completeness of 97.86%, contamination of 2.81%, the presence of 5S (102bp), 16S (1466bp) and 23S (2880bp) rRNA genes and 40 tRNAs encoding 18 amino acids.

**Description of ‘*Candidatus* Penumbrarchaeaceae’ fam. nov.**

*Candidatus* Penumbrarchaeaceae (Pe.num.brar.chae.a'ce.ae. N.L. fem. pl. n. *Penumbrarchaeaceae*, the *Penumbrarchaeum* family). The description is the same as for *Candidatus* Penumbrarchaeum gen. nov. with the following additions: -aceae ending to denote a family. Candidatus Penumbrarchaeaceae was previously known as the family “EX4484-6”. Members of the family were inferred to be found in sediments of marine and freshwater origin as well as cold seeps. Type genus: *Candidatus* Penumbrarchaeum gen. nov.

**Description of ‘*Candidatus* Penumbrarchaeales’ ord. nov.**

Candidatus Penumbrarchaeales (Pe.num.brar.chae.a'les. N.L. fem. pl. n. *Penumbrarchaeales*, the *Penumbrarchaeum* order). The description is the same as for *Candidatus* Penumbrarchaeum gen. nov. with the following additions: -ales ending to denote an order. *Candidatus* Penumbrarchaeales ord. nov. was previously known as “EX4484-6” and comprises two families *Candidatus* Penumbrarchaeaceae fam. nov. and Family 1B, previously known as “EX4484-36”.

**Description of ‘*Candidatus* Penumbrarchaeia’ class nov.**

*Candidatus* Penumbrarchaeia (Pe.num.brar.chae.a'i.a. N.L. neut. pl. n. *Penumbrarchaeia* the *Penumbrarchaeum* class). The description is the same as for *Candidatus* Penumbrarchaeum gen. nov. with the following additions: -ia ending to denote a class. *Candidatus* Penumbrarchaeia class. nov. comprises the type order *Candidatus* Penumbrarchaeales ord. nov. and Order 2, Order 3 and Order 4, three previously unidentified orders in this class. Members of Order 3 and Order 4 are anaerobes inhabiting the water column of oxygen minimum and oxygen deficient zones, not capable of protein degradation based on their metabolic potential.

# **Supplementary methods**

## **Clone library construction**

In order to retrieve long sequences for phylogenetic analysis and quantitative PCR (qPCR) standard preparation, a clone library of archaeal 16S rRNA gene fragments (~800 bp) of the protein amended samples from the first generation enrichments (main methods section 1) was constructed. The primers 109F (5’-ACKGCTCAGTAACACGT-3’) [3] and 912R (5’‑GTGCTCCCCCGCCAATTCCTTTA-3’) [4] were used for PCR with the ALLin RPH polymerase Kit (highQu, Kraichtal, Germany) following the manufacturer’s protocol. Thermal cycling conditions included initial denaturation at 95°C for 10 min, followed by 28 cycles with denaturation at 95°C for 30 s, annealing at 52°C for 45 s, amplification at 72°C for 90 s, and final amplification at 72°C for 5 min. PCR products were purified using the Monarch PCR & DNA Cleanup kit (New England Biolabs, Frankfurt am Main, Germany), ligated into the pGEM-t vector (Promega, Mannheim, Germany) and transformed into *Escherichia coli* JM109 competent cells (Promega, Mannheim, Germany) according to the manufacturer’s protocol. DNA of randomly selected white colonies was extracted and subsequently PCR amplified with M13 primers (M13F-40; 5'-GTTTTCCCAGTCACGAC-3' and M13b; 5'-CAGGAAACAGCTATG-3') at the following thermal cycling conditions: initial denaturation at 95°C for 5 min, 30 cycles at 95°C for 30 s, 55°C for 45 s, 72°C for 45 s, followed by final amplification at 72°C for 10 min. The PCR was performed using AmpliTaq PCR kit (Applied biosystems, Carlsbad, USA). Amplicons of 64 clones were submitted to LGC Genomics (Berlin, Germany) for Sanger sequencing.

## **Standard preparation for qPCR**

Selected clone sequences affiliated with different archaeal groups were used for qPCR standard preparation. The 16S rRNA gene of previously extracted colonies (section I) was amplified with the primer set 109F (5’-ACKGCTCAGTAACACGT-3’) and 912R (5’-GTGCTCCCCCGCCAATTCCTTTA-3’) using the AmpliTaq PCR kit. PCR was performed using the following thermal cycling conditions: initial denaturation at 95°C for 5 min, followed by 30 cycles of denaturation at 95°C for 30 s, annealing at 58°C for 45 s and elongation at 72°C for 60 s, followed by final amplification at 72°C for 5 min. The resulting ~800 bp PCR product was analyzed by agarose gel electrophoresis and purified using the Monarch PCR & DNA Cleanup kit. Concentrations of standard DNA were quantified using the Quant-iT PicoGreen dsDNA assay kit (Thermo Fisher Scientific, USA). Each qPCR standard was diluted to a concentration of 0.5 ng/µl for further use.

## **qPCR primer design**

qPCR primers were designed for quantification of specific uncultured Thermoplasmatota groups present in our first- and second generation enrichments. Clone and sequences of amplicon sequence variants (ASV) of first (main methods section 1) and second (main methods section 2) generation enrichments were aligned using the Silva Incremental Aligner (SINA) v1.2.11 [5], imported into the ARB software v6.0.2 [6] and added to the SILVA 16S rRNA gene phylogenetic base tree (database version 138.1 Ref NR 99; [7, 8]) using the ARB Parsimony tool to retrieve their taxonomic placement. Based on their taxonomic placement, added clone sequences of the target group (class *Ca.* Penumbrarchaeia) were selected in the SILVA 16S rRNA gene phylogenetic base tree. Additionally, clone sequences from other archaeal phyla were selected as outgroup. For qPCR primer design, Primer Prospector v1.0.1 [9] was used to calculate possible primer sequences. Calculated primers were checked in ARB for their theoretical specificity against sequences of other closely related groups, selecting only primer sequences with at least two mismatches for non-*Ca.* Penumbrarchaeia taxa in the forward and at least 1 mismatch in the reverse primer. Resulting primer sequences 472f (5’-CGGTAAATCTCTGGGTAAATCG-3’) and 633r (5’-ACCCGTTCTGGTCGGACGCYTT-3’) were selected. The primer pair was validated for high specificity against the newly prepared Thermoplasmatota standard (positive control) and other standards (negative controls) of uncultured Thermoplasmata C1*,* Lokiarchaeia (Loki-2b), Bathyarchaeia (Bathy-8, Bathy-15), ANME-1, SG8-5 and MBGD. Further, a test for efficiency of the new Thermoplasmatota standard at annealing temperatures ranging from 58°C to 64°C was performed, followed by a melting curve stage after PCR, determining the best annealing temperature at 64°C with a final primer concentration of 300 nM and an amplification efficiency of 98.2%. Similarly, a new primer was created for Lokiarchaeia subgroup 2b. The primer pair was validated for high specificity against a Loki-2b standard (positive control) obtained in a previous study [10] and other standards (negative controls) of Lokiarchaeia (Loki-2c, Loki-3), Bathyarchaeia (Bathy-8, Bathy-15), ANME-1, SG8-5 and MBGD. The efficiency tests resulted in the best annealing temperature at 60°C and an amplification efficiency of 100%.

## **16S rRNA gene phylogenetic tree**

For the 16S rRNA gene phylogenetic tree, a total of 10,769 pre-aligned Thermoplasmatota sequences with a minimum sequence length of 1300 bp, pintail quality > 30, sequence quality > 50 and alignment quality > 50 were downloaded from SILVA (database version 138.1 Ref NR 99; [7, 8]). Further, 16S rRNA genes were extracted from all redundant Thermoplasmatota MAGs (main methods section 7) and all found *Ca.* Penumbrarchaeia MAGs (main methods section 8) using barrnap v0.9 [11] and aligned using SINA v1.2.11 [5]. Aligned sequences were manually refined in the built-in SINA alignment tool in ARB v7.1. All additional sequences were added to the SILVA 16S rRNA gene phylogenetic base tree (database version 138.1 Ref NR.99) in the ARB software v7.1[6-8] using ARB parsimony to augment underrepresented Thermoplasmatota groups. Additional to the sequences retrieved from the data-mined Thermoplasmatota and *Ca.* Penumbrarchaeia MAGs, a total of 354 reference sequences with an alignment length of 1303 bp were selected for de-novo phylogenetic tree reconstruction from the base tree. As outgroup 10 sequences from Halobacteriota were selected. A GTR+I+G4 model for the 16S rRNA gene phylogenetic tree was determined using modeltest-ng v0.1.7 [12], which was further used for calculation of the tree with raxml-ng v1.1.0 [13]. In total 50 starting trees were inferred; bootstrap convergence at a cutoff of 0.03 was reached after 1300 trees. Shorter 16S rRNA gene sequences found in *Ca.* Penumbrarchaeia MAGs, along with ASVs from the enrichments were added after tree calculation. Short sequences were aligned to the tree alignment with mothur v1.45.3 [14] and placed into the existing tree using epa-ng v0.3.8 [15] and gappa v0.7.1 [16]. Nucleotide blast [17] was used to calculate the similarity between the *Ca.* Penumbrarchaeia 16S rRNA gene ASV sq2 and the 16S rRNA gene sequence found in the *Ca.* Penumbrarchaeia MAG retrieved from the enrichment on day 157 (main methods section 6, sequences provided on zenodo (10.5281/zenodo.10813815).

## **Data collection, processing and MAG reconstruction in OMDB (v2)**

A total of 209 publicly available studies were gathered from selected marine metagenomics literature and matched with BioProject identifiers from the European Nucleotide Archive (ENA; Table S17). For this collection, raw read data were downloaded from ENA, and metagenomic data processing was performed as described in Paoli, et al. [18]. Briefly, sequencing raw reads were filtered using bbmap v.38.06 [19] by removing sequencing adapters from the reads, filtering out reads that mapped to quality control sequences (PhiX library), and discarding low quality reads using the parameters *trimq =*14, *maq =*20, *maxns =*1, and *minlength =*45. Additionally, read sets from Tara expeditions and from samples that required > 2TB of RAM in the subsequent assembling step were normalized with *bbnorm.sh target =*40 and *mindepth =*0. All metagenomes were individually assembled with metaSPAdes (versions from 3.11 to 3.15, depending on when the assembly was performed) [20]. For MAG reconstruction, quality-controlled metagenomic reads from at least 50 samples in OMDB (v2) were individually mapped against the scaffolds (≥ 1 kbp) of each sample. Reads were mapped with BWA v.0.7.17-r1188 [21], allowing reads to map at secondary sites (with the *-a* flag). Then, alignments were filtered by length (≥ 45 bp), with identity and coverage of the read sequence values of ≥ 97% and ≥ 80%, respectively. The resulting BAM files were processed using the *jgi_summarize_bam_contig_depths* script of MetaBAT 2 v.2.12.1 [22] to provide within- and between-sample coverages for each scaffold. The scaffolds were finally binned by running MetaBAT 2 on all samples individually with parameters *--minContig* 2000 and *--maxEdges* 500 for increased sensitivity. The quality of each metagenomic bin was evaluated using both the ‘lineage workflow’ of CheckM v.1.1.3 [23] and anvi’o v.7.1 [24]. Resulting MAGs with CheckM completeness ≥ 50% and contamination ≤ 10% or anvi’o completion ≥ 50% and redundancy ≤ 10% were taxonomically annotated using gtdbtk v.2.1.0 [25] with the default parameters against the GTDB v207 release [26]. These MAGs are available at [27].

**Supplementary results and discussion**

## **Additional qPCR results**

During the incubation time of 157 days (second generation), gene copies of Lokiarchaeia increased to 5.02 x 10^7^ gene copies per ml slurry. In the second biological replicate, Lokiarchaeia remained the most abundant group throughout the whole incubation period (Fig. S2). With prolonging incubation time, *Ca.* Penumbrarchaeia decreased in the second generation once the abundance of Lokiarchaeia had increased.

## **Delineation of the class *Ca*. Penumbrarchaeia**

Since 12 out of the 35 *Ca.* Penumbrarchaeia MAGs could not be placed at order or family level into the established prokaryotic taxonomy at the time (GTDB v207, [26]), we revised the taxonomy of the class *Ca*. Penumbrarchaeia using relative evolutionary divergence (RED) based on phylogenomic tree reconstruction (Fig. 3, Fig. S4, Table S5), average nucleotide identity (ANI) (Fig. S5) and amino acid identity (AAI) (Fig. S6). RED thresholds were adopted from the GTDB statistics for releases v207 [28] and v214 [29]: class ~0.25-0.46, order ~0.42-0.62, family ~0.63-0.84. Based on these thresholds Family 1A and 1B are sorted into one order (Order 1, RED: 0.47), Order 2 divides from Order 1 with a RED of 0.331. Order 3 and Order 4 branch with RED values of 0.286 and 0.253, respectively. The AAI thresholds derived from [30] were then used to confirm the RED-based classification into four orders and six families (Family 1A: 48- >90% AAI, Family 1B: 60- >90% AAI, Family 2: 60% AAI, Family 3A: 53- >90% AAI, Family 3B: >90% AAI, Family 4: >90% AAI). Genomes of Order 4 that initially branched with a quite low RED value, had an AAI of 41-42% compared to all other *Ca*. Penumbrarchaeia genomes and, based on these values, are part of the class *Ca*. Penumbrarchaeia. Computed ANI was further used to gain insights into species distinctions, using same thresholds as the GTDB (same species ANI >95%) [26].

## **Annotation of the class *Ca.* Penumbrarchaeia**

An overview of the full annotation can be found in Table S11. We further provide metabolic reconstructions based on annotated genes for each single order (Fig. S12-15). For simplification, orders are named by numbers. Order 1 corresponds to *Ca*. Penumbrarchaeales, family 1A to the family *Ca*. Penumbrarchaeaceae.

### **Carbon metabolism**

All orders encoded genes affiliated with peptide and amino acid degradation (Table S11). Families 1A, 1B and 2 encoded genes for extracellular peptidases (peptidase families C11A, M14B, S08A) (Fig. S11a). Moreover, all families encoded genes for oligopeptide transporters, the neurotransmitter:Na^+^ symporter of the NSS family, which can catalyze the uptake of nitrogenous substances, such as amino acids and osmolytes [31], different aminopeptidases (*pepF, pepT, pepP, pepS, map*) and aminotransferases (Table S12, Fig. S11b). All orders encoded the aspartate aminotransferase (*aspB*) and alanine aminotransferase (*alaA*). Single MAGs encoded an alanine-glyoxylate transaminase (AGXT2), branched-chain amino acid aminotransferase (*ilvE*) and aromatic amino acid aminotransferase. After deamination, the resulting 2-oxoacids could be further converted to acetyl-CoA via pyruvate ferredoxin oxidoreductase (*por*), or to acyl-CoA via indolepyruvate ferredoxin oxidoreductase (*ior*), 2-oxoacid:ferredoxin oxidoreductase (*kor*) or 2-oxoisovalerate ferredoxin oxidoreductase (*vor*). Acyl-CoA could further be hydrolyzed in substrate-level phosphorylation by an ADP-forming acetyl-CoA synthetase (*acdAB*) to form corresponding organic acids. The genes for ADP-forming acetyl coenzyme A synthetase were found in all orders. In four of the families (1B, 2, 3A and 4) also genes for succinyl-CoA synthetase (*sucCD*) were found, which could catalyze the conversion of succinyl-CoA to succinate.

The families 2, 3A and 4 additionally encoded all genes of the beta-oxidation pathway (Fig. S17), including a butyryl-CoA dehydrogenase (ACADS), acyl-CoA dehydrogenase (ACADM), enoyl-CoA hydratase (*crt*), 3-hydroxyacyl-CoA dehydrogenase (*fadB*) and acetyl-CoA acyltransferase (*fadA*) to further degrade short and medium chain acyl-CoAs. The amino acid degradation would result in the main products being organic acids. Family 1B additionally contained a lactate dehydrogenase (*ldhA*), with which lactate could be formed from pyruvate.

MAGs of the class *Ca.* Penumbrarchaeia contained between 2 to 14 different carbohydrate active enzymes (CAZymes), with most CAZymes being annotated as glycosyl transferases of family GT2 and GT4 (Table S12). Glycosyltransferases are involved in biosynthesis of glycosidic bonds and as such not involved in the metabolic degradation of sugars [32]. Some MAGs also encoded glycoside hydrolases, which hydrolase glycosidic bonds and might therefore be involved in carbohydrate degradation. Within our dataset only 21 of 35 MAGs encoded different glycoside hydrolases. Most of the MAGs contained between one and three glycoside hydrolases (Table S13). Overall, these low CAZyme counts for archaea have been observed before [33] and demonstrate the limited potential of archaea in carbohydrate degradation. While some MAGs found in this study might take part in the breakdown of carbohydrates, we did not observe a common feature for all families.

All orders within the class *Ca*. Penumbrarchaeia encoded genes for a partial reverse citric acid (rTCA) cycle, including an ATP-citrate lyase (*aclAB /* ACLY), aconitate hydratase (ACO) and isocitrate dehydrogenase (*idh*) (Table S11). The ATP-citrate lyase, catalyzing the cleavage of citrate into acetyl-CoA and oxaloacetate, is regarded as key enzyme for the rTCA cycle [34]. Additionally, most of the families encoded genes for fumarate hydratase (*fum*) and malate dehydrogenase (*mae*). Only all MAGs in families 2 and 4 encoded genes for 2-oxoglutarate ferredoxin oxidoreductase (*korABCD*), succinyl-CoA synthetase (*sucCD*) and succinate dehydrogenase (*sdhAB*), therefore possessing a complete rTCA cycle. During the rTCA cycle, CO_2_ is fixed in conversions of succinyl-CoA to 2-oxoglutarate and in conversion of 2-oxoglutarate to isocitrate [35]. For other carbon fixation pathways, namely the 3-Hydroxypropionate cycle, 3-Hydroxypropionate/4-Hydroxybutyrate cycle or Dicarboxylate/4-Hydroxybutyrate cycle no complete pathways were encoded in any of the families (Table S11). The presence of inorganic carbon fixation via the rTCA was previously suggested for other groups within the Thermoplasmatota, such as Thermoplasmatota_A [35], which clustered closest to the *Ca.* Penumbrarchaeia class in our phylogenomic tree (Fig. 3a) and the *Ca*. Proteinoplasmatales [36].

In contrast to any other family, genes involved in the Wood Ljungdahl pathway could only be detected in family 3A (Fig. S14). All of the MAGs contained genes for acetyl-CoA decarbonylase/synthase, CODH/ACS complex subunit beta, gamma and delta (*cdhCDE*). Besides, genes for methylenetetrahydrofolate reductase (*metF*), methylenetetrahydrofolate dehydrogenase (*folD*), formate--tetrahydrofolate ligase (*fhs*), 5-methyltetrahydrofolate corrinoid/iron sulfur protein methyltransferase (*acsE*) and the alpha subunit of formate dehydrogenase (*fdhA*) were present. However, the catalytic subunit of anaerobic carbon-monoxide dehydrogenase, catalyzing the reduction of CO_2_ to CO could not be detected in any of the MAGs. As all MAGs within this family have a completeness between 80-91%, these missing genes in the Wood Ljungdahl pathway might be due to incomplete genomes.

### **Carbon assimilation**

Carbon might be assimilated through gluconeogenesis and the pentose phosphate pathway (PPP) to form nucleic acids via phosphoribosylpyrophosphate (PRPP). Pyruvate formed through degradation of amino acids or formed from acetyl-CoA during amino acid breakdown via the rTCA cycle might be converted via the gluconeogenesis pathway to glyceraldehyde-3P. All families encoded genes for pyruvate dikinase (*pps*), enolase (*eno*), phosphoglycerate mutase (*gpmA*) or 2,3-bisphosphoglycerate-independent phosphoglycerate mutase (*gpmM*), phosphoglycerate kinase (*pgk*) and glyceraldehyde-3-phosphate dehydrogenase (*gap*) (Table S11, Fig. S12-15). Further, all families, except family 3B, encoded all genes of the non-oxidative PPP, including a transketolase (*tkt*), transaldolase (*tal*), ribose-5-phosphate isomerase (*rpi*), ribulose-phosphate 3-epimerase (*rpe*) and ribose-phosphate pyrophosphokinase (*prsA*). Family 3B lacked genes of the transketolase, transaldolase and ribulose-phosphate 3-epimerase. However, family 3B was the only family, which encoded a glucokinase (*glk*), suggesting that the family might use either the glycolysis or the gluconeogenesis pathway. Since neither genes for the pyruvate kinase (*pyk*) nor the fructose-1,6-bisphosphatase (*fbp*) could be found, no clear prediction could be made for the carbon assimilation potential of this family.

### **Hydrogenases and energy conservation**

Multiple subunits of the oxidative phosphorylation complex I were only encoded in family 1A. Genes for two of four subunits of the succinate dehydrogenase / fumarate reductase (*sdhAB*) were found in families 2 and 4, those families which encoded a full rTCA cycle. Of complex V multiple subunits of the V/A-type H+-transporting ATPase (A_1_A_0_-ATP synthase, *atpABCDEFGIK*) for energy conservation through a sodium gradient were encoded by all families, with most families lacking subunits *atpE* and *atpG*. None of the families encoded genes involved in the oxidative phosphorylation complexes III and IV (Table S11). The lack of most genes involved in oxidative phoyphorylation suggests an anaerobic lifestyle for the class *Ca.* Penumbrarchaeia.

All families encoded genes for a K(+)-stimulated pyrophosphate-energized sodium pump (*hppA*), which could couple the hydrolysis of pyrophosphate to the transport of sodium across the membrane against an electrochemical gradient [37, 38]. The sodium gradient could further be used to form ATP via the detected ATPase [39]. Moreover, all MAGs of families 3A, 3B and 4 contained genes for the H+/Na+-translocating ferredoxin:NAD+ oxidoreductase (*rnfABCDEG*), which oxidizes reduced ferredoxin and reduces NAD for energy conservation, while transporting ions across the cytoplasmic membrane [40]. The sodium gradient resulting from the *rnf* complex could be further used by the encoded V/A-type H+-transporting ATPase (A_1_A_0_-ATP synthase), as has been hypothesized previously [40].

All families encoded genes for hydrogenase nickel incorporation proteins and hydrogenase expression proteins (*hypABCDEF*) required for biosynthesis and Ni insertion of NiFe hydrogenases [41]. Genes for NiFe group 3b sulfhydrogenases were present in most of the families. However, in most families only genes for two subunits, which function as hydrogen dehydrogenase (*hydAD*) were found. Specifically, family 4 lacked the beta and gamma subunits (*hydBG*), which function as sulfur reductase [42], and family 2 did not encode any subunit of the sulfhydrogenase. The sulfhydrogenase was shown to catalyze the reduction of elemental sulfur or polysulfides to hydrogen sulfide and the oxidation or reverse reaction of hydrogen with NAD(P)+ as electron acceptor in the absence of sulfur [43, 44]. As no other sulfur related genes were found in any of the MAGs, it is most likely that the sulfhydrogenase might function as a hydrogen dehydrogenase only.

MAGs of families 1A, 2, 3A and 4 further encoded the NiFe group 3c F420-non-reducing hydrogenase (*mvhADG*) and heterodisulfide reductase (*hdrABC*). While this hydrogenase is usually associated with methanogenic archaea [45-48], in the *Ca*. Penumbrarchaeia class it might rather be involved in the reduction of ferredoxin and an unknown disulfide reducer, coupled to the oxidation of hydrogen, as was also suggested for *Ca*. Lokiarchaeum prometheoarchaeum [49] and the hyperthermophilic Panguiarchaeum symbiosum [50].

Lastly, MAGs of the families 1A and 1B encoded three of the fourteen subunit containing NiFe group 4d membrane bound hydrogenase (mbh), which was suggested to function as a redox-driven ion pump, generating a proton motive force through reduction of protons with a low-potential ferredoxin, thereby producing hydrogen [51]. Along with the three found hydrogenase subunits, MAGs of families 1A and 1B encoded multicomponent Na^+^:H^+^ antiporter subunits (mnhBCDEFG), and gene homologs of NADH-quinone oxidoreductase (nuoBCDEFH), which were shown to have similarities to multiple subunits of the NiFe group 4d membrane bound hydrogenase [51]. Genes of multicomponent Na^+^:H^+^ antiporter subunits, NADH-quinone oxidoreductase and the found membrane bound hydrogenase subunits were located along single contigs, possibly forming operons, which suggests that MAGs of order 1 might contain a mbh like hydrogenase.

### **Transporters**

Besides various ABC transporters, all families contained genes for ABC-2 type transport system ATP-binding proteins and ABC-2 type transport system permease proteins, for which no function could be assigned (Table S11). Iron, an essential nutrient in microorganisms, is required for enzymatic processes and as cofactor for proteins involved in respiration, oxidative stress resistance or gene regulation [52]. Genes encoding the iron (II) transport system (*feoAB)* could be found in families 1A, 1B, 3B and 4. MAGs of families 2 and 3A only encoded subunit *feoB*. Ferrous iron in its reduced form is only present in oxygen-limiting, anoxic and low pH conditions [53]. Since these families only encoded a ferrous iron transport system and are present in marine sediments, in which neutral pH is prevalent [54-56], the MAGs found in the *Ca.* Penumbrarchaeia class most likely live in anaerobic conditions [57].

Similarly to iron, zinc functions as cofactor in enzymatic reactions [58]. MAGs of families 1A, 1B and 4 encoded the cytoplasmic membrane zinc transporter *zupT*, which is responsible for the uptake of zinc and other metal ions [59]. Magnesium, another cofactor in enzymatic reactions and involved in the catalysis of ribozymes [60], RNA splicing and stabilization of proteins and RNA [61], is transported into the cell via the magnesium transporter *corA* [62, 63]. Genes for *corA* were found in families 1A and 2. Genes encoding a transporter for cobalt and nickel (*cbiMOQ*) could only be found in family 4. Nickel and cobalt also function as cofactors in enzymes, such as in NiFe hydrogenases [64] or in the corrin ring of coenzyme B12 [65].

Moreover, all families except family 4 encoded genes for a low-affinity inorganic phosphate transporter of the PiT family. Inorganic phosphate acts as a key nutrient in cells, as it is important for cellular building blocks, such as nucleic acids, phospholipids, teichoic acids and membranes. MAGs of family 1A and 1B additionally encoded genes for a high affinity phosphate transport system (*pstSABC*), which is induced in low phosphate concentrations. An accompanying *phoU* regulon was encoded and is required for the repression of the phosphate transport system *pstSABC* at high phosphate conditions to avoid uncontrolled phosphate uptake [66-68], therefore actively controlling phosphate transport. Furthermore, the *trk* system potassium uptake protein (*trkAH*) was encoded in all families, except family 4. Potassium plays a role in cellular homeostasis, osmotic tolerance, pH stress response and membrane potential maintenance [69, 70]. The *trkAH* was shown to act as an ATP- and ADP-gated ion channel, with *trkH* being classified as potassium transport protein in low external potassium concentrations [71]. MAGs of family 1B and 3A additionally contained genes for the high affinity molybdate/tungstate transport system ATP-binding protein *wtpABC [72]*, while MAGs of family 4 encoded genes for the lower affinity tungstate transport system *tupABC* [73]. A gene for a tungstate transport system ATP-binding protein (*tupC*) was missing in all MAGs of family 4. Both, molybdate and tungstate are known to function as cofactors in enzymatic reactions [74, 75].

Along with a controlled import, cells actively export ions from the cell to maintain homeostasis. The families 1A, 2, 3A, 3B and 4 contained genes encoding a P-type calcium transporter (ATP2C). These P-type ion transporting ATPases transport cations across membranes, while coupling the transport to the hydrolysis of ATP [76].

Besides, MAGs of family 1A, 1B, 3A and 3B encoded an acetate uptake transporter (*satP*), which has an affinity for acetate and succinate [77]. Acetate can have a regulatory function for processes, such as motility and stress response [78]. Furthermore, acetate could be used as a carbon source for metabolic functions [78].

### **Stress response**

All families of the class *Ca.* Penumbrarchaeia contained genes related to environmental stress (Table S11). All families encoded genes for thioredoxin reductase (*trxR*), thioredoxin (*trxA*), and desulfoferredoxin (*dfx*), acting as superoxide reductase [79], all of which prevent oxidative stress. Moreover, in MAGs of all families, except family 4, genes for peroxiredoxin (*prxQ*) were found, which catalyzes the conversion of hydrogen peroxide to water [80]. Additionally, MAGs of all families encoded DnaK. This enzyme functions as Heat Shock Protein-70 (Hsp70), which protects cells from heat and oxidation [81, 82].

To maintain cellular homeostasis, MAGs encoded a sodium:calcium antiporter (*yrbG*), which actively removes calcium ions from the cell by using a sodium gradient [83]. Genes for *yrbG* were found in all families, except family 3A.

Additionally, MAGs of all families contained genes for the P-type Cu+ transporter (*copA*) involved in maintaining copper homeostasis [84, 85]. While copper, in low concentrations, is required for enzymatic function, excess copper can become toxic for the cell and therefore needs to be exported [86, 87]. Another enzyme involved in heavy metal resistance is the cobalt-zinc-cadmium efflux system protein (*czcD*), for which genes were found in families 1A, 1B, 3B and 4 [88].

Besides active transport of heavy metal ions from the cell, microorganisms developed other functions to reduce toxicity. Toxic arsenate, as a structural homolog of phosphate, can be imported into the cell through phosphate transporters [89]. MAGs of the families 1A and 2 contained genes for the reduction of arsenate via an arsenate reductase (*arsC*), which reduces arsenate As(V) to arsenite As(III). A gene encoding an arsenite transporter (acr3 / *arsB*) to remove arsenite from the cell was found in MAGs of family 1A, 2 and 3A. An arsenite methyltransferase (AS3MT) was additionally found in the MAGs of family 1A, 3A, 3B and 4. This enzyme has the capability to detoxify arsenic by methylation of the toxic compound. Most of arsenic found in the environments derives from anthropogenic activity and can be introduced into environments through rivers, surface run-off and munition [90-93]. Genes against arsenic toxicity were mostly found in MAGs deriving from the Baltic Sea, which was shown to contain localized high arsenic concentrations [94].

Lastly, all MAGs of family 1A, 2 and 4 contained genes annotated as the MATE family drug/sodium antiporter, a defensive mechanism against antimicrobial drugs, driven by a sodium force [95]. Besides antimicrobial resistance against naturally occurring antibiotics, MAGs of these families were found in near-shore environments, such as the Baltic Sea or the Cariaco Basin in Venezuela, which are experiencing input of waste water and with this, antimicrobial drugs, might be causing antimicrobial resistance in these environments [96, 97].

**
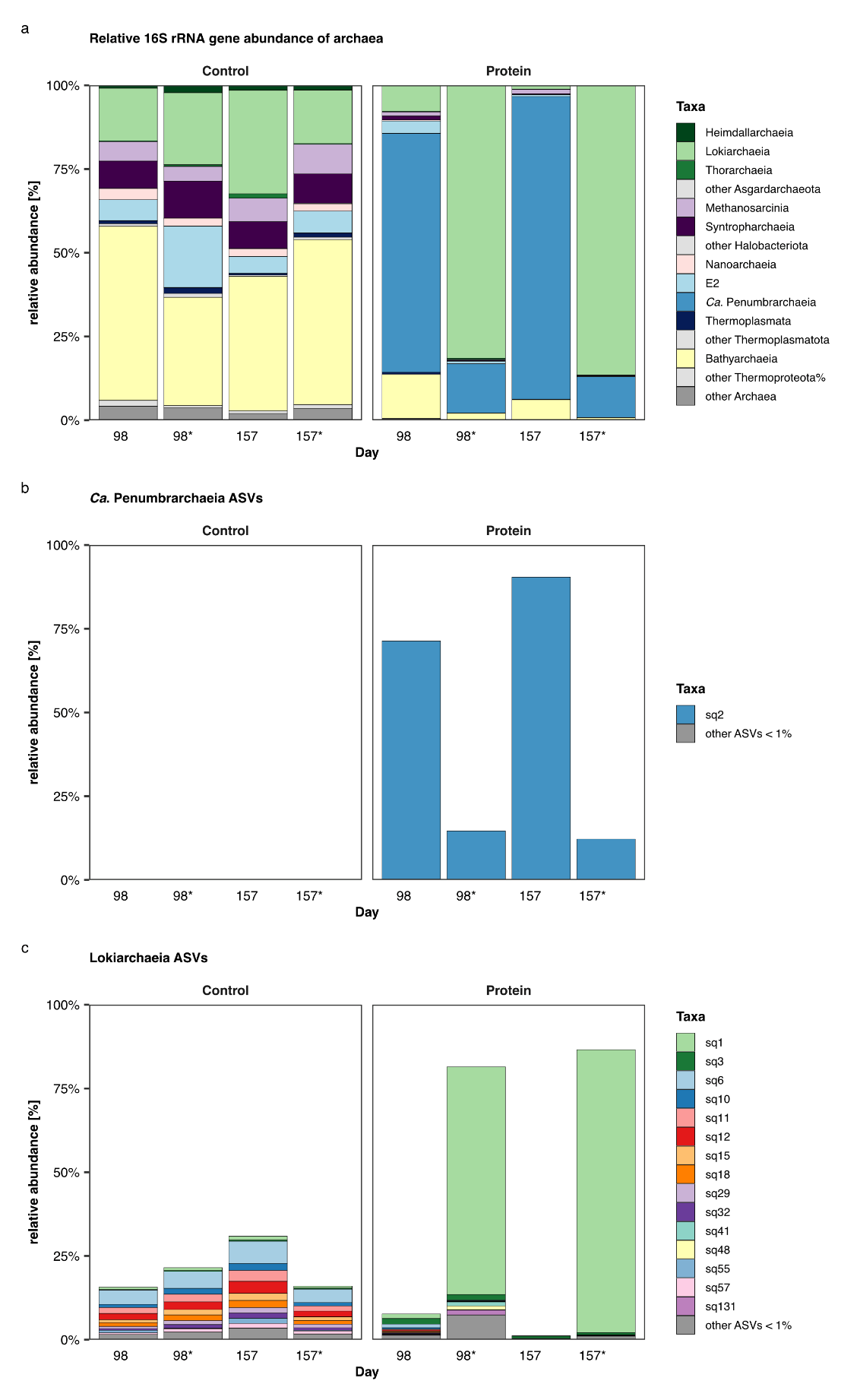
Supplementary Figures**

**S1. Microbial community composition of second-generation enrichments.** Relative abundances of (**a.)** archaeal 16S rRNA genes, (**b.)** *Ca.* Penumbrarchaeia ASVs and (**c.)** Lokiarchaeia ASVs at day 98 and 157 in two replicates each of control samples and protein amended samples, both amended with 30 mM sulfate (Na_2_SO_4_) and an antibiotics mix (D-cycloserin, kanamycin, vancomycin, ampicillin and streptomycin; 50 mg/l for each). Protein samples were additionally amended with 2.33 g/l egg white protein.

**
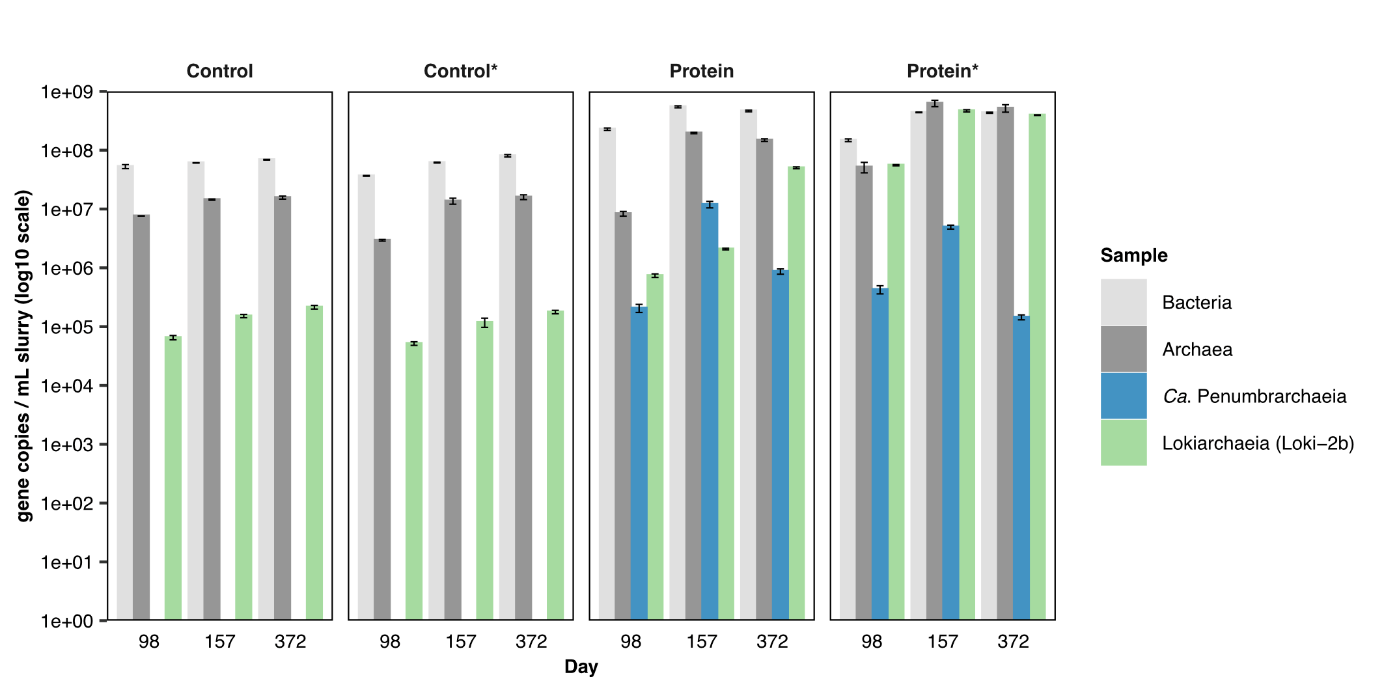
S2.**  **Microbial community composition of second-generation enrichments.** 16S rRNA gene copies per ml slurry of Bacteria, Archaea, the class *Ca.* Penumbrarchaeia and *Lokiarchaeia* subgroup Loki-2b. Gene copies are shown for both replicate enrichments (Replicate 1, Replicate 2*) of control samples and protein amended samples, both amended with 30 mM sulfate (Na_2_SO_4_) and an antibiotics mix (D-cycloserin, kanamycin, vancomycin, ampicillin and streptomycin; 50 mg/l for each). Protein samples were additionally amended with 2.33 g/l egg white protein. Error bars represent standard deviations of the three technical qPCR replicates per sample.


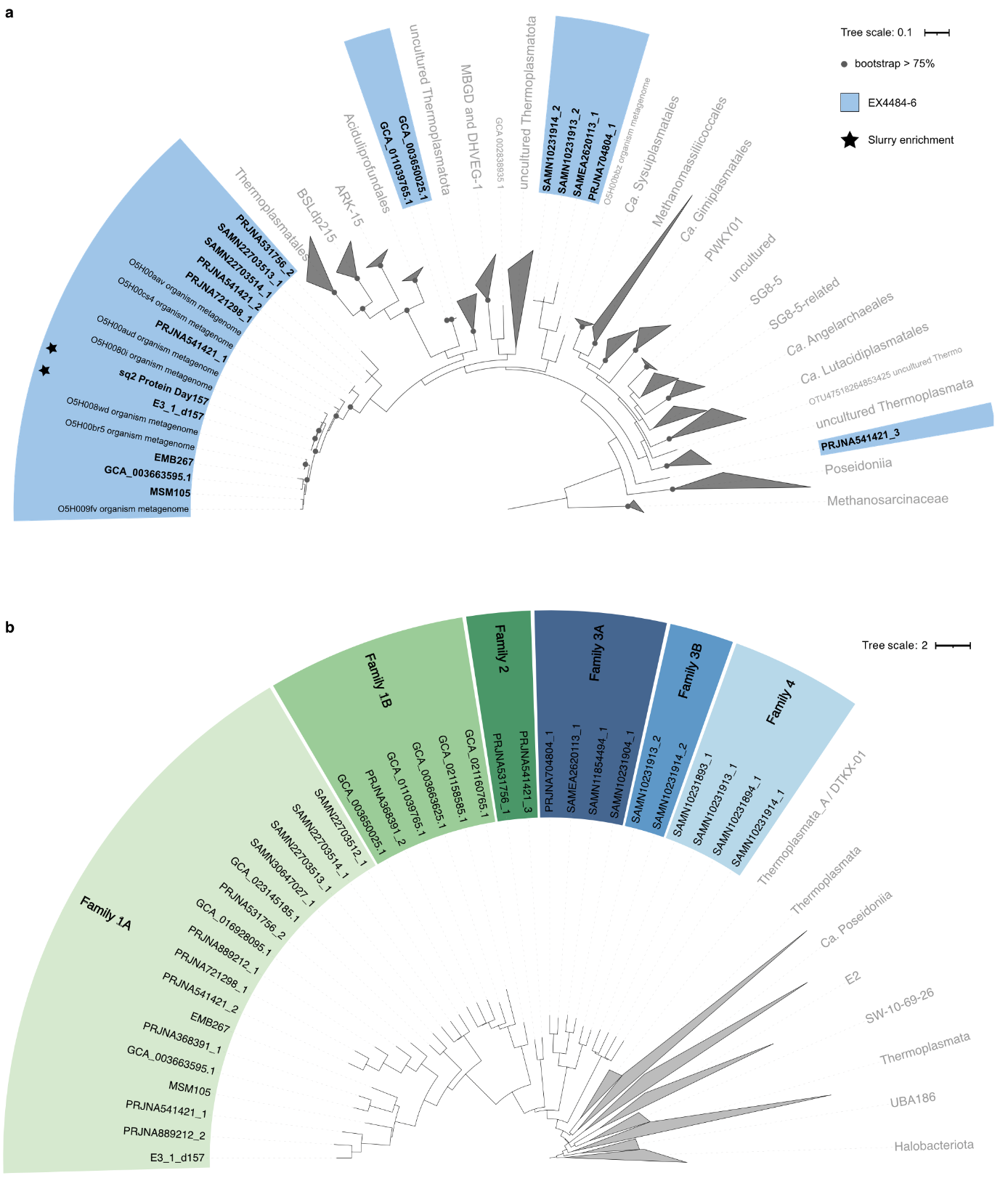


**S3. Phylogenetic reconstruction of the class *Ca*. Penumbrarchaeia.** **(a.)** Maximum-likelihood tree (RAxML, convergence reached after 1300 bootstraps) of 354 full length 16S rRNA gene sequences of Thermoplasmatota, including all retrieved full length *Ca*. Penumbrarchaeia 16S rRNA genes from single MAGs. Shorter 16S rRNA gene sequences of ASVs and those found in *Ca.* Penumbrarchaeia MAGs were added to the existing tree. **(b.)** Maximum-likelihood tree based on orthogroups that were part of the core genome and detected in > 90% of all *Ca*. Penumbrarchaeia MAGs. The species tree was inferred from gene trees of all orthogroups using SpeciesRax [98].


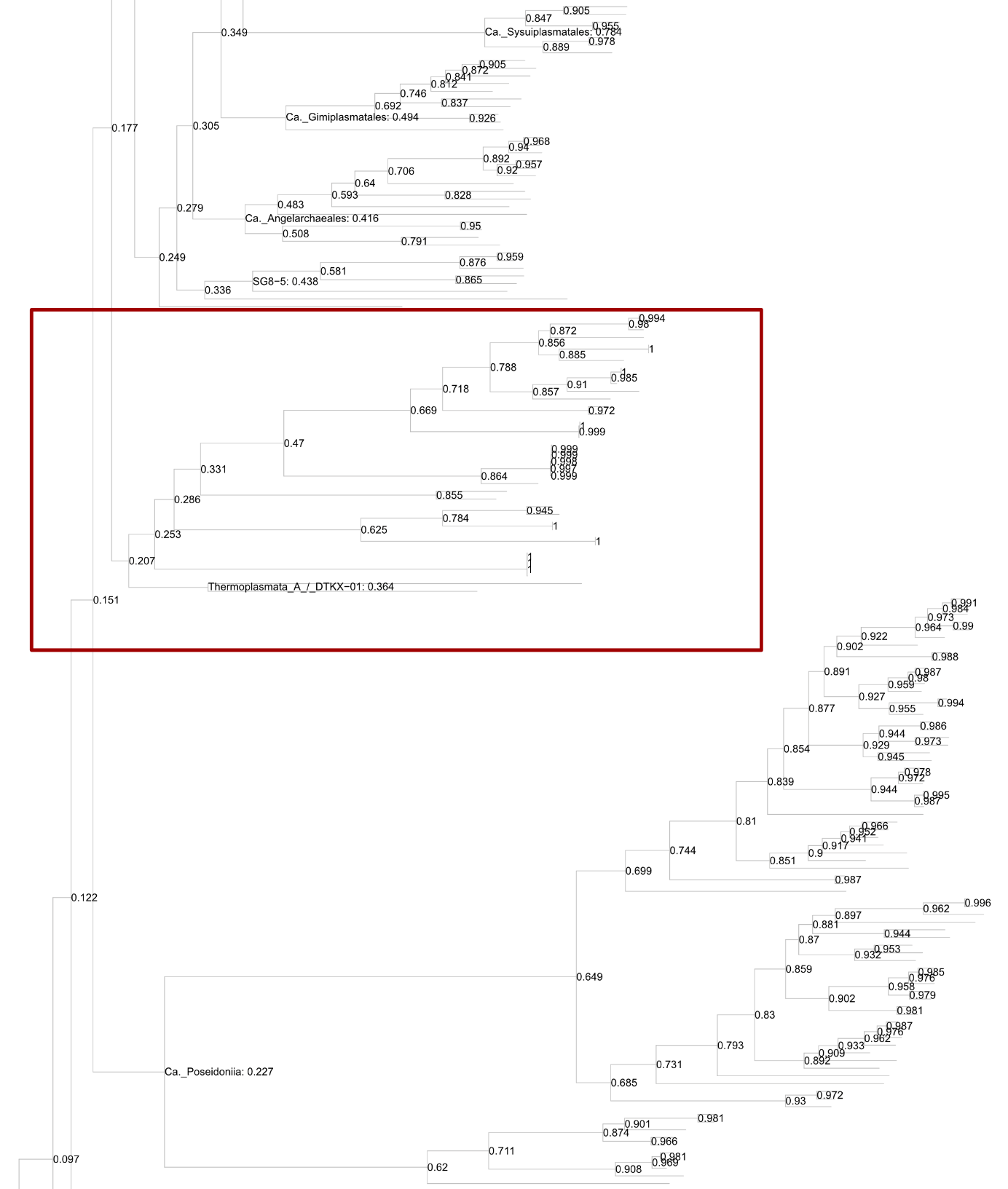


**S4. Partial phylogenomic tree of the Thermoplasmatota including RED.** Maximum-likelihood tree (RAxML, 100 bootstraps) of 370 Thermoplasmatota MAGs and 35 *Ca*. Penumbrarchaeia MAGs obtained through data mining of genome assemblies and metagenomic short read data sets. Node labels display taxonomic affiliation on class level. The class *Ca.* Penumbrarchaeia clusters with Thermoplasmata_A/DTKX_01 according to the marker gene tree and is indicated by a red box (Fig. 3a). Node values indicate relative evolutionary divergence (RED). The complete tree is provided as separate PDF.


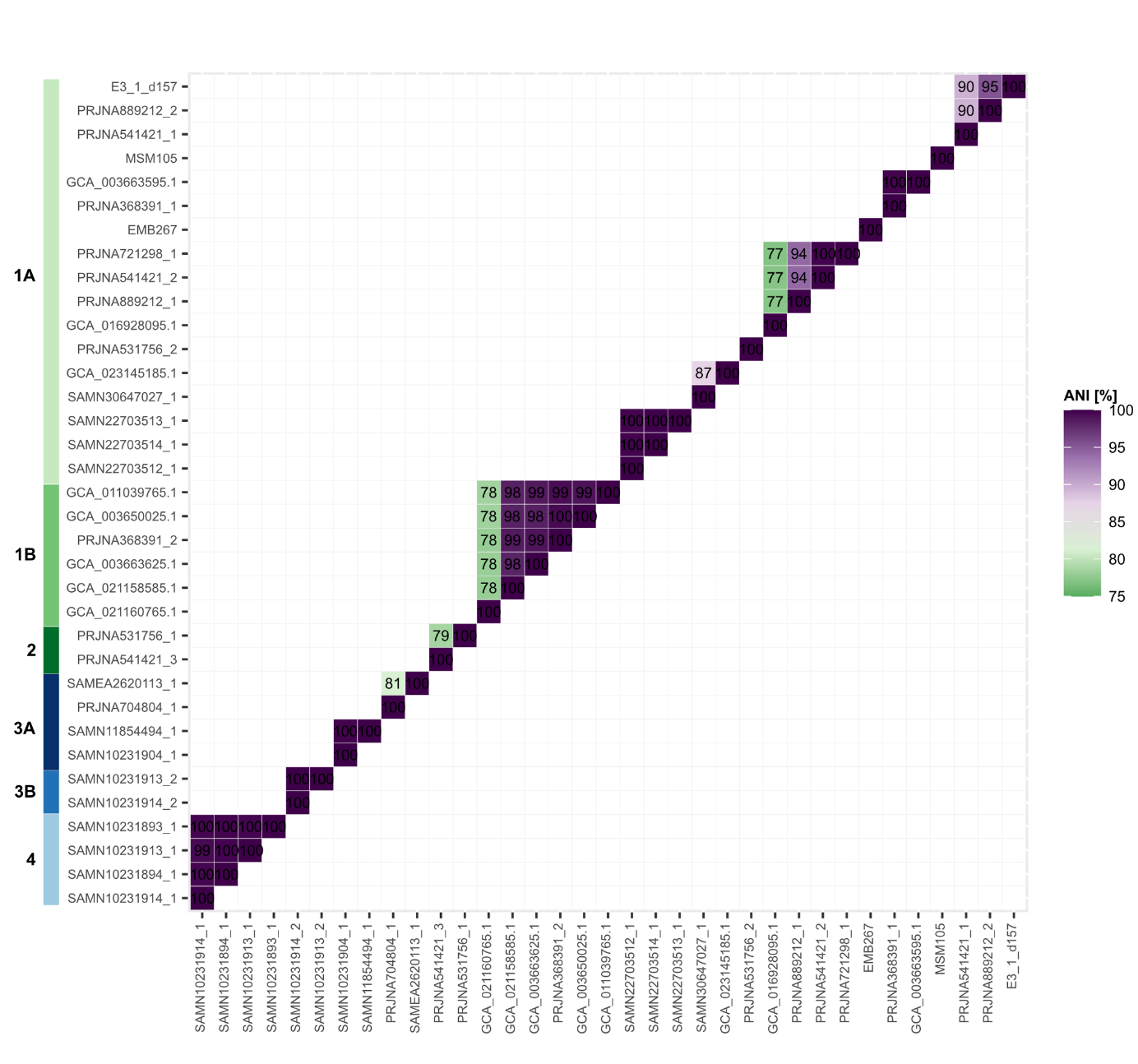


**S5. Average nucleotide identity (ANI).** Heatmap of ANI between all 35 *Ca.* Penumbrarchaeia MAGs obtained during our data mining. Numbers in colored tiles indicate the ANI percentage between the compared MAGs (threshold ANI > 75%). MAGs were ordered according to their taxonomy in the marker gene tree (Figure 3.


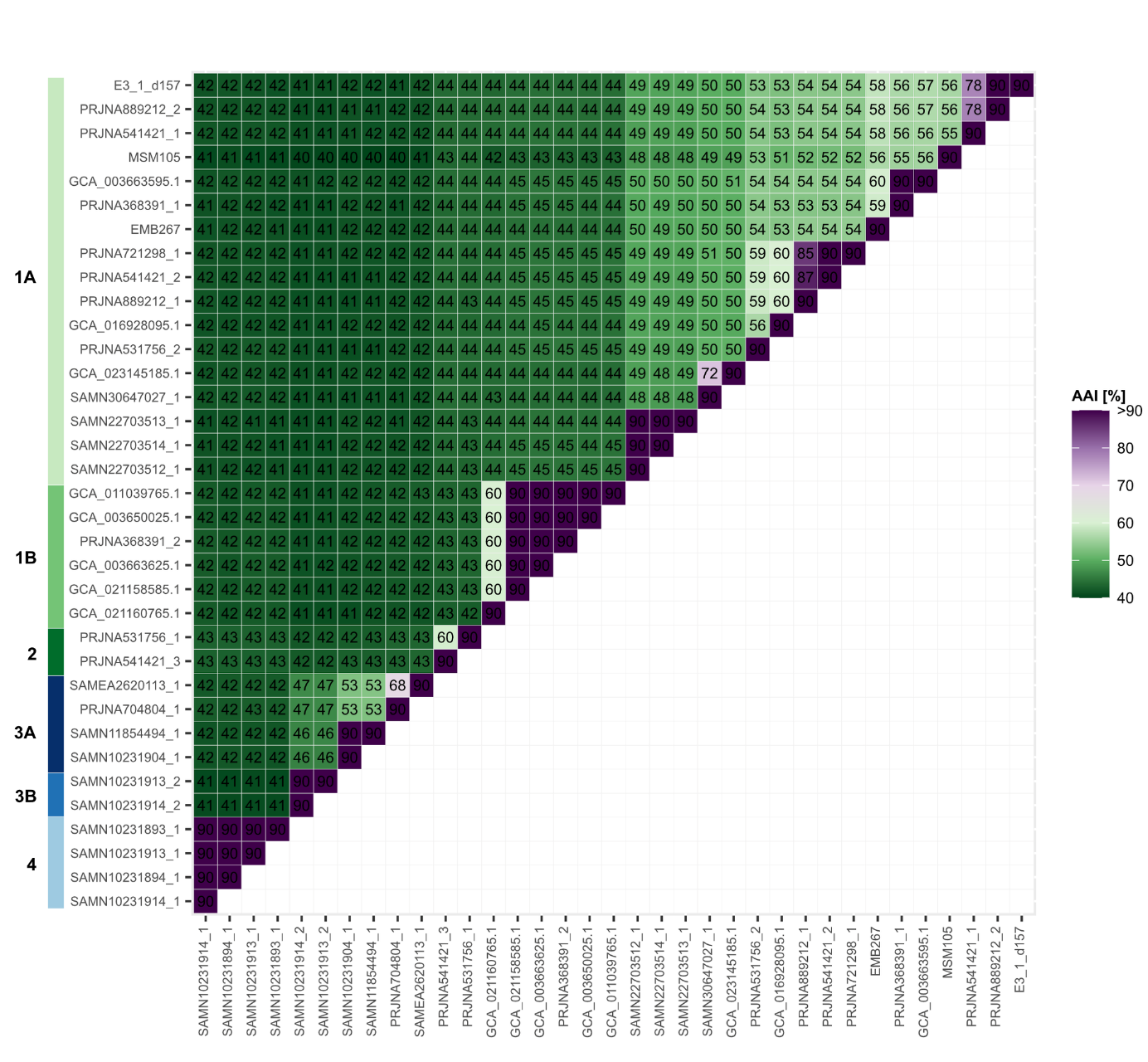


**S6. Amino acid identity (AAI).** Heatmap of AAI between all 35 *Ca.* Penumbrarchaeia MAGs obtained during our data mining. Numbers in colored tiles indicate the AAI percentage between the compared MAGs. MAGs were ordered according to their taxonomy in the marker gene tree (Figure 3).


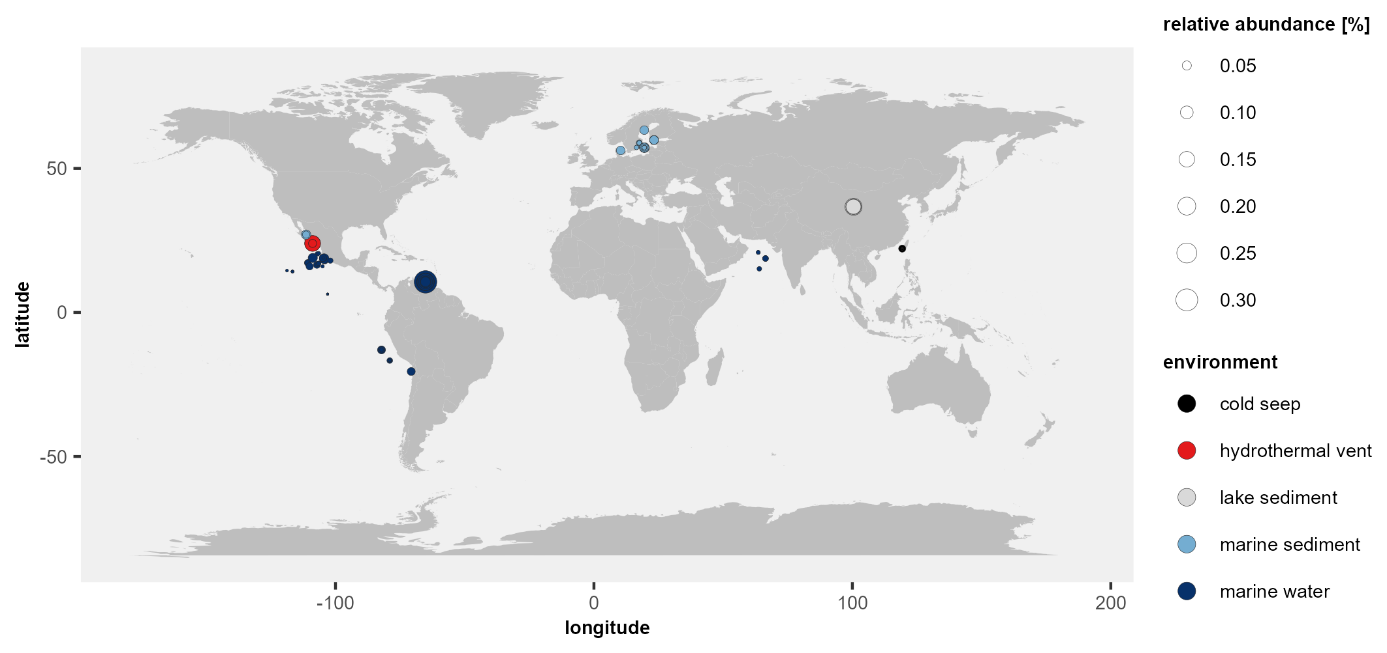


**S7. Distribution of the class *Ca.* Penumbrarchaeia.** World map showing the distribution, relative abundance and environment of all observed *Ca.* Penumbrarchaeia detected in 128 samples. Relative abundances were calculated by aligning quality trimmed reads of 8573 metagenomic sequencing runs to a competitive mapping index containing 20 non-redundant *Ca.* Penumbrarchaeia MAGs. Point colors indicate the environment, point sizes indicate relative abundance.

**
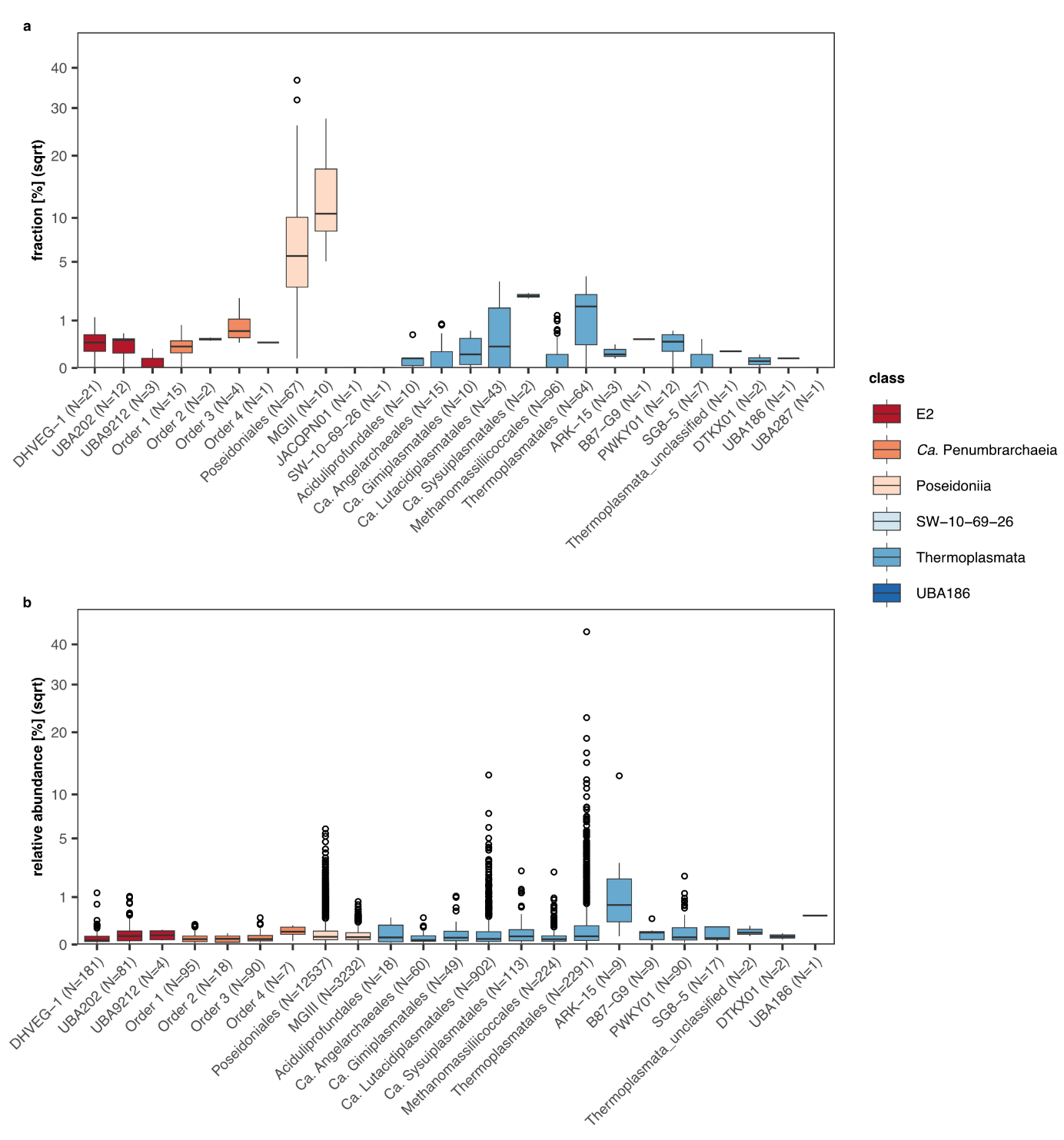
**

**S8. Abundance of non-redundant Thermoplasmatota in the environment. (a.)** As fraction of occurrence for each order found within the non-redundant Thermoplasmatota data set. The fraction of occurrence was defined as fraction of data sets MAGs occurred in across all screened data sets. Number of observations N represents the number of Thermoplasmatota genomes per order. (**b.)** As relative abundance of each order in samples they occurred in. Number of observations N represents the number of metagenomic runs, in which the genome was detected. Order 1 - Order 4 correspond to the *Ca*. Penumbrarchaeia orders, with Order 1 being *Ca.* Penumbrarchaeales.


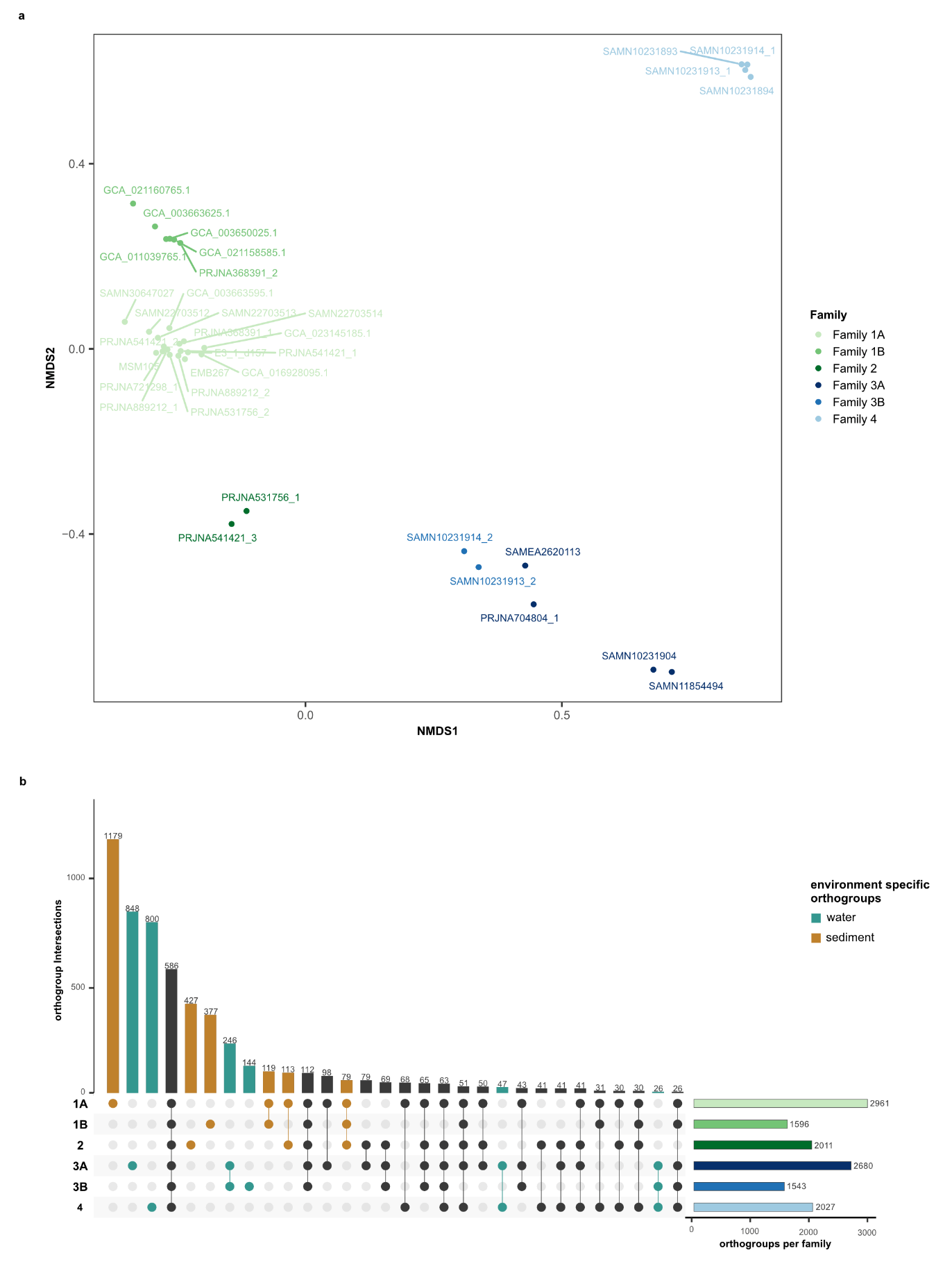
**S9. Clustering of *Ca.* Penumbrarchaeia MAGs based on orthogroups. a** NMDS based on all orthogroups found in all 35 *Ca.* Penumbrarchaeia. The NMDS was computed using the function metaMDS from the package vegan v2.6.4 with Jaccard dissimilarities and two dimensions. Single families are indicated by different colors. Family 1A corresponds to *Ca.* Penumbrarchaeaceae. **b** Upset plot showing intersections of orthogroups for all six families. Intersections shown reflect 95% of all orthogroups within the data set. Numbers of orthogroups per intersection are indicated above the bars. Orthogroup intersections, which are only present in sediment or water are indicated by color. Total number of different orthogroups per family are indicated by horizontal bars next to the upset plot.


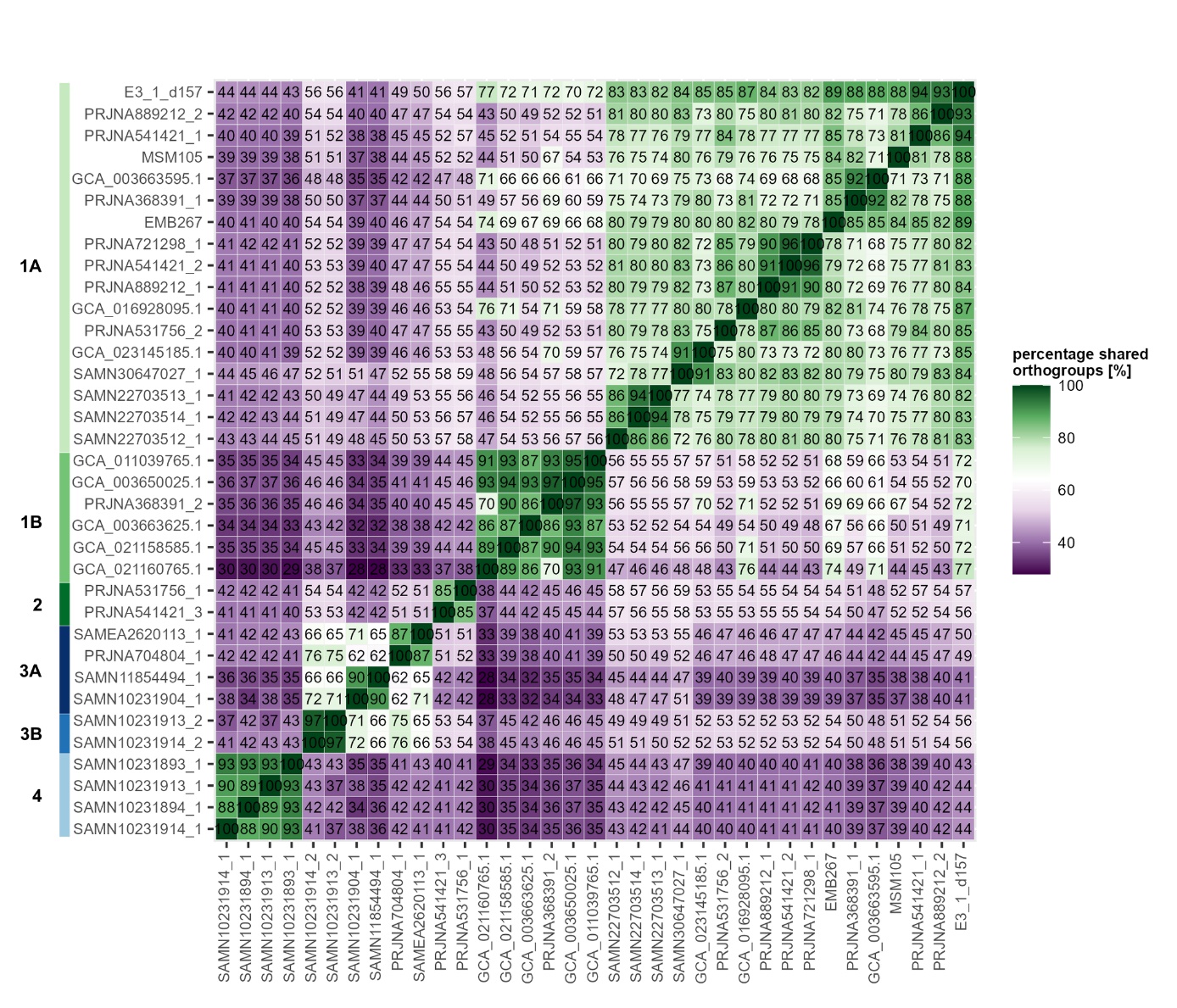


**S10. Shared orthogroups between *Ca.* Penumbrarchaeia MAGs.** Heatmap of shared orthogroups between all 35 *Ca.* Penumbrarchaeia MAGs. Numbers in colored tiles in the heatmap indicate the percentage of shared orthogroups between the compared MAGs. MAGs were ordered according to their family taxonomy in the marker gene tree (Figure 3). Family 1A corresponds to *Ca.* Penumbrarchaeaceae.

**
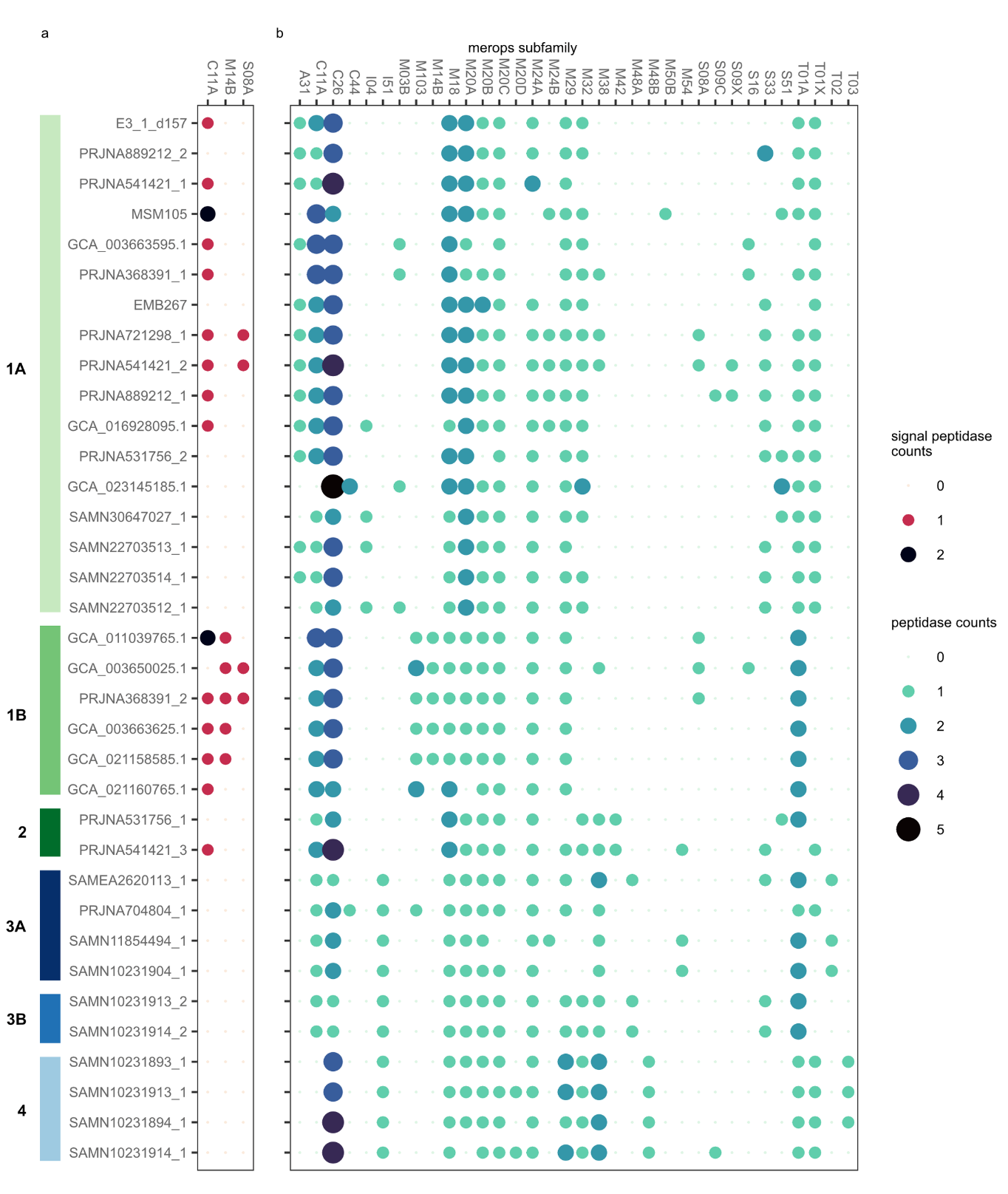
**

**S11. Peptidases in *Ca.* Penumbrarchaeia** **MAGs. a** Extracellular peptidase homologs within MAGs of the class *Ca*. Penumbrarchaeia. **b** Peptidase homologs within MAGs of the class *Ca.* Penumbrarchaeia. MAGs were ordered according to their family taxonomy in the marker gene tree (Figure 3). Family 1A corresponds to *Ca.* Penumbrarchaeaceae.

**
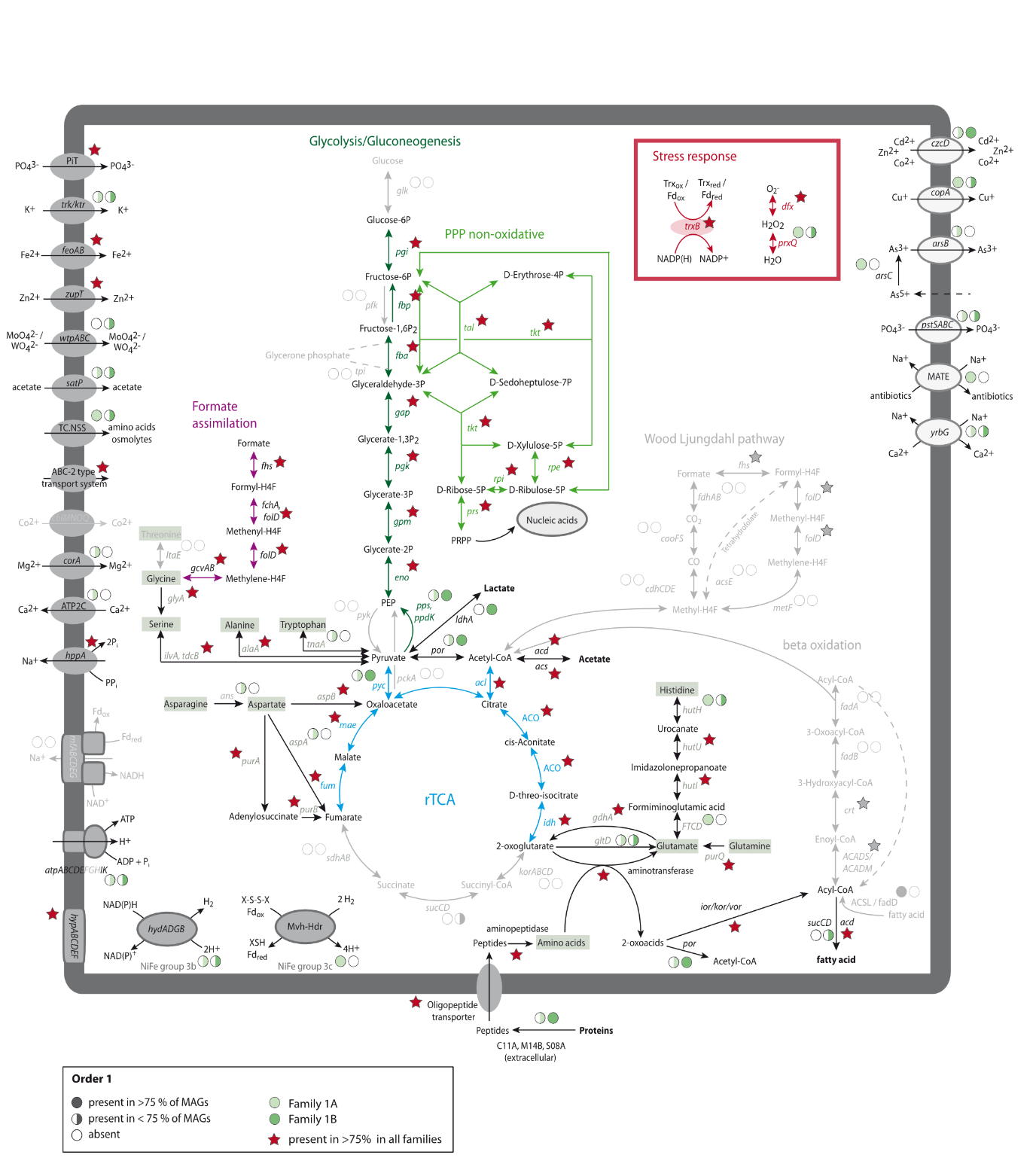
**

**S12. Metabolic reconstruction of *Ca*. Penumbrarchaeales (Order 1) (*Ca*. Penumbrarchaeaceae (Family 1A), Family 1B).** Pathways in grey represent pathways with missing genes that are therefore not functional. Gene abbreviations can be found in Supplementary table 9. The presence of genes is indicated by full or half circles for each family. Red stars indicate the presence of genes in all families.

**
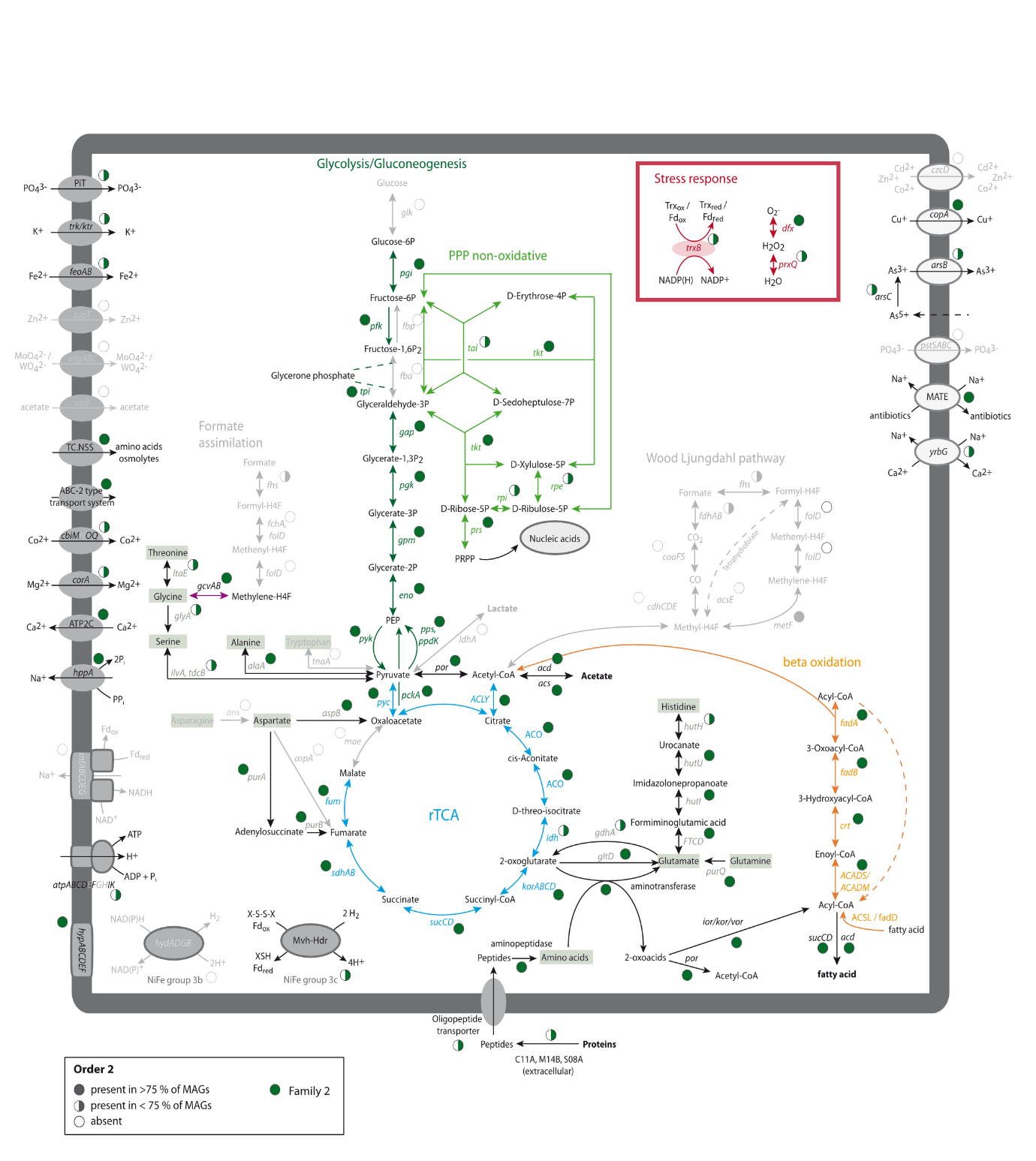
**

**S13. Metabolic reconstruction of Order 2 (Family 2).** Pathways in grey represent pathways with missing genes that are therefore not functional. Gene abbreviations can be found in Supplementary table 9. The presence of genes is indicated by full or half circles.

**
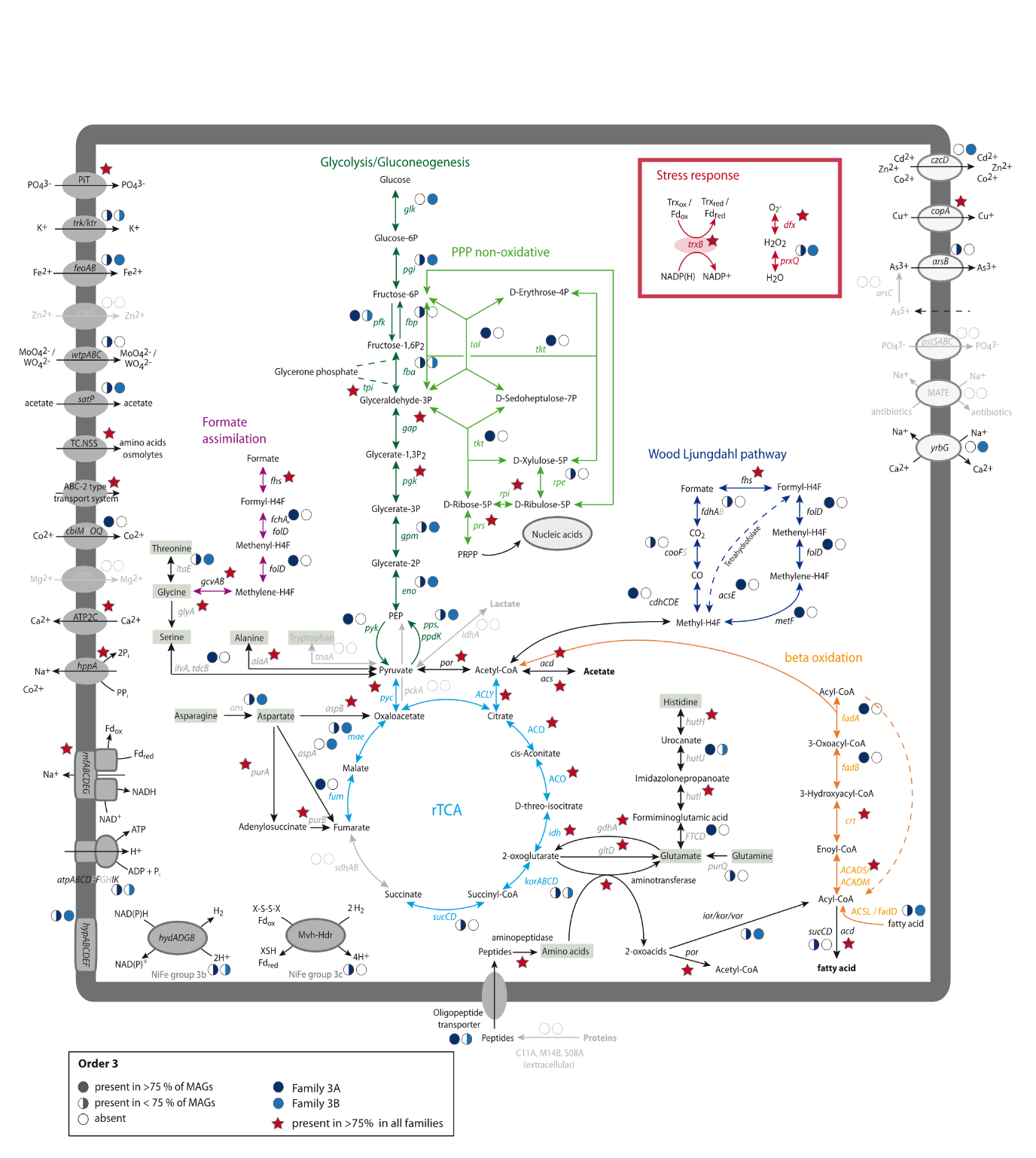
**

**S14. Metabolic reconstruction of Order 3 (Family 3A, Family 3B).** Pathways in grey represent pathways with missing genes that are therefore not functional. Gene abbreviations can be found in Supplementary table 9. The presence of genes is indicated by full or half circles for each family. Red stars indicate the presence of genes in all families.

**
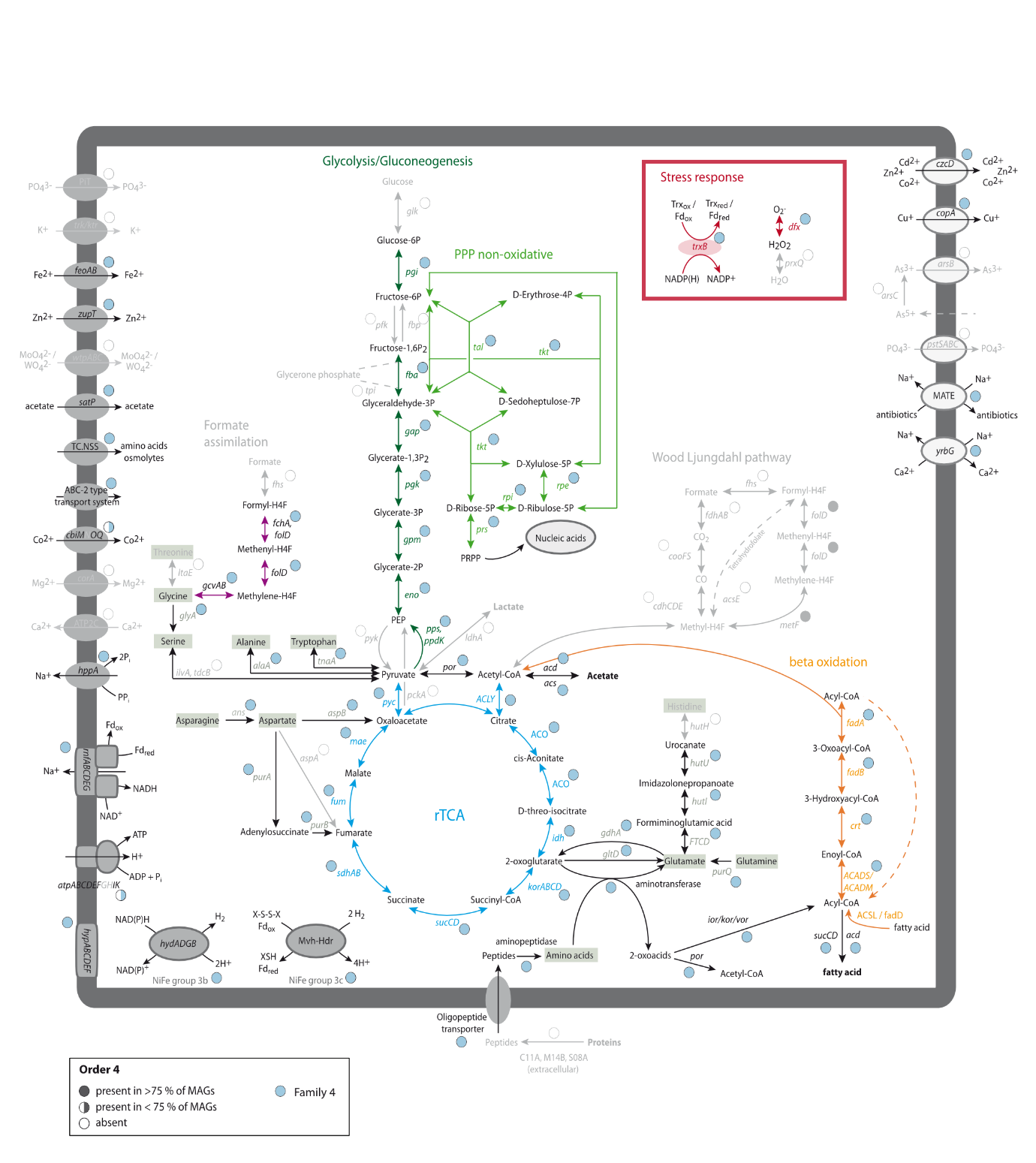
**

**S15. Metabolic reconstruction of Order 4 (Family 4).** Pathways in grey represent pathways with missing genes and are therefore not functional. Gene abbreviations can be found in Supplementary table 9. The presence of genes is indicated by full or half circles.

**
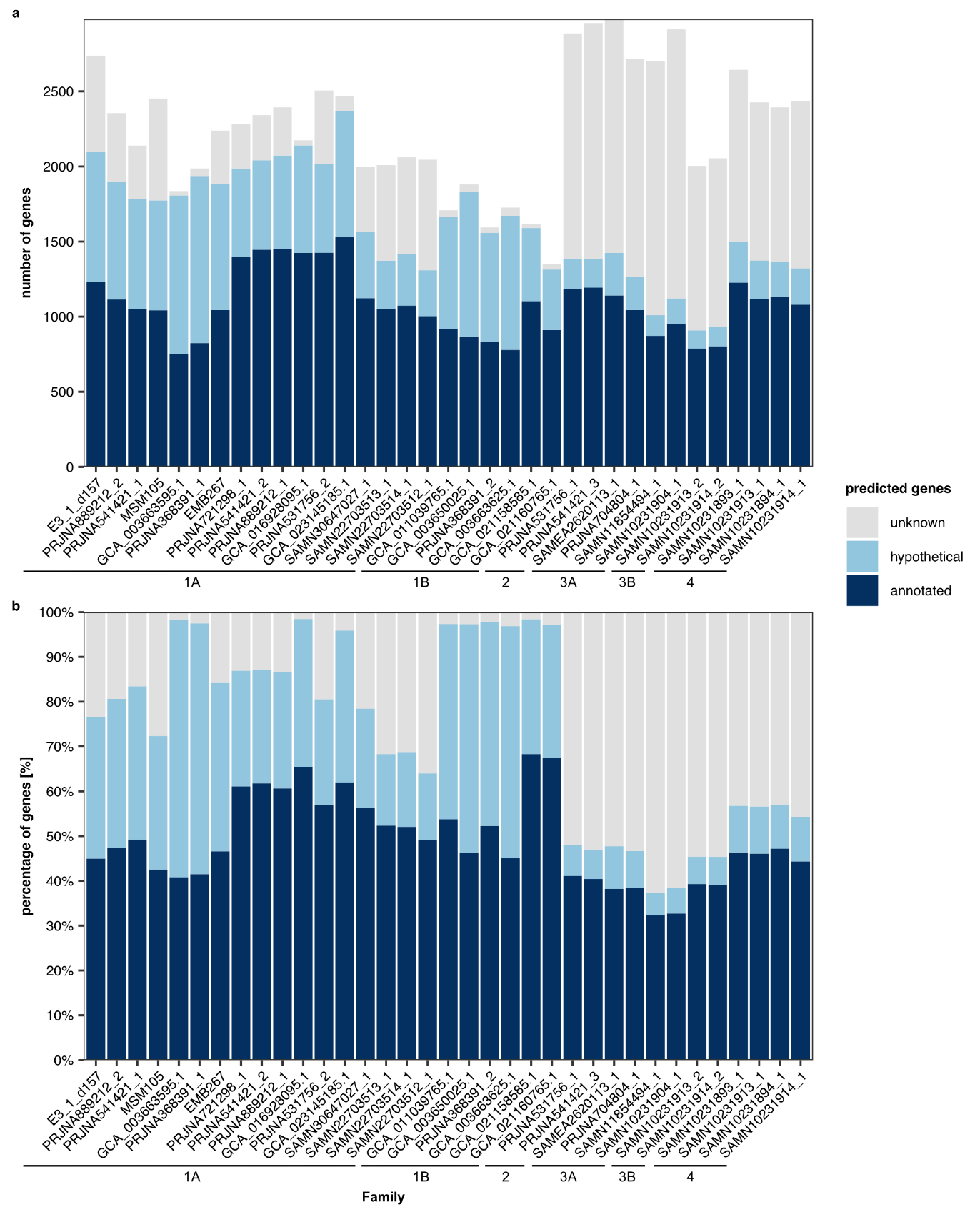
**

**S16. Annotation status of predicted genes in *Ca.* Penumbrarchaeia** **MAGs.** Genes classified as annotated, hypothetical and unknown based on NR and KEGG annotations shown as (**a.)** number of genes and (**b.)** percentage of genes. MAGs were ordered according to their family taxonomy in the marker gene tree (Figure 3). Family 1A corresponds to *Ca.* Penumbrarchaeaceae.

**
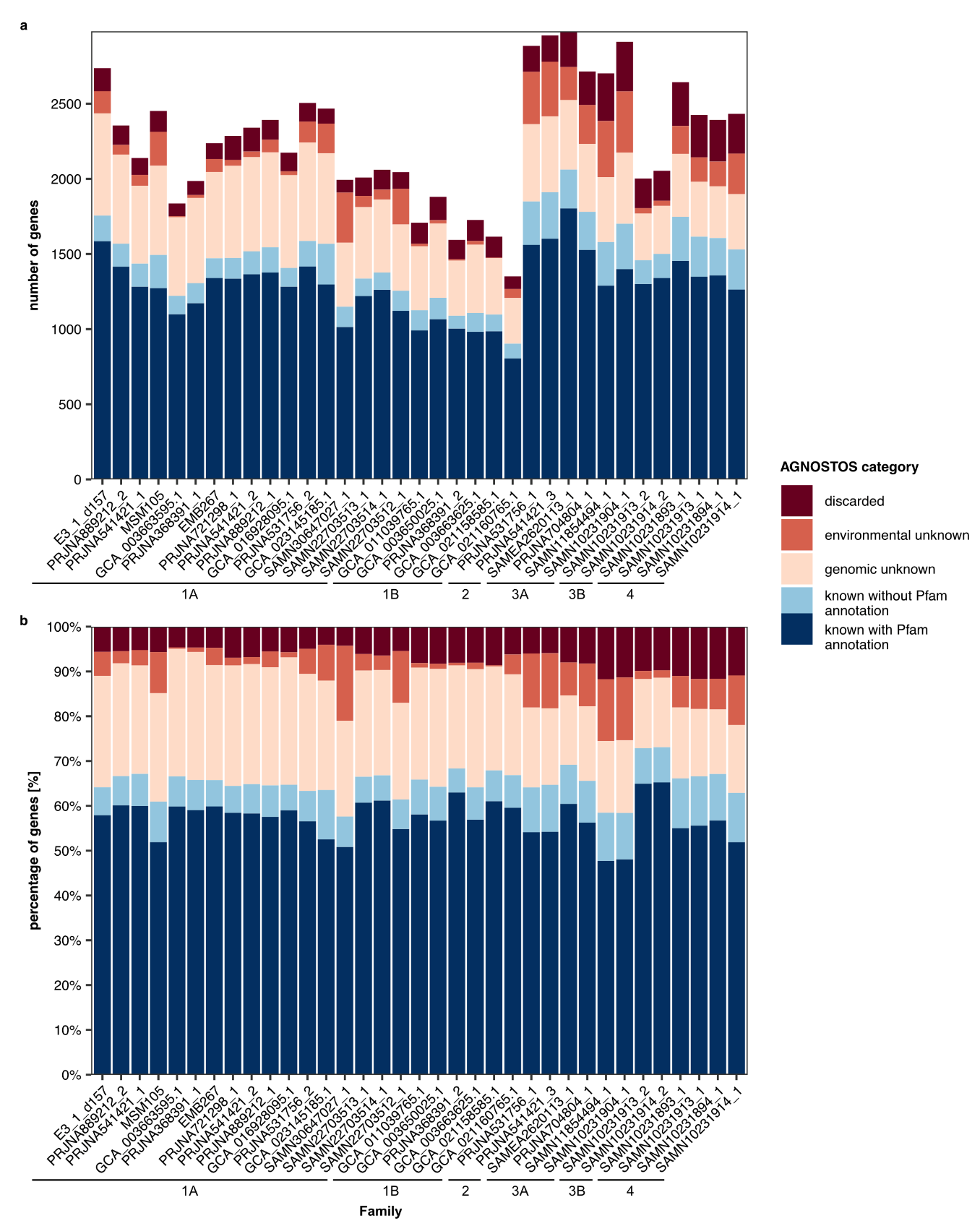
**

**S17. AGNOSTOS annotation category of genes in *Ca.* Penumbrarchaeia** **MAGs.** Gene classification based on AGNOSTOS. Gene classifications are shown as (**a.)** number of genes and (**b.)** percentage of genes. MAGs were ordered according to their family taxonomy in the marker gene tree (Figure 3). Family 1A corresponds to *Ca.* Penumbrarchaeaceae.

**
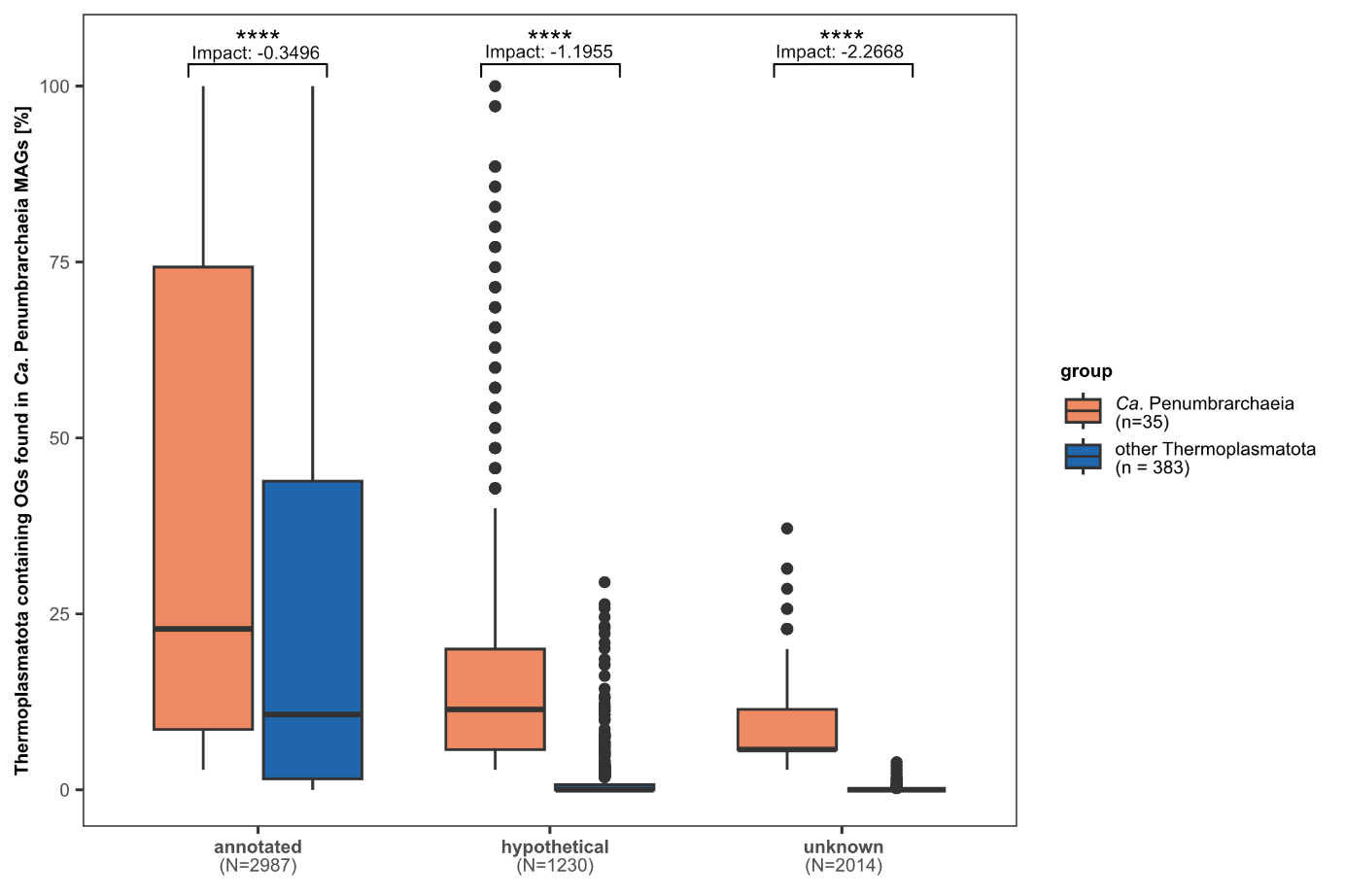
**

**S18. Shared orthogroups among Thermoplasmatota.** Boxplot of Thermoplasmatota genomes sharing orthogroups (OGs) present within *Ca.* Penumbrarchaeia MAGs separated by annotation category: annotated, hypothetical and unknown, according to their NR and KEGG annotation. Differences between groups were tested by Wilcoxon Signed Rank test, *p*-values are indicated by asterisks (**** *p* <= 0.0001). The measure of impact describes the effect size. Number of observations N indicates the number of orthogroups in each category. Number of genomes per group is indicated by number n.

**
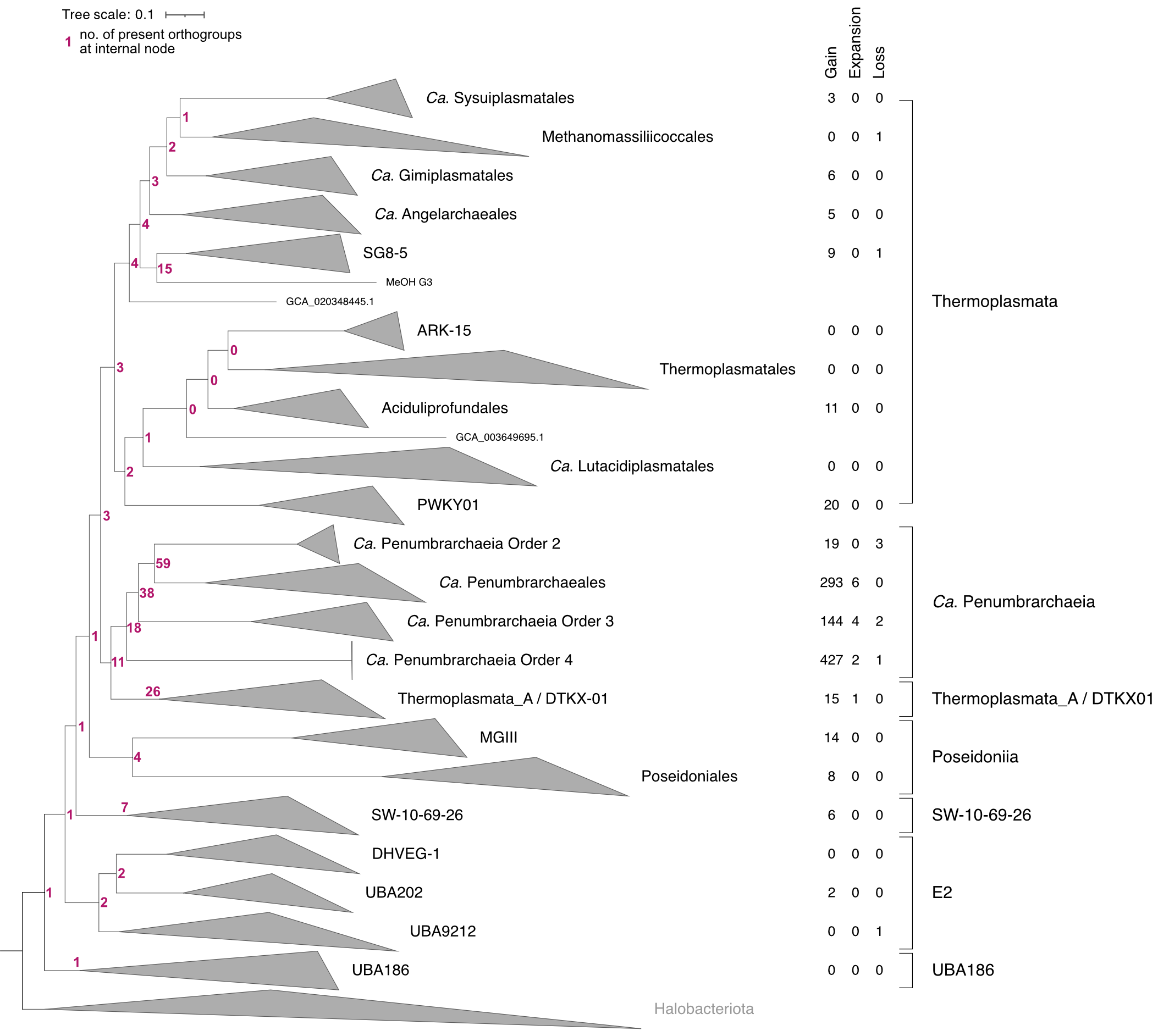
**

**S19. Ancestral gene family reconstruction of orthogroups containing genes with hypothetical or unknown function.** Evolutionary events are indicated for each order within the phylum of Thermoplasmatota representing orthogroup gains, expansions and losses. Evolutionary events were inferred using the Wagner parsimony in COUNT (gain penalty = 1) [99].

**
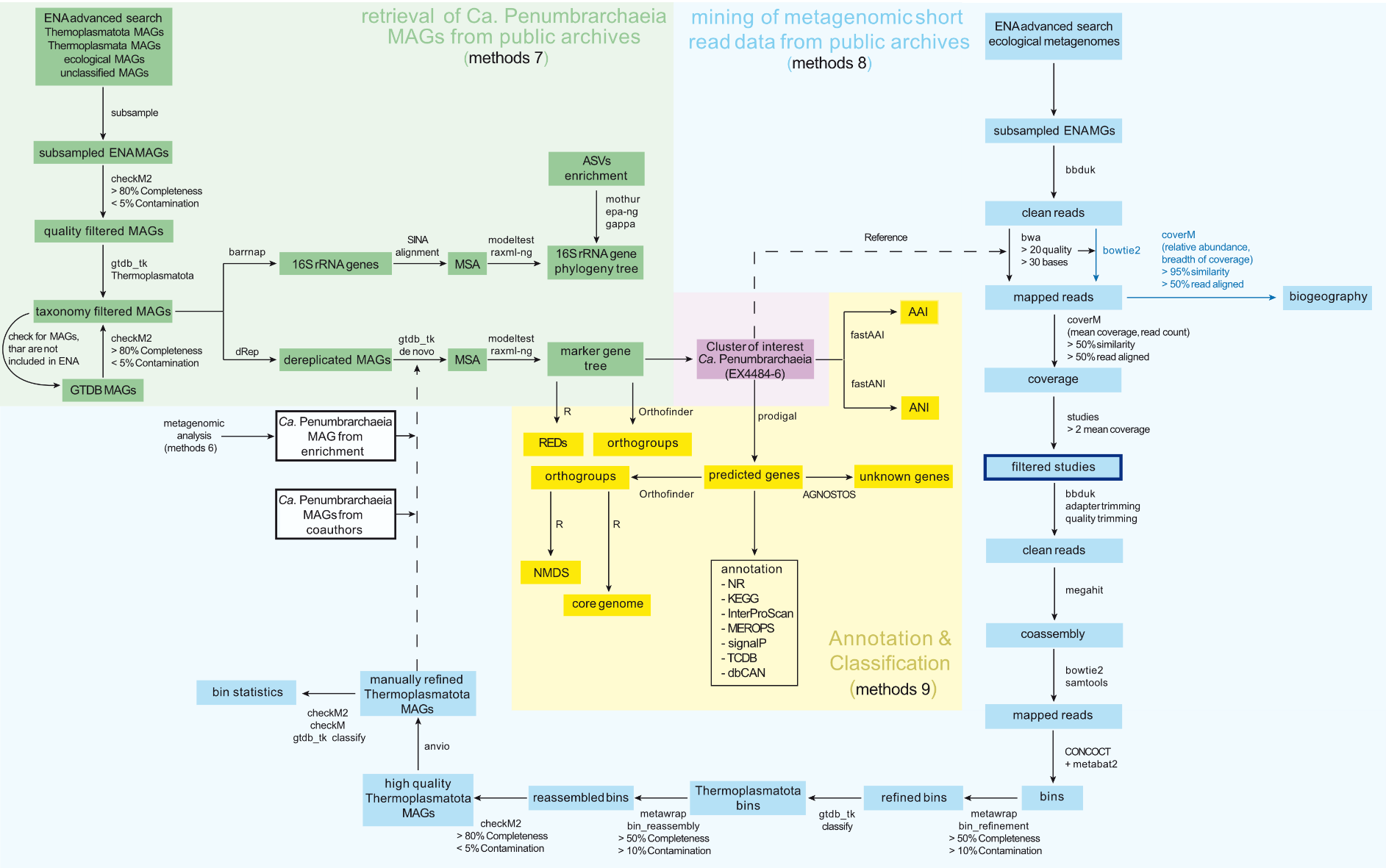
**

**S20.** **Flowchart of methods applied during the data mining conducted in this study.** Retrieval of *Ca.* Penumbrarchaeia (EX4484-6) MAGs from public archives (methods section 7) is displayed in green, the subsequent mining of metagenomic short read data from public archives is displayed in blue (methods section 8). Annotation and classification (methods section 9) was conducted on both, the MAGs retrieved from public archives and MAGs reconstructed from metagenomic short read data (yellow). Programs and settings used are indicated next to or below arrows.

## **Supplementary References**

1. Hedlund BP, Chuvochina M, Hugenholtz P, Konstantinidis KT, Murray AE, Palmer M, et al. SeqCode: a nomenclatural code for prokaryotes described from sequence data. Nat Microbiol. 2022;7(10):1702-8; doi: 10.1038/s41564-022-01214-9.

2. Bowers RM, Kyrpides NC, Stepanauskas R, Harmon-Smith M, Doud D, Reddy TBK, et al. Minimum information about a single amplified genome (MISAG) and a metagenome-assembled genome (MIMAG) of bacteria and archaea. Nat Biotechnol. 2017;35(8):725-31; doi: 10.1038/nbt.3893.

3. Grosskopf R, Janssen PH, Liesack W. Diversity and structure of the methanogenic community in anoxic rice paddy soil microcosms as examined by cultivation and direct 16S rRNA gene sequence retrieval. Appl Environ Microbiol. 1998;64(3):960-9; doi: 10.1128/aem.64.3.960-969.1998.

4. Lueders T, Friedrich MW. Effects of amendment with ferrihydrite and gypsum on the structure and activity of methanogenic populations in rice field soil. Appl Environ Microbiol. 2002;68(5):2484-94; doi: 10.1128/aem.68.5.2484-2494.2002.

5. Pruesse E, Peplies J, Glöckner FO. SINA: accurate high-throughput multiple sequence alignment of ribosomal RNA genes. Bioinformatics. 2012;28(14):1823-9; doi: 10.1093/bioinformatics/bts252.

6. Ludwig W, Strunk O, Westram R, Richter L, Meier H, Yadhukumar, et al. ARB: a software environment for sequence data. Nucleic Acids Res. 2004;32(4):1363-71; doi: 10.1093/nar/gkh293.

7. Yilmaz P, Parfrey LW, Yarza P, Gerken J, Pruesse E, Quast C, et al. The SILVA and “All-species Living Tree Project (LTP)” taxonomic frameworks. Nucleic Acids Res. 2013;42(D1):D643-D8; doi: 10.1093/nar/gkt1209.

8. Quast C, Pruesse E, Yilmaz P, Gerken J, Schweer T, Yarza P, et al. The SILVA ribosomal RNA gene database project: improved data processing and web-based tools. Nucleic Acids Res. 2013;41(Database issue):D590-6; doi: 10.1093/nar/gks1219.

9. Walters WA, Caporaso JG, Lauber CL, Berg-Lyons D, Fierer N, Knight R. PrimerProspector: de novo design and taxonomic analysis of barcoded polymerase chain reaction primers. Bioinformatics. 2011;27(8):1159-61; doi: 10.1093/bioinformatics/btr087.

10. Yin X, Cai M, Liu Y, Zhou G, Richter-Heitmann T, Aromokeye DA, et al. Subgroup level differences of physiological activities in marine Lokiarchaeota. ISME J. 2020;15(3):848-61; doi: 10.1038/s41396-020-00818-5.

11. Seemann T: barrnap 0.9 : rapid ribosomal RNA prediction. In.; 2018.

12. Darriba D, Posada D, Kozlov AM, Stamatakis A, Morel B, Flouri T. ModelTest-NG: a new and scalable tool for the selection of DNA and protein evolutionary models. Mol Biol Evol. 2019;37(1):291-4; doi: 10.1093/molbev/msz189.

13. Kozlov AM, Darriba D, Flouri T, Morel B, Stamatakis A. RAxML-NG: a fast, scalable and user-friendly tool for maximum likelihood phylogenetic inference. Bioinformatics. 2019;35(21):4453-5; doi: 10.1093/bioinformatics/btz305.

14. Schloss PD, Westcott SL, Ryabin T, Hall JR, Hartmann M, Hollister EB, et al. Introducing mothur: open-source, platform-independent, community-supported software for describing and comparing microbial communities. Appl Environ Microbiol. 2009;75(23):7537-41; doi: doi:10.1128/AEM.01541-09.

15. Barbera P, Kozlov AM, Czech L, Morel B, Darriba D, Flouri T, et al. EPA-ng: Massively Parallel Evolutionary Placement of Genetic Sequences. Syst Biol. 2018;68(2):365-9; doi: 10.1093/sysbio/syy054.

16. Czech L, Barbera P, Stamatakis A. Genesis and Gappa: processing, analyzing and visualizing phylogenetic (placement) data. Bioinformatics. 2020;36(10):3263-5; doi: 10.1093/bioinformatics/btaa070.

17. Altschul SF, Gish W, Miller W, Myers EW, Lipman DJ. Basic local alignment search tool. J Mol Biol. 1990;215(3):403-10; doi: 10.1016/s0022-2836(05)80360-2.

18. Paoli L, Ruscheweyh H-J, Forneris CC, Hubrich F, Kautsar S, Bhushan A, et al. Biosynthetic potential of the global ocean microbiome. Nature. 2022;607(7917):111-8; doi: 10.1038/s41586-022-04862-3.

19. Bushnell B. BBMap: a fast, accurate, splice-aware aligner. Lawrence Berkeley National Laboratory. 2014;LBNL Report #: LBNL-7065E.

20. Prjibelski A, Antipov D, Meleshko D, Lapidus A, Korobeynikov A. Using SPAdes de novo assembler. Curr Protoc Bioinformatics. 2020;70(1):e102; doi: 10.1002/cpbi.102.

21. Li H, Durbin R. Fast and accurate short read alignment with Burrows-Wheeler transform. Bioinformatics. 2009;25(14):1754-60; doi: 10.1093/bioinformatics/btp324.

22. Kang DD, Li F, Kirton E, Thomas A, Egan R, An H, et al. MetaBAT 2: an adaptive binning algorithm for robust and efficient genome reconstruction from metagenome assemblies. PeerJ. 2019;7:e7359; doi: 10.7717/peerj.7359.

23. Parks DH, Imelfort M, Skennerton CT, Hugenholtz P, Tyson GW. CheckM: assessing the quality of microbial genomes recovered from isolates, single cells, and metagenomes. Genome Res. 2015;25(7):1043-55; doi: 10.1101/gr.186072.114.

24. Eren AM, Kiefl E, Shaiber A, Veseli I, Miller SE, Schechter MS, et al. Community-led, integrated, reproducible multi-omics with anvi’o. Nat Microbiol. 2021;6(1):3-6; doi: 10.1038/s41564-020-00834-3.

25. Chaumeil P-A, Mussig AJ, Hugenholtz P, Parks DH. GTDB-Tk v2: memory friendly classification with the Genome Taxonomy Database. bioRxiv. 2022; doi: 10.1101/2022.07.11.499641.

26. Parks DH, Chuvochina M, Waite DW, Rinke C, Skarshewski A, Chaumeil P-A, et al. A standardized bacterial taxonomy based on genome phylogeny substantially revises the tree of life. Nat Biotechnol. 2018;36(10):996-1004; doi: 10.1038/nbt.4229.

27. Ocean Microbiomics Database. https://microbiomics.io/ocean2 (2024). Accessed 05 Sept 2024.

28. GTDB - Release 207 statistics. https://gtdb.ecogenomic.org/stats/r207#relative-evolutionary-divergence (2022). Accessed 04 April 2025.

29. GTDB - Release 214 statistics. https://gtdb.ecogenomic.org/stats/r214#relative-evolutionary-divergence (2023). Accessed 04 April 2025.

30. Konstantinidis K, Ruiz Pérez C, Gerhardt K, Rodríguez-R L, Jain C, Tiedje J, et al. FastAAI: efficient estimation of genome average amino acid identity and phylum-level relationships using tetramers of universal proteins. Preprint from Research Square. 2022; doi: 10.21203/rs.3.rs-1459378/v1.

31. Rudnick G, Krämer R, Blakely RD, Murphy DL, Verrey F. The SLC6 transporters: perspectives on structure, functions, regulation, and models for transporter dysfunction. Pflug Arch Eur J Phy. 2014;466(1):25-42; doi: 10.1007/s00424-013-1410-1.

32. Cantarel BL, Coutinho PM, Rancurel C, Bernard T, Lombard V, Henrissat B. The Carbohydrate-Active EnZymes database (CAZy): an expert resource for Glycogenomics. Nucleic Acids Res. 2009;37(Database issue):D233-8; doi: 10.1093/nar/gkn663.

33. López-Mondéjar R, Tláskal V, da Rocha UN, Baldrian P. Global distribution of carbohydrate utilization potential in the prokaryotic tree of life. mSystems. 2022;7(6):e0082922; doi: 10.1128/msystems.00829-22.

34. Mall A, Sobotta J, Huber C, Tschirner C, Kowarschik S, Bačnik K, et al. Reversibility of citrate synthase allows autotrophic growth of a thermophilic bacterium. Science. 2018;359(6375):563-7; doi: 10.1126/science.aao2410.

35. Garritano AN, Song W, Thomas T. Carbon fixation pathways across the bacterial and archaeal tree of life. PNAS Nexus. 2022;1(5); doi: 10.1093/pnasnexus/pgac226.

36. Yin X, Zhou G, Cai M, Zhu Q-Z, Richter-Heitmann T, Aromokeye DA, et al. Catabolic protein degradation in marine sediments confined to distinct archaea. ISME J. 2022; doi: 10.1038/s41396-022-01210-1.

37. Baltscheffsky M, Schultz A, Baltscheffsky H. H+-PPases: a tightly membrane-bound family. FEBS Lett. 1999;457(3):527-33; doi: 10.1016/S0014-5793(99)90617-8.

38. Belogurov GA, Malinen AM, Turkina MV, Jalonen U, Rytkönen K, Baykov AA, et al. Membrane-bound pyrophosphatase of *Thermotoga maritima* requires sodium for activity. Biochem. 2005;44(6):2088-96; doi: 10.1021/bi048429g.

39. Malinen AM, Belogurov GA, Baykov AA, Lahti R. Na+-pyrophosphatase:  a novel primary sodium pump. Biochem. 2007;46(30):8872-8; doi: 10.1021/bi700564b.

40. Kuhns M, Trifunović D, Huber H, Müller V. The Rnf complex is a Na^+^ coupled respiratory enzyme in a fermenting bacterium, *Thermotoga maritima*. Commun Biol. 2020;3(1):431; doi: 10.1038/s42003-020-01158-y.

41. Watanabe S, Sasaki D, Tominaga T, Miki K. Structural basis of [NiFe] hydrogenase maturation by Hyp proteins. Biol Chem. 2012;393(10):1089-100; doi: 10.1515/hsz-2012-0197.

42. Ma K, Weiss R, Adams MWW. Characterization of hydrogenase II from the hyperthermophilic archaeon *Pyrococcus furiosus* and assessment of its role in sulfur reduction. J Bacteriol. 2000;182(7):1864-71; doi: doi:10.1128/jb.182.7.1864-1871.2000.

43. Ma K, Schicho RN, Kelly RM, Adams MW. Hydrogenase of the hyperthermophile *Pyrococcus furiosus* is an elemental sulfur reductase or sulfhydrogenase: evidence for a sulfur-reducing hydrogenase ancestor. Proc Natl Acad Sci U S A. 1993;90(11):5341-4; doi: 10.1073/pnas.90.11.5341.

44. Ma K, Weiss R, Adams MW. Characterization of hydrogenase II from the hyperthermophilic archaeon *Pyrococcus furiosus* and assessment of its role in sulfur reduction. J Bacteriol. 2000;182(7):1864-71; doi: 10.1128/jb.182.7.1864-1871.2000.

45. Hedderich R, Berkessel A, Thauer RK. Purification and properties of heterodisulfide reductase from *Methanobacterium thermoautotrophicum* (strain Marburg). Eur J Biochem. 1990;193(1):255-61; doi: 10.1111/j.1432-1033.1990.tb19331.x.

46. Setzke E, Hedderich R, Heiden S, Thauer RK. H_2_: heterodisulfide oxidoreductase complex from *Methanobacterium thermoautotrophicum*. Composition and properties. Eur J Biochem. 1994;220(1):139-48; doi: 10.1111/j.1432-1033.1994.tb18608.x.

47. Thauer RK, Kaster AK, Seedorf H, Buckel W, Hedderich R. Methanogenic archaea: ecologically relevant differences in energy conservation. Nat Rev Microbiol. 2008;6(8):579-91; doi: 10.1038/nrmicro1931.

48. Kaster AK, Moll J, Parey K, Thauer RK. Coupling of ferredoxin and heterodisulfide reduction via electron bifurcation in hydrogenotrophic methanogenic archaea. Proc Natl Acad Sci U S A. 2011;108(7):2981-6; doi: 10.1073/pnas.1016761108.

49. Imachi H, Nobu MK, Nakahara N, Morono Y, Ogawara M, Takaki Y, et al. Isolation of an archaeon at the prokaryote-eukaryote interface. Nature. 2020;577(7791):519-25; doi: 10.1038/s41586-019-1916-6.

50. Qu Y-N, Rao Y-Z, Qi Y-L, Li Y-X, Li A, Palmer M, et al. *Panguiarchaeum symbiosum*, a potential hyperthermophilic symbiont in the TACK superphylum. Cell Rep. 2023;42(3):112158; doi: 10.1016/j.celrep.2023.112158.

51. Sapra R, Verhagen MFJM, Adams MWW. Purification and characterization of a membrane-bound hydrogenase from the hyperthermophilic archaeon *Pyrococcus furiosus*. J Bacteriol. 2000;182(12):3423-8; doi: 10.1128/jb.182.12.3423-3428.2000.

52. Lau CK, Ishida H, Liu Z, Vogel HJ. Solution structure of *Escherichia coli* FeoA and its potential role in bacterial ferrous iron transport. J Bacteriol. 2013;195(1):46-55; doi: 10.1128/jb.01121-12.

53. Schulz H, Zabel M: Marine geochemistry. In., 2nd ed. edn. Berlin Heidelberg, Germany: Springer-Verlag Berlin Heidelberg; 2006.

54. Silburn B, Kröger S, Parker ER, Sivyer DB, Hicks N, Powell CF, et al. Benthic pH gradients across a range of shelf sea sediment types linked to sediment characteristics and seasonal variability. Biogeochemistry. 2017;135(1):69-88; doi: 10.1007/s10533-017-0323-z.

55. Von Damm KL, Edmond JM, Measures CI, Grant B. Chemistry of submarine hydrothermal solutions at Guaymas Basin, Gulf of California. Geochim Cosmochim Acta. 1985;49(11):2221-37; doi: 10.1016/0016-7037(85)90223-6.

56. Ulfsbo A, Hulth S, Anderson LG. pH and biogeochemical processes in the Gotland Basin of the Baltic Sea. Mar Chem. 2011;127(1):20-30; doi: 10.1016/j.marchem.2011.07.004.

57. Kammler M, Schön C, Hantke K. Characterization of the ferrous iron uptake system of *Escherichia coli*. J Bacteriol. 1993;175(19):6212-9; doi: 10.1128/jb.175.19.6212-6219.1993.

58. Andreini C, Banci L, Bertini I, Rosato A. Zinc through the three domains of life. J Proteome Res. 2006;5(11):3173-8; doi: 10.1021/pr0603699.

59. Grass G, Franke S, Taudte N, Nies DH, Kucharski LM, Maguire ME, et al. The metal permease ZupT from *Escherichia coli* is a transporter with a broad substrate spectrum. J Bacteriol. 2005;187(5):1604-11; doi: doi:10.1128/jb.187.5.1604-1611.2005.

60. Pyle AM. Role of metal ions in ribozymes. Met Ions Biol Syst. 1996;32:479-520.

61. Wolf FI, Cittadini A. Chemistry and biochemistry of magnesium. Mol Aspects Med. 2003;24(1-3):3-9; doi: 10.1016/s0098-2997(02)00087-0.

62. Smith RL, Banks JL, Snavely MD, Maguire ME. Sequence and topology of the CorA magnesium transport systems of Salmonella typhimurium and *Escherichia coli*. Identification of a new class of transport protein. J Biol Chem. 1993;268(19):14071-80; doi: 10.1016/S0021-9258(19)85210-9.

63. Smith RL, Maguire ME. Microbial magnesium transport: unusual transporters searching for identity. Mol Microbiol. 1998;28(2):217-26; doi: 10.1046/j.1365-2958.1998.00810.x.

64. Boer JL, Mulrooney SB, Hausinger RP. Nickel-dependent metalloenzymes. Arch Biochem Biophys. 2014;544:142-52; doi: 10.1016/j.abb.2013.09.002.

65. Kobayashi M, Shimizu S. Cobalt proteins. Eur J Biochem. 1999;261(1):1-9; doi: 10.1046/j.1432-1327.1999.00186.x.

66. Nakata A, Amemura M, Shinagawa H. Regulation of the phosphate regulon in *Escherichia coli* K-12: regulation of the negative regulatory gene phoU and identification of the gene product. J Bacteriol. 1984;159(3):979-85; doi: 10.1128/jb.159.3.979-985.1984.

67. Gardner SG, Johns KD, Tanner R, McCleary WR. The PhoU Protein from *Escherichia coli* Interacts with PhoR, PstB, and metals to form a phosphate-signaling complex at the membrane. J Bacteriol. 2014;196(9):1741-52; doi: doi:10.1128/jb.00029-14.

68. Muda M, Rao NN, Torriani A. Role of PhoU in phosphate transport and alkaline phosphatase regulation. J Bacteriol. 1992;174(24):8057-64; doi: doi:10.1128/jb.174.24.8057-8064.1992.

69. Stautz J, Hellmich Y, Fuss MF, Silberberg JM, Devlin JR, Stockbridge RB, et al. Molecular mechanisms for bacterial potassium homeostasis. J Mol Biol. 2021;433(16):166968; doi: 10.1016/j.jmb.2021.166968.

70. Beagle SD, Lockless SW. Unappreciated roles for K(+) channels in bacterial physiology. Trends Microbiol. 2021;29(10):942-50; doi: 10.1016/j.tim.2020.11.005.

71. Cao Y, Pan Y, Huang H, Jin X, Levin EJ, Kloss B, et al. Gating of the TrkH ion channel by its associated RCK protein TrkA. Nature. 2013;496(7445):317-22; doi: 10.1038/nature12056.

72. Bevers LE, Hagedoorn PL, Krijger GC, Hagen WR. Tungsten transport protein A (WtpA) in *Pyrococcus furiosus*: the first member of a new class of tungstate and molybdate transporters. J Bacteriol. 2006;188(18):6498-505; doi: 10.1128/jb.00548-06.

73. Makdessi K, Andreesen JR, Pich A. Tungstate uptake by a highly specific ABC transporter in *Eubacterium acidaminophilum*. J Biol Chem. 2001;276(27):24557-64; doi: 10.1074/jbc.M101293200.

74. Hille R. Molybdenum and tungsten in biology. Trends Biochem Sci. 2002;27(7):360-7; doi: 10.1016/s0968-0004(02)02107-2.

75. Kletzin A, Adams MW. Tungsten in biological systems. FEMS Microbiol Rev. 1996;18(1):5-63; doi: 10.1016/0168-6445(95)00025-9.

76. Møller JV, Juul B, le Maire M. Structural organization, ion transport, and energy transduction of P-type ATPases. Biochim Biophys Acta Rev Biomembr. 1996;1286(1):1-51; doi: 10.1016/0304-4157(95)00017-8.

77. Sá-Pessoa J, Paiva S, Ribas D, Silva IJ, Viegas SC, Arraiano CM, et al. SATP (YaaH), a succinate-acetate transporter protein in *Escherichia coli*. Biochem J. 2013;454(3):585-95; doi: 10.1042/bj20130412.

78. Bernal V, Castaño-Cerezo S, Cánovas M. Acetate metabolism regulation in *Escherichia coli*: carbon overflow, pathogenicity, and beyond. Appl Microbiol Biotechnol. 2016;100(21):8985-9001; doi: 10.1007/s00253-016-7832-x.

79. Riebe O, Fischer RJ, Bahl H. Desulfoferrodoxin of *Clostridium acetobutylicum* functions as a superoxide reductase. FEBS Lett. 2007;581(29):5605-10; doi: 10.1016/j.febslet.2007.11.008.

80. Perkins A, Nelson KJ, Parsonage D, Poole LB, Karplus PA. Peroxiredoxins: guardians against oxidative stress and modulators of peroxide signaling. Trends Biochem Sci. 2015;40(8):435-45; doi: 10.1016/j.tibs.2015.05.001.

81. Thompson AD, Bernard SM, Skiniotis G, Gestwicki JE. Visualization and functional analysis of the oligomeric states of *Escherichia coli* heat shock protein 70 (Hsp70/DnaK). Cell Stress Chaperones. 2012;17(3):313-27; doi: 10.1007/s12192-011-0307-1.

82. Delaney JM. Requirement of the *Escherichia coli* dnaK gene for thermotolerance and protection against H_2_O_2_. Microbiol. 1990;136(10):2113-8; doi: 10.1099/00221287-136-10-2113.

83. Besserer GM, Nicoll DA, Abramson J, Philipson KD. Characterization and purification of a Na^+^/Ca^2+^ exchanger from an archaebacterium. J Biol Chem. 2012;287(11):8652-9; doi: 10.1074/jbc.M111.331280.

84. Rensing C, Fan B, Sharma R, Mitra B, Rosen BP. CopA: an *Escherichia coli* Cu(I)-translocating P-type ATPase. Proc Natl Acad Sci U S A. 2000;97(2):652-6; doi: doi:10.1073/pnas.97.2.652.

85. Fan B, Rosen BP. Biochemical characterization of CopA, the *Escherichia coli* Cu(I)-translocating P-type ATPase. J Biol Chem. 2002;277(49):46987-92; doi: 10.1074/jbc.M208490200.

86. Peña MM, Lee J, Thiele DJ. A delicate balance: homeostatic control of copper uptake and distribution. J Nutr. 1999;129(7):1251-60; doi: 10.1093/jn/129.7.1251.

87. Linder MC. Introduction and overview of copper as an element essential for life. In: Biochemistry of Copper. Boston, MA: Springer US; 1991. p. 1-13.

88. Anton A, Grosse C, Reissmann J, Pribyl T, Nies DH. CzcD is a heavy metal ion transporter involved in regulation of heavy metal resistance in *Ralstonia* sp. strain CH34. J Bacteriol. 1999;181(22):6876-81; doi: 10.1128/jb.181.22.6876-6881.1999.

89. Rosen BP, Liu Z. Transport pathways for arsenic and selenium: a minireview. Environ Int. 2009;35(3):512-5; doi: 10.1016/j.envint.2008.07.023.

90. Cullen WR, Reimer KJ. Arsenic speciation in the environment. Chem Rev. 1989;89(4):713-64.

91. Kalia K, Khambholja DB. 28 - Arsenic contents and its biotransformation in the marine environment. In: Flora SJS, editor. Handbook of Arsenic Toxicology. Oxford: Academic Press; 2015. p. 675-700.

92. Knobloch T. Chemical Munitions Dumped in the Baltic Sea: Report of the Ad Hoc Expert Group to Update and Review the Existing Information on Dumped Chemical Munitions in the Baltic Sea (HELCOM MUNI). Helsinki Commission, Baltic Marine Environment Protection Commission; 2014.

93. Missiaen T, Söderström M, Popescu I, Vanninen P. Evaluation of a chemical munition dumpsite in the Baltic Sea based on geophysical and chemical investigations. Sci Total Environ. 2010;408(17):3536-53; doi: 10.1016/j.scitotenv.2010.04.056.

94. Szubska M, Bełdowski J. Spatial distribution of arsenic in surface sediments of the southern Baltic Sea. Oceanol. 2023;65(2):423-33; doi: 10.1016/j.oceano.2022.12.002.

95. Krah A, Huber RG, Zachariae U, Bond PJ. On the ion coupling mechanism of the MATE transporter ClbM. Biochim Biophys Acta Biomembr. 2020;1862(2):183137; doi: 10.1016/j.bbamem.2019.183137.

96. Bouki C, Venieri D, Diamadopoulos E. Detection and fate of antibiotic resistant bacteria in wastewater treatment plants: a review. Ecotoxicol Environ Saf. 2013;91:1-9; doi: 10.1016/j.ecoenv.2013.01.016.

97. Schijven JF, Blaak H, Schets FM, de Roda Husman AM. Fate of extended-spectrum β-lactamase-producing *Escherichia coli* from faecal sources in surface water and probability of human exposure through swimming. Environ Sci Technol. 2015;49(19):11825-33; doi: 10.1021/acs.est.5b01888.

98. Morel B, Schade P, Lutteropp S, Williams TA, Szöllősi GJ, Stamatakis A. SpeciesRax: A Tool for Maximum Likelihood Species Tree Inference from Gene Family Trees under Duplication, Transfer, and Loss. Mol Biol Evol. 2022;39(2); doi: 10.1093/molbev/msab365.

99. Csűös M. Count: evolutionary analysis of phylogenetic profiles with parsimony and likelihood. Bioinformatics. 2010;26(15):1910-2; doi: 10.1093/bioinformatics/btq315.
