# Supplementary figures and images for: Extensive data mining uncovers novel diversity among members of the rare biosphere within the Thermoplasmatota
Source: Microbiome. 2025 Jul 1;13:155. doi: 10.1186/s40168-025-02140-8 (PMC12220078; doi:10.1186/s40168-025-02140-8)

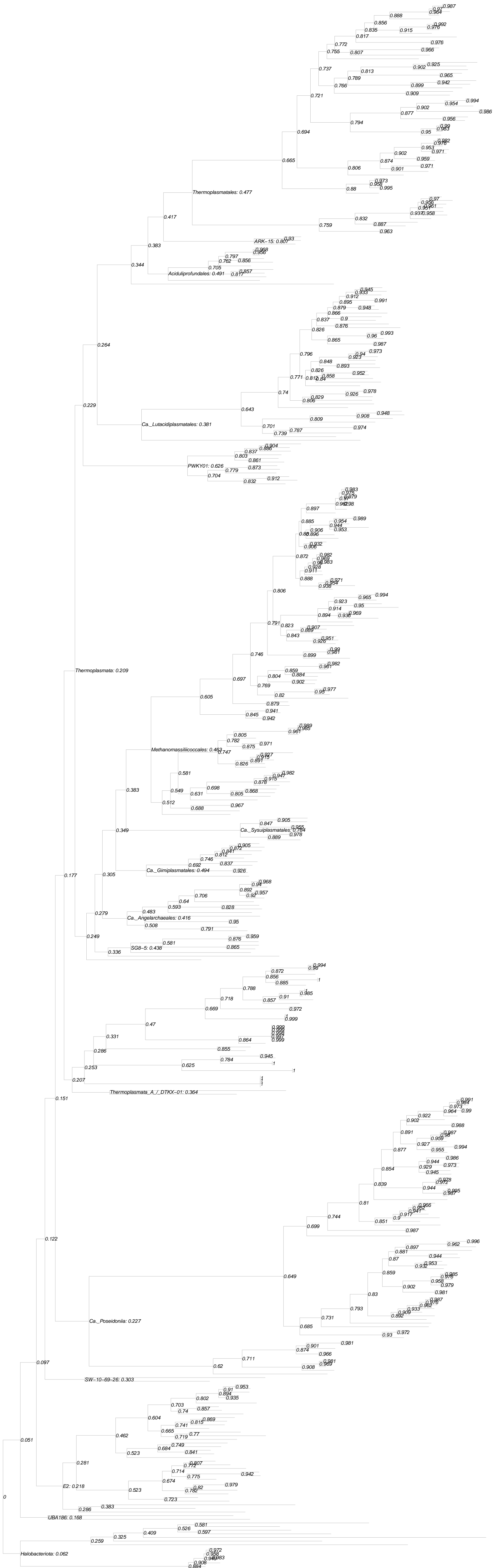

Supplement: Supplementary file 4 — Supplementary Material 3. Proposal of type material and higher ranks. Methods: I. Clone library construction. II. Standard preparation for qPCR. III. qPCR primer design. IV. 16S rRNA gene phylogenetic tree. V. Data collection, processing and MAG reconstruction in OMDB (v2). Results and discussion: I. Additional qPCR results. II. Delineation of the class Ca. Penumbrarchaeia. III. Annotation of the class Ca. Penumbrarchaeia. Carbon metabolism. Carbon assimilation. Hydrogenases and energy conservation. Transporters. Stress response. [file 40168_2025_2140_MOESM3_ESM.pdf]
